# Symmetry, gauge freedoms, and the interpretability of sequence-function relationships

**DOI:** 10.1101/2024.05.12.593774

**Published:** 2024-05-13

**Authors:** Anna Posfai, David M. McCandlish, Justin B. Kinney

**Affiliations:** 1 Simons Center for Quantitative Biology, Cold Spring Harbor Laboratory, Cold Spring Harbor, NY, 11724

**Keywords:** sequence-function relationships, gauge freedoms, sequence space sermutation symmetry, representation theory

## Abstract

Quantitative models of sequence-function relationships, which describe how biological sequences encode functional activities, are ubiquitous in modern biology. One important aspect of these models is that they commonly exhibit gauge freedoms, i.e., directions in parameter space that do not affect model predictions. In physics, gauge freedoms arise when physical theories are formulated in ways that respect fundamental symmetries. However, the connections that gauge freedoms in models of sequence-function relationships have to the symmetries of sequence space have yet to be systematically studied. Here we study the gauge freedoms of models that respect a specific symmetry of sequence space: the group of position-specific character permutations. We find that gauge freedoms arise when the transformations of model parameters that compensate for these symmetry transformations are described by redundant irreducible matrix representations. Based on this finding, we describe an “embedding distillation” procedure that enables analytic calculation of the dimension of the space of gauge freedoms, as well as efficient computation of a sparse basis for this space. Finally, we show that the ability to interpret model parameters as quantifying allelic effects places strong constraints on the form that models can take, and in particular show that all nontrivial equivariant models of allelic effects must exhibit gauge freedoms. Our work thus advances the understanding of the relationship between symmetries and gauge freedoms in quantitative models of sequence-function relationships.

## Introduction

Understanding the quantitative nature of sequence-function relationships is a major goal of modern biology (1). To study a specific sequence-function relationship of interest, researchers often propose a mathematical model, fit the parameters of the model to data, then biologically interpret the resulting parameter values. This interpretation step is often complicated, however, by gauge freedoms—directions in parameter space along which model parameters can be changed without altering model predictions. If any gauge freedoms are present in a model, the numerical values of individual model parameters cannot be meaningfully interpreted in the absence of additional constraints.

Researchers performing quantitative studies of sequence function relationships routinely encounter gauge freedoms in their models. In practice, one of two methods is typically used to overcome the difficulties that such gauge freedoms can present. One method—called “gauge fixing”—removes gauge freedoms by introducing additional constraints on model parameters (2–18). Another method limits the mathematical models that one uses to models that do not have any gauge freedoms (19–24). But despite being frequently encountered in the course of research, the gauge freedoms present in models of sequence-function relationships have received little attention (though see e.g. 3, 5–7, 12, 25). In particular, the mathematical properties of these gauge freedoms have yet to be sytematically studied.

In physics, by contrast, gauge freedoms are a topic of fundamental importance (26). Gauge freedoms are well-known to arise when a physical theory is expressed in a form that manifestly respects fundamental symmetries. For example, the classical theory of electricity and magnetism (E&M) is invariant to Lorentz transformations, i.e., changes in an observer’s velocity (27). Lorentz invariance is obscured, however, when the equations of E&M are expressed directly in terms of electric and magnetic fields. To express E&M in a form that is manifestly Lorentz invariant, one must instead formulate the equations in terms of an electromagnetic four-potential. Doing this introduces gauge freedoms because the four-potential, unlike electric and magnetic fields, is neither directly measurable nor uniquely determined by the configuration of a physical system^*^. Nevertheless, working with the four-potential greatly simplifies the equations of E&M and often aids in both their solution and their physical interpretation.

Motivated by the connection between gauge freedoms and symmetries in physics, we investigated whether the gauge freedoms in mathematical models of sequence-function relationships have a connection to the symmetries of sequence space. Here we study the gauge freedoms of linear models that are equivariant under a specific symmetry group of sequence space—the group of position-specific character permutations. These models include many of the most commonly used models, including models with pairwise and/or higher-order interactions. Using techniques from the theory of matrix representations, we find that the gauge freedoms of these models arise when model parameters transform under redundant irreducible matrix representations of the symmetry group. Based on this finding, we describe an “embedding distillation” procedure that facilitates the analysis of the vector space formed by the gauge freedoms of a large class of commonly used models.

Finally, we investigate the connection between parameter interpretability and model transformation behavior. We show that the ability to interpret the individual parameters of an equivariant model as quantifying the effects of specific alleles requires that these parameters transform under a permutation representation of the symmetry group, rather than a more general matrix representation. A consequence is that all nontrivial models that satisfy this interpretation criterion have gauge freedoms. This shows in particular that models that have gauge freedoms can have important advantages over mathematically equivalent models that do not have gauge freedoms. A companion paper (31) reports specific gauge-fixing strategies that can be applied to the most commonly used models that can be interpreted as quantifying allelic effects.

## Background

We now establish definitions and notation used in Results. We also review basic results regarding gauge freedoms in mathematical models of sequence-function relationships. Our companion paper (31) provides an expanded discussion of these results together with corresponding proofs.

### Sequence-function relationships.

Let 𝒜 denote an alphabet comprising α distinct characters. Let 𝒮 denote the set of $\alpha^{L}$ sequences of length L built from these characters. A model of a sequence-function relationship, $f(s;\vec{\theta })$, is defined to be a function that maps each sequence s∈𝒮 to a complex number. The vector $\vec{\theta }$ denotes the parameters of the model and is assumed to comprise M complex numbers.

### Linear models.

Linear models of sequence-function relationships are linear in $\vec{\theta }$ and thus have the form

[1] $$f(s;\vec{\theta })={\vec{\theta }}^{†}\vec{x}(s)=\sum_{i=1}^{M}\theta_{i}x_{i}(s),$$

where $\vec{x}(\cdot )$ is a vector of M distinct sequence features, and each feature $x_{i}(\cdot )$ is a function that maps sequences in 𝒮 to the complex numbers. We refer to the space $C^{M}$ in which these feature vectors live as feature space, and each specific feature vector $\vec{x}(s)$ as the embedding of sequence s.

Note that we let both sequence embeddings $\vec{x}$ and model parameters $\vec{\theta }$ be complex. By contrast, ref. (31) limited embeddings and parameters to the reals. We choose here to work in complex spaces because, in addition to the added generality of the results, the algebraic completeness of the complex numbers simplifies some of our proofs. All of our results, however, hold if the parameters and embeddings are restricted to the reals. See SI Sec. 10 for details.

### Generalized one-hot (GO) models.

GO models are linear models in which the sequence features indicate only the presence or absence of specific characters at specific positions (1). An example of a GO is the pairwise-interaction model, which has the form

[2] $$f_{\text{pair}}(s)=\theta_{0}x_{0}(s)+\sum_{l}\sum_{c}\theta_{l}^{c}x_{l}^{c}(s)+\sum_{l<l^{\prime }}\sum_{c,c^{\prime }}\theta_{ll^{\prime }}^{cc^{\prime }}x_{ll^{\prime }}^{cc^{\prime }}(s),$$

where $l,l^{\prime }\in \{1,\ldots ,L\}$ index positions within sequences s and $c,c^{\prime }\in 𝒜$ index characters at these positions. Pairwiseinteraction models comprise three types of GO feature: the constant feature, $x_{0}(s)$, which equals one for every sequence s; additive features, $x_{l}^{c}(s)$, which equal one if $s_{l}=c$ and equal zero otherwise (where $s_{l}$ denotes the character at position l in sequence s); and pairwise features, $x_{ll^{\prime }}^{{cc}^{\prime }}(s)$, which equal one if both $s_{l}=c$ and $s_{l^{\prime }}=c^{\prime }$, and which equal zero otherwise.

GO models are defined in a similar manner: as sums of terms that each have the form

[3] $$\theta_{l_{1}l_{2}\ldots l_{K}}^{c_{1}c_{2}\ldots c_{K}}x_{l_{1}l_{2}\ldots l_{K}}^{c_{1}c_{2}\ldots c_{K}}(s).$$

Here, K∈{0,…,L} is a term-specific number, $\left\{l_{1},l_{2},\ldots ,l_{K}\right\}$ is a term-specific set of positions, and $\left\{c_{1},c_{2},\ldots ,c_{K}\right\}$ is a term-specific set of characters at the corresponding positions. Each feature $x_{l_{1}l_{2}\ldots l_{K}}^{c_{1}c_{2}\ldots c_{K}}(s)$ is a K-order one-hot feature defined to be equal to one if $s_{k}=c_{k}$ for all k∈{1,…,K} and equal to zero otherwise. For example, the pairwise-interaction model is a GO model that contains a K=0 term^†^ as well as all possible terms of order K=1 and K=2.

### Gauge freedoms.

Gauge freedoms are transformations of model parameters that do not affect model predictions. Formally, a gauge freedom is any vector $\vec{g}\in C^{M}$ that satisfies

[4] $$f(s;\vec{\theta })=f(s;\vec{\theta }+\vec{g})\;\text{for}\;\text{all}\;s\in 𝒮.$$

For linear sequence-function relationships, the set of gauge freedoms G is a vector space in $C^{M}.\;G$ is the orthogonal complement of the space spanned by sequence embeddings, which we denote by $span\vec{x}$ (31). In what follows, we use γ to represent the dimension of G, and often refer to this quantity somewhat informally as the number of gauge freedoms.

Gauge freedoms arise from linear dependencies among sequence features. For example, one-hot pairwise-interaction models have M=1+αL+L2α2 parameters, but span $\vec{x}$ has only 1+(α−1)L+L2(α−1)2 dimensions due the presence of L+L2(2α−1) constraints on the embedding. Specifically, $x_{0}(s)=\sum_{c^{\prime }}x_{l}^{c^{\prime }}(s)$ for all positions l (yielding 1 constraint per position), and both $x_{l}^{c}(s)=\sum_{c^{\prime }}x_{ll^{\prime }}^{{cc}^{\prime }}(s)$ and $x_{l^{\prime }}^{c}(s)=\sum_{c^{\prime }}x_{ll^{\prime }}^{c^{\prime }c}(s)$ for all characters c and for all pairs of positions $l<l^{\prime }$ [yielding 2α−1 independent constraints per pair of positions (31)]. The one-hot pairwise interaction model therefore has γ=L+L2(2α−1) gauge freedoms; See also (3, 5, 7, 10).

### Fixing the gauge.

Fixing the gauge is the process of removing gauge freedoms by restricting $\vec{\theta }$ to a subset of parameter space, Θ, called the gauge. Linear gauges are choices of Θ that are themselves vector spaces. One useful property of linear gauges is that gauge-fixing can be accomplished by projection. Specifically, for any linear gauge Θ, there exists a projection matrix P that projects each parameter vector $\vec{\theta }\in C^{M}$ onto an equivalent parameter vector ${\vec{\theta }}_{\text{fixed}}$ that lies in Θ, i.e.,

[5] $${\vec{\theta }}_{\text{fixed}}=P\vec{\theta }.$$

Given Θ, the projection matrix P is uniquely defined by the requirement that P is idempotent, the image P is Θ, and the kernel of P is G. Our companion paper (31) describes a parametric family of linear gauges (including an explicit formula for the projection matrix) that includes many of the most commonly used gauges as special cases.

## Results

In what follows, we define the group of position-specific character permutations, as well as the linear models that are equivariant under this group. Next, we use methods from the theory of group representations (32) to identify all possible equivariant linear models. In the process, we also describe a porocedure we call “embedding distillation” that allows one to compute the gauge freedoms of any equivariant linear model. After demonstrating embedding distillation on the one-hot pairwise-interaction model, we apply embedding distillation to GEO models and derive expressions for the number of gauge freedoms (i.e., the dimension of the space of gage freedoms) of a variety of commonly used models. Finally, we explore the relationship between model transformation behavior and parameter identifiability.

### Position-specific character permutations.

Let $H_{CP}^{l}$ denote the group of permutations among the α possible characters at position l in a sequence. Note that $H_{CP}^{l}$ is isomorphic to the symmetric group on α elements, $S_{\alpha }$ (32). The group of position-specific character permutations is given by the direct product of all $H_{CP}^{l}$, i.e.,

[6] $$H_{PSCP}=H_{CP}^{1}\times \cdots \times H_{CP}^{L}.$$

Given any $h\in H_{PSCP}$, the transformation of a sequence s by h is written as hs, and the transformation of sequence space 𝒮 by h is written as h𝒮.

### Equivariant embeddings and equivariant models.

A represenation R of a group H is a function that maps each h∈H to a complex matrix R(h) such that $R\left(h_{1}h_{2}\right)=R\left(h_{1}\right)R\left(h_{2}\right)$ for all $h_{1},h_{2}\in H$. In what follows, we say that an embedding $\vec{x}$ is “equivariant” if and only if there is a representation R of $H_{PSCP}$ such that

**Fig. 1.**
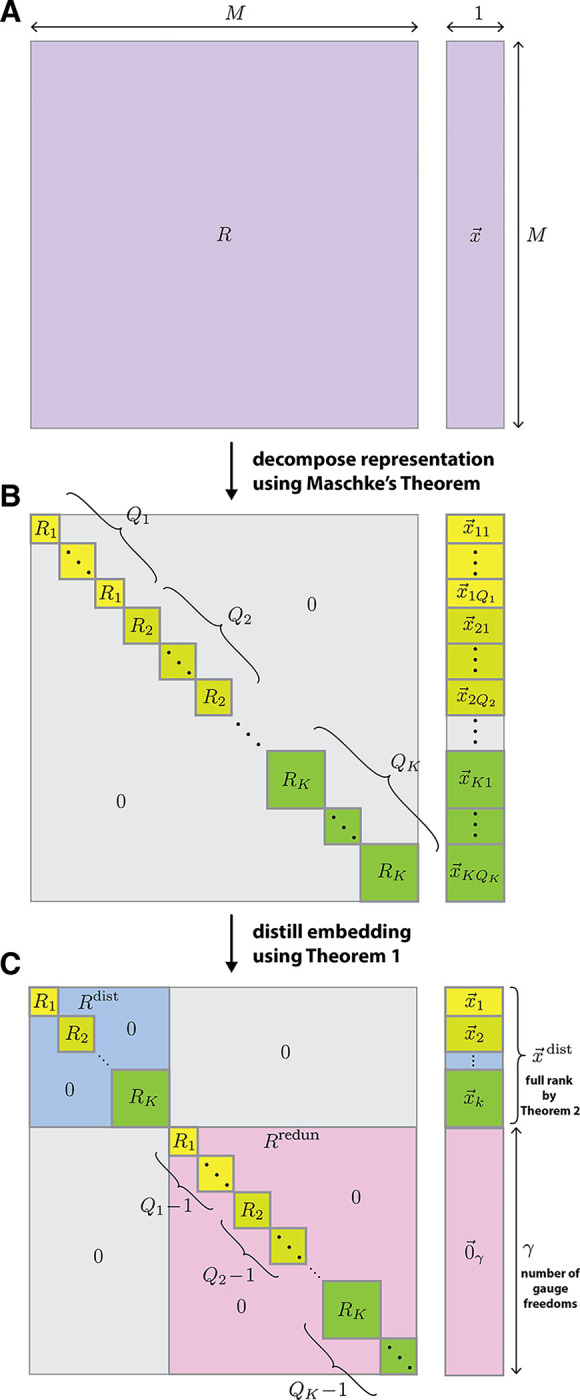
Embedding distillation. (A) Given an M-dimensional embedding $\vec{x}$ that is equivariant under $H_{PSCP}$, let R be the representation of $H_{PSCP}$ that acts on $\vec{x}$. (B) By Maschke’s theorem, R can be decomposed into a direct sum of irreducible representations, $R_{k}\;(k\in \{1,\ldots ,K\})$, each of which occurs with multiplicity $Q_{k}$ (Eq. 8). Similarly, $\vec{x}$ can be decomposed into a direct sum of irreducible embeddings ${\vec{x}}_{kq}\;\left(q\in \left\{1,\ldots ,Q_{k}\right\}\right)$, where each ${\vec{x}}_{kq}$ transforms under $R_{k}$ (Eq. 9). (C) By Theorem 1, an additional similarity transformation can be performed that, for each value of k, zeroes out all but one ${\vec{x}}_{kq}$; the remaining ${\vec{x}}_{kq}$ is denoted by ${\vec{x}}_{k}$ (Eq. 11 and Eq. 12). Consequently, $\vec{x}$ decomposes into a direct sum of a distilled embedding, ${\vec{x}}^{\text{dist}}$, and a zero vector, ${\vec{0}}_{\gamma }$, having dimension γ (Eq. 11). ${\vec{x}}^{\text{dist}}$ is given by the direct sum of all ${\vec{x}}_{k}$ (Eq. 12) and is full rank by Theorem 2. The distilled representation, $R^{\text{dist}}$, describes how ${\vec{x}}^{\text{dist}}$ transforms and contains one copy of each $R_{k}$. The redundant representation, $R^{\text{redun}}$, operates on ${\vec{0}}_{\gamma }$ and encapsulates the $Q_{k}-1$ redundant copies of each $R_{k}.\;\gamma$, the degree of $R^{\text{redun}}$, is equal to the number of gauge freedoms (Eq. 15).

[7] $$\vec{x}(hs)=R(h)\vec{x}(s)$$

for all $h\in H_{PSCP}$ and all s∈𝒮 (Fig. 1A). We also say that a linear model is equivariant if and only if it is defined with an equivariant embedding $\vec{x}$ in Eq. 1. For any equivariant model, the transformation of sequence space by any $h\in H_{PSCP}$ can be compensated for by a corresponding transformation of model parameters. Specifically, the sequence-space transformation $S\to h𝒮,\;h\in H_{PSCP}$, is compensated for by the parameter transformation $\vec{\theta }\to R(h{)}^{-1†}\vec{\theta }$, in the sense that $f\;(s;\vec{\theta })=f\;(hs;\;R(h{)}^{-1†}\vec{\theta })$ for every s∈𝒮 and $\vec{\theta }\in C^{M}$ (see SI Sec. 3.2). Using terminology from representation theory, every R(h) is an M×M matrix where M is called the degree of R (denoted deg R). Similarly $\vec{x}(s)$ is an M-dimensional vector, where m is called the degree of $\vec{x}$ (denoted deg $\vec{x}$).

### Maschke decomposition of equivariant embeddings.

Every group representation is either reducible or irreducible. A representation is irreducible if and only if it has no proper invariant subspace. Maschke’s theorem, a basic result in repreentation theory, says that all representations of finite groups are equivalent (i.e., equal up to a similarity transformation) to a direct sum of irreducible representations. Any representation R of $H_{PSCP}$ can therefore be expressed as

[8] $$R≃\overset{K}{\underset{k=1}{⊕}}Q_{k}R_{k},$$

where ≃ denotes equivalence, each $R_{k}$ is an irreducible repreentation of $H_{PSCP}$, all $R_{k}$ are pairwise inequivalent, and $Q_{k}$ denotes the multiplicity of $R_{k}$ in the direct sum.

In what follows, we say that a sequence embedding is irreducible if and only if it transforms under an irreducible representation of $H_{PSCP}$. One consequence of Eq. 8 is that any embedding $\vec{x}$ that transforms under R can be decomposed as

[9] $$\vec{x}≃\overset{K}{\underset{k=1}{⊕}}\overset{Q_{k}}{\underset{q=1}{⊕}}{\vec{x}}_{kq},$$

where each ${\vec{x}}_{kq}$ is an irreducible embedding that transforms under $R_{k}$. This decomposition is illustrated in Fig. 1B. We assume in what follows that all ${\vec{x}}_{kq}$ are nonzero, but this assumption can be removed without fundamentally changing our results.^‡^

### Distillation of equivariant embeddings.

We now describe how equivariant models are analyzed via the distillation of their embeddings. In SI Sec. 5.1, we prove the following:

#### Theorem 1

*Any two nonzero sequence embeddings that transform under the same irreducible representation of* $H_{PSCP}$ *are equal up to a constant of proportionality*.

Using Theorem 1, then performing additional similarity transformations to remove the constants of proportionality, we obtain,

[10] $$\vec{x}≃\overset{K}{\underset{k=1}{⊕}}Q_{k}{\vec{x}}_{k},$$

where ${\vec{x}}_{k}$ is any one of the ${\vec{x}}_{kq}$, and $Q_{k}$ is the multiplicity of ${\vec{x}}_{k}$ in the direct sum. Additional similarity transformations can then be performed to zero out all except one copy of ${\vec{x}}_{k}$. We therefore find that there is an invertible “distillation matrix” T such that

[11] $$T\vec{x}={\vec{x}}^{\text{dist}}⊕{\vec{0}}_{\gamma },$$

where ${\vec{0}}_{\gamma }$ is a γ-dimensional vector of zeros, and

[12] $${\vec{x}}^{\text{dist}\;}=\overset{K}{\underset{k=1}{⊕}}{\vec{x}}_{k},$$

is the distilled embedding. Similarly, the matrix representation R decomposes as

[13] $$TRT^{-1}={\vec{R}}^{\text{dist}}⊕{\vec{R}}^{\text{redun}}$$

where the distilled representation, $R^{\text{dist}}={⨁}_{k=1}^{K}R_{k}$, contains one copy of each $R_{k}$, and the redundant representation, $R^{\text{redun}}={⨁}_{k=1}^{K}\;\left(Q_{k}-1\right)R_{k}$, contains all of the other copies of each $R_{k}$ that are present in R. These decompositions are illustrated in Fig. 1C.

### Identification of gauge freedoms in equivariant models.

To identify the gauge freedoms of any equivariant model, we use the fact that ${\vec{x}}^{\text{dist}}$ is full rank. This is a consequence of the following Theorem, which is proven in SI Sec. 3.4:

#### Theorem 2

*For each* k∈{1,…,K}, *let* ${\vec{x}}_{k}$ *be a nonzero embedding that transforms under an irreducible representation* $R_{k}$ *of the group* $H_{PSCP}$. *Then the direct sum of all* ${\vec{x}}_{k}$ *is full rank if all* $R_{k}$ *are pairwise inequivalent*.

Because ${\vec{x}}^{\text{dist}}$ is full rank, ${\vec{g}}^{†}\vec{x}(s)=0$ for all s∈𝒮 if and only if

[14] $$\vec{g}=T^{†}[{\vec{0}}_{M-\gamma }⊕{\vec{g}}_{\gamma }],$$

for some γ-dimensional vector ${\vec{g}}_{\gamma }$. The space of gauge transformations, G, is therefore given by the set of vectors having the form in Eq. 14. In particular, the number of gauge freedoms is,

[15] $$\gamma =\deg\vec{x}-\deg{\vec{x}}^{\text{dist}}=\deg R^{\text{redun}}.$$

We thus see that the number of gauge freedoms is equal to the sum of the degrees of all redundant irreducible representations in R.

From Eq. 14, we also see that G is spanned by the last γ column vectors of $T^{†}$. One can therefore compute a basis for G simply by computing T, and computing T only requires keeping track of the similarity transformations needed to express $\vec{x}$ in the distilled form shown in Eq. 11.

### Identification of all equivariant embeddings.

The specific structure of $H_{PSCP}$ allows us to identify all possible inequivalent irreducible equivariant embeddings, ${\vec{x}}_{k}$. Because ${\vec{x}}_{k}$ is irreducible and $H_{PSCP}$ is a product group, ${\vec{x}}_{k}$ can be expressed as

[16] $${\vec{x}}_{k}≃\overset{L}{\underset{l=1}{⊗}}{\vec{x}}_{l}^{k},$$

where each ${\vec{x}}_{l}^{k}$ is an embedding that depends only on the character at position l and that transforms under an irreducible representation of $H_{CP}^{l}$. Moreover, because $H_{CP}^{l}$ is isomorphic to $S_{\alpha }$ and $S_{\alpha }$ supports only two inequivalent embeddings (see SI Sec. 4.3 for proof), there are only two inequivalent choices for each ${\vec{x}}_{l}^{k}$ : the trivial embedding and the simplex embedding. The trivial embedding, denoted ${\vec{x}}^{\text{triv}}$, maps every sequence to a one-dimensional vector and transforms under what is called the “trivial representation” of $S_{\alpha }$. The simplex embedding, denoted ${\vec{x}}_{l}^{\text{sim}}$, maps sequences to the α vertices of an α−1 dimensional simplex and transforms under what is called the “standard representation” of $S_{\alpha }$. One example of the simplex embedding is the tetrahedral embedding of DNA and RNA (20, 22). Note: to lessen the notational burden in what follows, we avoid writing ${\vec{x}}^{\text{triv}}$ within tensor products over positions l, and only show factors that contribute nontrivially to these products.

We now identify all equivariant embeddings $\vec{x}$. Because there are 2 inequivalent choices for each ${\vec{x}}_{l}^{k}\;({\vec{x}}^{\text{triv}}$ or ${\vec{x}}_{l}^{\text{sim}}$), there are $2^{L}$ inequivalent choices for ${\vec{x}}_{k}$, and thus 2LK inequivalent choices for the set ${\left\{{\vec{x}}_{k}\right\}}_{k=1}^{K}$. Letting K in Eq. 12 range from 0 to $2^{L}$, we find that there are ∑K=02L2LK=22L inequivalent choices for ${\vec{x}}^{\text{dist}}$. Every equivariant embedding $\vec{x}$ can therefore be expressed, using one of these $2^{2^{L}}$ inequivalent distilled embeddings ${\vec{x}}^{\text{dist}}$ together with a zero vector ${\vec{0}}_{\gamma }$ and an invertible matrix T. Conversely, choosing any of the $2^{2^{L}}$ inequivalent distilled embeddings ${\vec{x}}^{\text{dist}}$, any non-negative integer γ, and any invertible matrix T of the appropriate size will yield an equivariant embedding $\vec{x}$ via Eq. 11. We therefore find that, modulo the choice of the similarity matrix T and number of gauge freedoms γ, there are $2^{2^{L}}$ distinct choices for $\vec{x}$.

### Analytical analysis of pairwise-interaction models.

We now demonstrate the embedding distillation procedure on the pairwise-interaction model. First we specify the pairwise-interaction embedding, ${\vec{x}}_{\text{pair}}$, as a direct sum of direct products f simpler embeddings:

[17] $${\vec{x}}_{\text{pair}}={\vec{x}}^{\text{triv}}⊕\left\{\underset{l}{⊕}{\vec{x}}_{l}^{\text{ohe}}\right\}⊕\left\{\underset{l<l^{\prime }}{⊕}{\vec{x}}_{l}^{\text{ohe}}⊗{\vec{x}}_{l^{\prime }}^{\text{ohe}}\right\},$$

where ${\vec{x}}_{l}^{\text{ohe}}$ is a position-specific one-hot embedding of dimension α given by

[18] $${\vec{x}}_{l}^{\text{ohe}}(s)=\left[\begin{array}{c} x_{l}^{c_{1}}(s)\\ \vdots \\ x_{l}^{c_{\alpha }}(s) \end{array}\right]$$

for all sequences s, where $c_{1},\ldots ,c_{\alpha }$ denote the elements of 𝒜. The number of model parameters is equal to the dimension of ${\vec{x}}_{\text{pair}}$, which is seen from Eq. 17 to be deg x→pair=1+Lα+L2α2.

The gauge freedoms of pairwise-interaction models arise because ${\vec{x}}_{\text{pair}}$ is not full rank. The reduced rank of ${\vec{x}}_{\text{pair}}$ is a consequence of the fact that ${\vec{x}}_{l}^{\text{ohe}}$ is reducible. To derive a distilled version of ${\vec{x}}_{\text{pair}}$ that is full rank, we reexpress ${\vec{x}}_{l}^{\text{ohe}}$ as a direct sum of irreducible embeddings using

[19] $${\vec{x}}_{l}^{ohe}≃{\vec{x}}^{triv}⊕{\vec{x}}_{l}^{sim};$$

see SI Sec. 2.4 for details. Plugging Eq. 19 into Eq. 17, expanding the direct product, and grouping like terms, we get

[20] $${\vec{x}}_{\text{pair}}≃\left[1+L+(\begin{array}{c} L\\ 2 \end{array})\right]{\vec{x}}^{\text{triv}}⊕\left\{\underset{l}{⊕}L{\vec{x}}_{l}^{\text{sim}}\right\}⊕\left\{\underset{l<l^{\prime }}{⊕}{\vec{x}}_{l}^{\text{sim}}⊗{\vec{x}}_{l^{\prime }}^{\text{sim}}\right\},$$

where the scalar coefficients denote the multiplicity of each term in the direct sum. Because ${\vec{x}}^{\text{triv}},\;{\vec{x}}_{l}^{\text{sim}}$, and ${\vec{x}}_{l}^{\text{sim}}⊗{\vec{x}}_{l}^{\text{sim}}$ are irreducible and pairwise inequivalent, the distillation of ${\vec{x}}_{\text{pair}}$ is seen from Eq. 20 to be

[21] $${\vec{x}}_{\text{pair}}^{\text{dist}}={\vec{x}}^{\text{triv}}⊕\left\{\underset{l}{⊕}{\vec{x}}_{l}^{\text{sim}}\right\}⊕\left\{\underset{l<l^{\prime }}{⊕}{\vec{x}}_{l}^{\text{sim}}⊗{\vec{x}}_{l^{\prime }}^{\text{sim}}\right\}.$$

From this we observe that deg x→pairdist=1+L(α−1)+L2(α−1)2. The number of gauge freedoms then follows from Eq. 15:

[22] γ=L+L22α−1,

which matches the well-known result (3).

### Generalized equivariant one-hot (GEO) models.

For a GO model to be equivariant, it is sufficient for the model to be expressible as a sum of equivariant terms, each term of the form

[23] $$\sum_{c_{1}\in 𝒜}\cdots \sum_{c_{K}\in 𝒜}\theta_{l_{1}l_{2}\ldots l_{K}}^{c_{1}c_{2}\ldots c_{K}}x_{l_{1}l_{2}\ldots l_{K}}^{c_{1}c_{2}\ldots c_{K}}(s),$$

for some term-specific choice of K and term-specific set of positions $\left\{l_{1},\ldots ,l_{K}\right\}$. Observe that GEO models differ from GO models in that, for every set of positions used to define a term, a GEO model sums over all possible characters at all positions in the set, whereas a GO model need not include terms for every possible choice of characters. An example of GO models that are not GEO models are those based on wild-type embeddings, i.e., embeddings that exclude features that involve character-positions pairs that occur in a chosen “wild-type” sequence.

The embeddings of GEO models all have the following form. Let $A_{j}$ denote a set of sequence positions, and let ${\left\{A_{j}\right\}}_{j=1}^{J}$ denote the sets of positions used to construct an GEO model with sequence embedding $\vec{x}$. By analogy to Eq. 17, $\vec{x}$ can then be written as

[24] $$\vec{x}=\overset{J}{\underset{j=1}{⊕}}\underset{l\in A_{j}}{⊗}{\vec{x}}_{l}^{\text{ohe}}.$$

Because each direct product in Eq. 24 yields an embedding of dimension $\alpha^{\left|A_{j}\right|}$, the full dimension of $\vec{x}$ (and thus the number of model parameters) is

[25] $$\deg\vec{x}=\sum_{j=1}^{J}\alpha^{\left|A_{j}\right|}.$$

### Analytical analysis of GEO models.

Now we derive the corresponding distilled embedding. Using Eq. 19 to decompose each ${\vec{x}}_{l}^{\text{ohe}}$ in terms of ${\vec{x}}^{\text{triv}}$ and ${\vec{x}}_{l}^{\text{sim}}$, then expanding each tensor product and grouping the resulting terms, we find that $\vec{x}$ is given by Eq. 10 where

[26] $${\vec{x}}_{k}=\underset{l\in B_{k}}{⊗}{\vec{x}}_{l}^{\text{sim}},$$

where each $B_{k}\;(k\in \{1,\ldots ,K\})$ denotes a subset of positions that occurs among at least one of the $A_{j}$, and $Q_{k}$ denotes the number of $A_{j}$ in which $B_{k}$ occurs.^§^ By inspection we see that each ${\vec{x}}_{k}$ in Eq. 26 has dimension $(\alpha -1{)}^{\left|B_{k}\right|}$. Therefore, the dimension of $\vec{x}$ can alternatively be written as

[27] $$\text{deg}\;\vec{x}=\sum_{k=1}^{K}Q_{k}{\left(\alpha -1\right)}^{\left|B_{k}\right|}.$$

Every ${\vec{x}}_{k}$ is irreducible because every ${\vec{x}}_{l}^{\text{sim}}$ is irreducible. Consequently, the distilled embedding ${\vec{x}}^{\text{dist}}$ is given by Eq. 12 and has dimension

[28] $$\text{deg}\;{\vec{x}}^{\text{dist}}=\sum_{k=1}^{K}{\left(\alpha -1\right)}^{\left|B_{k}\right|}.$$

Using Eq. 15, the number of gauge freedoms of the embedding $\vec{x}$ is thus seen to be

[29] $$\gamma =\sum_{k=1}^{K}\left(Q_{k}-1\right){\left(\alpha -1\right)}^{\left|B_{k}\right|}.$$

This result provides a way to analytically compute the number of gauge freedoms of any GEO model. Table 1 reports the number of gauge freedoms thus computed for a variety of such models. SI Sec. 6 provides expanded descriptions for each model, as well as detailed computations of the results in Table

We note that the only GEO models that have no gauge freedoms are those that have embeddings built from only one tensor product in Eq. 24. To see this, observe from Eq. 29 that γ=0 if and only if none of the $Q_{k}$ are greater than 1. This requires that none of the $B_{k}$ are subsets of two or more $A_{j}$. But the empty set, ∅, is a subset of every $A_{j}$, which means that $Q_{k}=J$ whenever $B_{k}=\emptyset$. Gauge freedoms will therefore be present unless J=1, i.e. the direct sum in Eq. 24 includes only one term.

### Computational analysis of GEO models.

To derive a basis or the space of gauge freedoms, we must choose a specific realization of the irreducible embeddings ${\vec{x}}^{\text{triv}}$ and ${\vec{x}}^{\text{sim}}$. In what follows we choose ${\vec{x}}^{\text{triv}}(s)=[1]$ and

[30] $${\vec{x}}_{l}^{sim}(s)=\left\{\begin{array}{c} \left[\begin{array}{c} x_{l}^{c_{1}}(s)\\ \vdots \\ x_{l}^{c_{\alpha -1}}(s) \end{array}\right]\;\;\text{if}\;s_{l}\neq c_{\alpha },\\ \left[\begin{array}{c} -1\\ \vdots \\ -1 \end{array}\right]\;\;\text{if}\;s_{l}=c_{\alpha }, \end{array}\right.$$

for all sequences s, where $c_{1},\ldots ,c_{\alpha }$ represent an ordering of the characters in 𝒜. With these choices in hand, Eq. 19 can be written as an equality:

[31] $$T^{\left(1\right)}{\vec{x}}^{\text{ohe}}={\vec{x}}^{\text{triv}}⊕{\vec{x}}^{\text{sim}},$$

where $T^{\left(1\right)}$ is an α×α matrix given by

[32] $$T^{\left(1\right)}=\left[\begin{array}{rrrrr} 1 & 1 & \cdots & 1 & 1\\ 1 & 0 & \cdots & 0 & -1\\ 0 & 1 & \cdots & 0 & -1\\ \vdots & \vdots & \ddots & \vdots & \vdots \\ 0 & 0 & \cdots & 1 & -1 \end{array}\right].$$

Using $T^{\left(1\right)}$ one can compute the distillation matrix for any GEO model as the product of three matrices:

[33] $$T=T_{\text{sort}}{\;T}_{\text{thin}}\;T_{\text{decom}}.$$

**Fig. 3.**
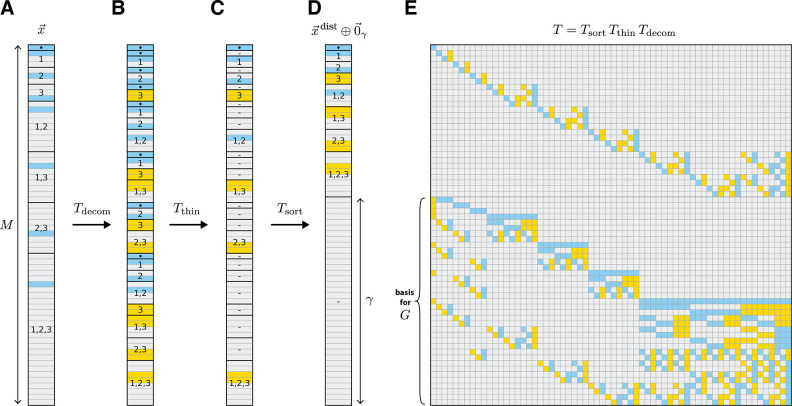
Illustrated distillation computation. (A) Embedding $\vec{x}$ of sequence s=ABC for an all-order interaction model based on the alphabet 𝒜={A,B,C}. Embedding has degree M=64. (B) Result of multiplication by the decomposition matrix, $T_{\text{decom}}$. (C) Result of subsequent multiplication by the thinning matrix $T_{thin}$. (D) Result of subsequent multiplication by the sorting matrix $T_{\text{sort}}$, which yields ${\vec{x}}^{\text{dist}}⊕{\vec{0}}_{\gamma }$ with γ=37 gauge freedoms. In B-D, dots indicate ${\vec{x}}^{\text{triv}}$, dashes indicate zero vectors, and nuumbers indicate ${\vec{x}}_{l}^{\text{sim}}$ or Kronecker products thereof for specified positions l. (E) Distillation matrix T that implements the full distillation procedure in A-D. Last γ rows of T provide a sparse basis for the gauge space, G. In A-E, vector and matrix elements are colored using: blue, +1 ; yellow, −1 ; gray, 0.

The effects of these three matrices are illustrated in Fig. 3. The “decomposition matrix”, $T_{\text{decom}}$, decomposes the one-hot embedding $\vec{x}$ (Fig. 3A) into a direct sum of irreducible embeddings (Fig. 3B). The “thinning matrix”, $T_{\text{thin}}$, then zeros out all except the first copy of each irreducible embedding (Fig. 3C). The “sorting matrix”, $T_{\text{sort}}$, then rearranges the direct sum so that the remaining nonzero embeddings come first (Fig. 3D). SI Sec. 8 provides explicit algorithms for constructing $T_{\text{decom}},\;T_{\text{thin}}$, and $T_{\text{sort}}$, as well as the inverse of each of these three matrices, for a large class of GEO models. Each of these six matrices has only O(L) nonzero elements, and the algorithm for constructing each matrix has O(L) computational complexity. The resulting distillation matrix T, as well as its inverse, are also sparse. Moreover, every nonzero element of T is +1 or −1 (Fig. 3E). Because the last γ columns of $T^{†}$ provide a basis for G, we thus obtain a basis for the gauge space consisting of sparse vectors whose only nonzero elements are +1 and −1.

This result can also be used to efficiently fix the gauge of any GEO. Define the projection matrix

[34] $$P={\left.T^{†}\right|}_{M-\gamma }T^{-1†},$$

where ${|}_{M-\gamma }$ denotes that the last γ columns of a matrix have been set to zero. P projects parameter vectors $\vec{\theta }$ onto the spaced spanned by the first M−γ columns of $T^{†}$. Moreover, by expanding P as

[35] $$P={\left.T_{\text{decom}}^{†}\;T_{\text{thin}}^{†}{\;T}_{\text{sort}}^{†}\;I\right|}_{M-\gamma }T_{\text{sort}}^{-1†}T_{\text{thin}}^{-1†}T_{\text{decom}}^{-1†},$$

and applying each matrix factor to $\vec{\theta }$ individually, this projection can be performed in O(L) time. Projection by P therefore provides and efficient way to remove gauge freedoms by projecting model parameters into a linear gauge. We note, however, that the resulting linear gauge is not one of the parametric gauges discussed in our companion paper (31).

### Interpretability of pairwise-interaction models.

**Fig. 4.**
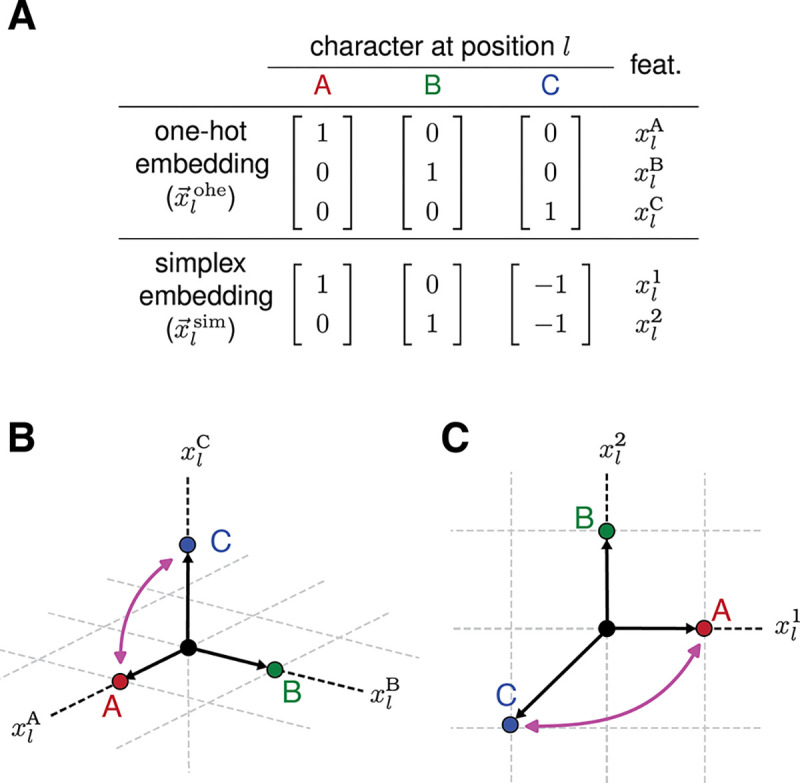
Transformation behavior of two single-position embeddings. (A) Two single-position embeddings, ${\vec{x}}_{l}^{\text{ohe}}$ and ${\vec{x}}_{l}^{\text{sim}}$, for the three-character alphabet A={A,B,C}. The specific features corresponding to each element of ${\vec{x}}_{l}^{\text{ohe}}$ and ${\vec{x}}_{l}^{\text{sim}}$ are also shown. (B) The three-dimensional one-hot embedding, ${\vec{x}}_{l}^{\text{ohe}}(c)$, for each c∈𝒜. (C) The two-dimensional simplex embedding, ${\vec{x}}_{l}^{\text{sim}}(c)$, for each c∈𝒜. Pink arrows indicate the transformation of each embedding vector induced by permuting characters A and C at position l.

The ability to interpret the parameters of equivariant models as quantifying allelic effects is closely related to how those parameters transform under $H_{PSCP}$. To illustrate this point, we consider two equivariant models: a pairwise-interaction GEO model with embedding ${\vec{x}}_{\text{pair}}$, and the corresponding distilled model with embedding ${\vec{x}}_{\text{pair}}^{\text{dist}}$, both operating on sequences built from a three-character alphabet, 𝒜={A,B,C}. The two embeddings encode the same set of interactions but do so in different ways: ${\vec{x}}_{\text{pair}}$ is built from the single-position one-hot encodings ${\vec{x}}_{l}^{\text{ohe}}$, whereas ${\vec{x}}_{\text{pair}}^{\text{dist}}$ is built from the single-position simplex encodings ${\vec{x}}_{l}^{\text{sim}}$ (Fig. 4A). And as we show above, the GEO model has gauge freedoms whereas the distilled model does not.

We now focus on how the features and parameters of these two models are affected by the transformation $h\in H_{PSCP}$ that exchanges the characters A and C at all positions l. For the GEO model, h induces a permutation of embedding coordinates (Fig. 4B) and thus of model parameters. Consequently, h preserves the set of values taken by the GEO parameters; it simply permutes which parameters have which values. This mirrors the action of h on the alleles that drive model predictions: h permutes sequences and thus the one-position and two-position alleles they contain, but does not alter the full set of alleles present among the full set of sequences. And in fact we see that individual parameter values track their corresponding alleles: $\theta_{l}^{A}$ and $\theta_{l}^{C}$ switch values, $\theta_{ll^{\prime }}^{AA}$ and $\theta_{ll^{\prime }}^{CC}$ switch values, etc.. The transformation behavior of the GEO model is therefore consistent with individual parameters quantifying the effects of individual alleles.

For the distilled model, however, h induces a non-permutation transformation of embedding coordinates (Fig. 4C) and thus of model parameters. Using the embedding shown in Fig. 4A, one finds that the value of $\theta_{l}^{1}$ transforms to $-\theta_{l}^{1}+\theta_{l}^{2}$, the value of $\theta_{ll^{\prime }}^{11}$ transforms to $\theta_{ll^{\prime }}^{11}-\theta_{ll^{\prime }}^{12}-\theta_{ll^{\prime }}^{21}+\theta_{ll^{\prime }}^{22}$, etc.. The transformation h therefore changes the full set of values taken by the distilled model parameters. Consequently, the individual parameters of this model cannot be interpreted as quantifying the effects of individual alleles.

### Nontrivial equivariant allelic models have gauge freedoms.

To clarify the connection between the interpretation and transformation behavior of model parameters, we now formalize the notion of an allele, and allelic effect, and related concepts. We define an allele a to be a pattern of characters that is either present or absent in every sequence. The corresponding allelic set ${𝒮}_{a}$ is defined to be the set of sequences that have allele a, and the corresponding allelic feature $x_{a}$ is defined be the indicator function for membership in ${𝒮}_{a}$. An allelic model is defined to be a linear sequence-function relationship in which every feature is an allelic feature. The effect of allele a is defined, in the context of a specific allelic model, to be the parameter $\theta_{a}$ that multiplies the allelic feature $x_{a}$.

Requiring an allelic model to be equivariant puts strong constraints on which alleles it can describe, and on how the corresponding allelic features and allelic effects transform. Given a specific allele a, the action of $H_{PSCP}$ on a generates a set of alleles 𝒪, which we call an allelic orbit. If the allelic model is equivariant, the allelic sets ${𝒮}_{a^{\prime }}$ corresponding to all $a^{\prime }\in 𝒪$ will tile sequence space without overlaps. This requirement greatly constraints the set of possible alleles such a model can describe. Moreover, the model must include one feature $x_{a^{\prime }}$ for every allele $a^{\prime }\in 𝒪$. These features will then transform among themselves according to a permutation representation. See SI Sec. 9 for details.

An equivariant allelic model must therefore contain features that can be partitioned into a set of complete allelic orbits. The features of the model will then transform under a direct sum of permutation representations, one for each allelic orbit. Because every permutation representation contains the trivial representation in its Maschke decomposition, the allelic model will have at least as many gauge freedoms as the number of allelic orbits minus one. Perhaps more intuitively, the sum of all allelic features corresponding to each orbit is equal to one for all sequences. Therefore, each orbit’s features are sufficient to represent a constant function on sequence space. Including features from multiple orbits therefore overparameterizes the model and introduces gauge freedoms. We emphasize, however, that additional gauge freedoms can be present as well, so this result only provides a lower bound on γ.

It is readily seen that all GEO models are allelic models. In a GEO model, each allelic orbit corresponds to a position set $A_{j}$ in Eq. 24, and the number of allelic orbits is given by J. Our lower-bound on the number of gauge freedoms recapitulates the finding above that only GEO models with J=1 have no gauge freedoms. We also show in SI Sec. 9 that, given a model defined by a direct sum of direct products of single-position embeddings, the corresponding GEO model has the smallest number of gauge freedoms possible.

We therefore see that there is an incompatibility between two distinct notions of parameter interpretability. In all except a limited class of models, the ability to interpret parameters as quantifying allelic effects is incompatible with the ability to interpret parameter values in the absence of gauge-fixing constraints. The only exceptions to this rule are single-orbit allelic models, but these models are trivial in the following siense: ^¶^ each sequence has only one allele, the effect of which is the model’s prediction for the sequence. In a single-orbit allelic model, each sequence has only one allele–and thus one feature and one parameter–that contributes to its activity. The parameters are therefore essentially just a catalog of allelic effects. By contrast, the reason researchers quantitatively model sequence-function relationships in the first place is to deconvolve the influence of multiple co-occurring alleles. We onclude that, among nontrivial equivariant models (i.e., modls that support co-occurring alleles), the ability to interpret model parameters as quantifying allelic effects requires that the model have gauge freedoms.

## Discussion

Motivated by the connection between gauge freedoms and symmetries in physics, we investigated the relationship between gauge freedoms and symmetries in quantitative models of sequence-function relationships. We found that, for models that are equivariant under the group of position-specific character permutations (denoted $H_{PSCP}$), gauge freedoms arise due to model parameters transforming according to redundant irreducible matrix representations of $H_{PSCP}$. From a practical standpoint, this result facilitates the analytic calculation of the dimension of the space of gauge freedoms in a large class of commonly used models, as well as the efficient computation of a sparse basis for this space. From a conceptual standpoint, the results link the gauge freedoms of models of sequence-function relationships to the transformation behavior of these models under a specific symmetry group of sequence space.

We also investigated the link between parameter transformation behavior and parameter interpretability. In doing so, we identified a tension between two different notions of parameter interpretability: in all nontrivial equivariant models, the ability to interpret the values of model parameters in the absence of gauge-fixing constraints is incompatible with the ability to interpret parameters as quantifying allelic effects. Consequently, models that do have gauge freedoms (including nontrivial additive models, pairwise-interaction models, etc.) have important advantages over equally expressive models that do not have gauge freedoms.

We now return to the analogy with theoretical physics. In classical field theories like E&M, there are specific symmetries that are well-established by experiment and that any mathematical formulation of the theory must be consistent with. This does not, however, mean that the equations of the theory must transform in a simple way under those symmetries. Mathematically formulating physical theories so that the equations themselves manifestly respect the symmetries of the theory generally requires over-parameterizing the equations, thereby introducing gauge freedoms. Physicists often find it worthwhile to do this, as having fundamental symmetries be reflected in one’s equations can greatly facilitate the interpretation and application of those equations. Solving such equations, however, requires fixing the gauge—introducing additional constraints that make the solution of the equations unique.

Unlike in physics, there is no experimentally established requirement that models of sequence-function relationships be equivariant under symmetries of sequence space. The specific mathematical form one uses for such models is subjective, and different models are commonly used in different contexts. Citing the ambiguities caused by gauge freedoms, some have argued for restricting one’s choice of model to those that have no gauge freedoms. Nevertheless, models that have gauge freedoms remain dominant in the literature. We suggest that a major reason for this may be that researchers prefer to use models that both (a) reflect symmetries of sequence space and (b) have parameters that can be interpreted as allelic effects. As we showed, these criteria require the use of over-parameterized models. And in this way, the origin of gauge freedoms in models of sequence-function relationships does mirror the origin of gauge freedoms in physical theories.

There is still much to understand about the relationship between models of sequence-function relationships, the symmetries of these models, and how these modes can be biological interpreted. This paper and its companion (31) have only addressed gauge freedoms and symmetries in linear models of sequence-function relationships. Some work has explored the gauge freedoms and symmetries of nonlinear models of sequence-function relationships (33, 34), but important questions remain. The sloppy modes (35, 36) present in sequencefunction relationships are also important to understand but, to our knowledge, these have yet to be systematically studied. Addressing these problems is becoming increasingly urgent, not just because of the rapidly expanding use of quantitative models of sequence-function relationships, but also because of the emerging use of surrogate models for interpreting sequence-function relationships described by genomic deep neural networks (37).

## Materials and Methods

See Supplemental Information for full derivations of the mathematical results. Python code implementing the embedding distillation algorithm described the section “Computational analysis of GEO models”, as well as used for generating Fig. 3, is available at https://github.com/jbkinney/23_posfai.

## Supplementary Material

Supplement 1

## Figures and Tables

**Fig. 2.**
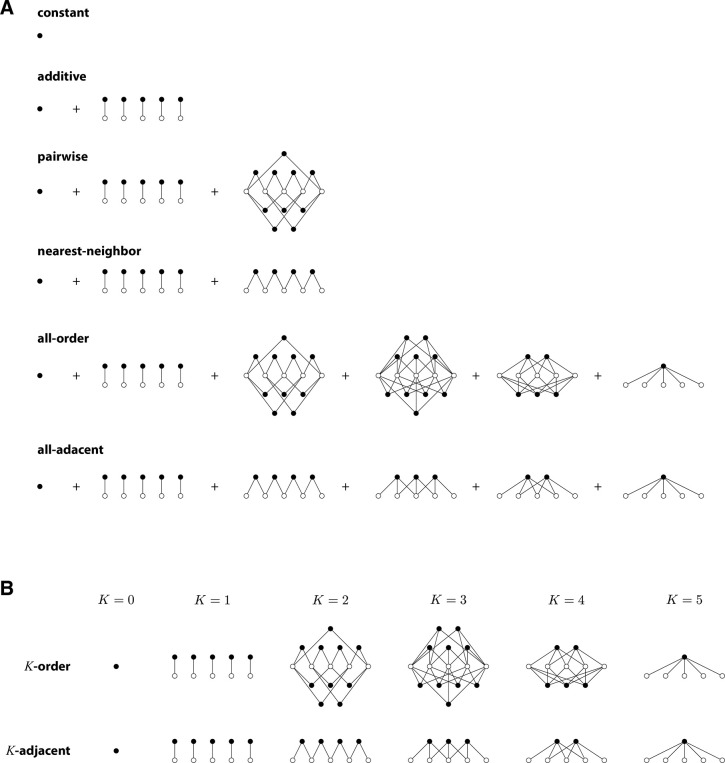
Structure of GEO models. (A,B) Models analyzed in Table 1, illustrated for L=5. Open circles represent sequence positions. Closed circles represent sets of parameters that are closed under the action of $H_{PSCP}$, as in Eq. 23. Edges indicate position indices shared by all the parameters within each closed set. (A) Structure of specific models of interest. (B) Structure of K-order models and K-adjacent models for various interaction orders K.

**Table 1.** Number of parameters and gauge freedoms of various GEO models. Columns show model type, the orders of interaction included in each model, the number of parameters of each model, and the number of gauge freedoms of each model. See SI Sec. 6 for derivations of these results. GEO, generalized equivariant one-hot.

model type	interaction orders	no. parameters (M)	no. gauge freedoms (γ)

constant	0	1	0
additive	0, 1	1+Lα	L
pairwise	0, 1, 2	1+Lα+L2α2	L+L2(2α−1)
nearest-neighbor	0, 1, 2	$1+L\alpha +(L-1)\alpha^{2}$	L+(L−1)(2α−1)
all-order	0,1,…,L	$(\alpha +1{)}^{L}$	$(\alpha +1{)}^{L}-\alpha^{L}$
all-adjacent	0,1,…,L	$1+\frac{\alpha }{(\alpha -1{)}^{2}}\left[\alpha^{L+1}-(L+1)\alpha +L\right]$	$1+\frac{\alpha }{(\alpha -1{)}^{2}}\left[2\alpha^{L}-\alpha^{L-1}-(L+1)\alpha +L\right]$

K-order	K	LKαK	LKαK−∑k=0KLk(α−1)k
hierarchical K-order	0,1,…,K	∑k=0KLkαk	∑n=0KLkαk−(α−1)k

K-adjacent^†^	K	$(L-K+1)\alpha^{K}$	$(L-K)\alpha^{K-1}$
hierarchical K-adjacent^†^	0,1,…,K	$1+\sum_{k=1}^{K}\;(L-k+1)\alpha^{k}$	$(L-K)\alpha^{K-1}+1+\sum_{k=1}^{K-1}\;(L-k+1)\alpha^{k}$

† Assumes *K* ≥ 1.

## Footnotes

* Results in quantum physics, such as the Aharanov-Bohm effect (28, 29), suggest a reality to the four-potential beyond what can be inferred solely from classical E&M, though there are arguments against this interpretation (30).

† Here and in what follows, K=0 corresponds to feature $x_{0}$ and parameter $\theta_{0}$.

‡ See SI Sec. 5.2 for a statement of our main results when this assumption is removed.

§ Formally, ${\left\{B_{k}\right\}}_{k=1}^{K}={⋃}_{j=1}^{J}\;P\left(A_{j}\right)$ where P(⋅) denotes the powerset, $Q_{k}=\sum_{j=1}^{J}\;1_{P\left(A_{j}\right)}\left(B_{k}\right)$ where $1_{P(A)}(\cdot )$ is the indicator function for membership in P(A).

¶ This is the same sense in which the “trivial gauge” described in (31) is trivial.

## References

[1] Kinney JB, McCandlish DM, Massively parallel assays and quantitative sequence-function relationships. Annu. Rev. Genomics Hum. Genet. 20, 99–127 (2019) Wrote. 31091417 10.1146/annurev-genom-083118-014845

[2] JB Kinney, Tkacik G, Callan CG, Precise physical models of protein-DNA interaction from high-throughput data. Proc. Natl. Acad. Sci. 104, 501–506 (2007) Wrote. 17197415 10.1073/pnas.0609908104 PMC1766414

[3] Weigt M, White RA, Szurmant H, Hoch JA, Hwa T, Identification of direct residue contacts in protein-protein interaction by message passing. Proc. Natl. Acad. Sci. 106, 67–72 (2009). 19116270 10.1073/pnas.0805923106 PMC2629192

[4] Marks DS, , Protein 3D Structure Computed from Evolutionary Sequence Variation. PLoS ONE 6, e28766 (2011) 22163331 10.1371/journal.pone.0028766 PMC3233603

[5] Ekeberg M, ovkvist C L, Lan Y, Weigt M, Aurell E, Improved contact prediction in proteins: Using pseudolikelihoods to infer Potts models. Phys. Rev. E 87, 012707 (2013). 10.1103/PhysRevE.87.012707 23410359

[6] Ekeberg M, Hartonen T, Aurell E, Fast pseudolikelihood maximization for direct-coupling analysis of protein structure from many homologous amino-acid sequences. J. Comput. Phys. 276, 341–356 (2014)

[7] Stein RR, Marks DS, Sander C, Inferring Pairwise Interactions from Biological Data Using Maximum-Entropy Probability Models. PLoS Comput. Biol. 11, e1004182 (2015). 26225866 10.1371/journal.pcbi.1004182 PMC4520494

[8] Barton JP, Leonardis ED, Coucke A, Cocco S, ACE: adaptive cluster expansion for maximum entropy graphical model inference. Bioinformatics 32, 3089–3097 (2016). 27329863 10.1093/bioinformatics/btw328

[9] Haldane A, Flynn WF, He P, Levy RM, Coevolutionary Landscape of Kinase Family Proteins: Sequence Probabilities and Functional Motifs. Biophys. J. 114, 21–31 (2018). 29320688 10.1016/j.bpj.2017.10.028 PMC5773752

[10] Cocco S, Feinauer C, Figliuzzi M, Monasson R, Weigt M, Inverse statistical physics of protein sequences: a key issues review. Reports on Prog. Phys. 81, 032601 (2018). 10.1088/1361-6633/aa9965 29120346

[11] Haldane A, Levy RM, Influence of multiple-sequence-alignment depth on Potts statistical models of protein covariation. Phys. Rev. E 99, 032405 (2019). 30999494 10.1103/PhysRevE.99.032405 PMC6508952

[12] Rube HT, , Probing molecular specificity with deep sequencing and biophysically interpretable machine learning. bioRxiv p. 2021.06.30.450414 (2021).

[13] Zamuner S, Rios PDL, Interpretable Neural Networks based classifiers for categorical inputs arXiv (2021)

[14] Feinauer C, Meynard-Piganeau B, Lucibello C, Interpretable pairwise distillations for generative protein sequence models. PLoS Comput. Biol. 18, e1010219 (2022). 35737722 10.1371/journal.pcbi.1010219 PMC9258900

[15] Gerardos A, Dietler N, Bitbol AF, Correlations from structure and phylogeny combine constructively in the inference of protein partners from sequences. PLoS Comput. Biol. 18, e1010147 (2022). 35576238 10.1371/journal.pcbi.1010147 PMC9135348

[16] Hsu C, Nisonoff H, Fannjiang C, Listgarten J, Learning protein fitness models from evolutionary and assay-labeled data. Nat. Biotechnol. 40, 1114–1122 (2022). 35039677 10.1038/s41587-021-01146-5

[17] Feinauer C, Borgonovo E, Mean Dimension of Generative Models for Protein Sequences. bioRxiv p. 2022.12.12.520028 (2022)

[18] Rube HT, , Prediction of protein-ligand binding affinity from sequencing data with interpretable machine learning. Nat. Biotechnol. 40, 1520–1527 (2022). 35606422 10.1038/s41587-022-01307-0 PMC9546773

[19] Weinberger ED, Fourier and taylor series on fitness landscapes. Biol. cybernetics 65,321–330 (1991).

[20] Zhang CT, Zhang R, Analysis of distribution of bases in the coding sequences by a diagrammatic technique. Nucleic acids research 19, 6313–7 (1991). 1956790 10.1093/nar/19.22.6313 PMC329145

[21] Stadler PF, Spectral landscape theory in Evolutionary Dynamics: Exploring the Interplay of Selection, Accident, Neutrality and Function, eds. Crutchfield J, Schuster P. (Oxford Univ Press, Oxford), pp. 231–271 (2003).

[22] Stormo GD, Maximally efficient modeling of DNA sequence motifs at all levels of complexity Genetics 187, 1219 – 1224 (2011-04) 21300846 10.1534/genetics.110.126052 PMC3070529

[23] Poelwijk FJ, Krishna V, Ranganathan R, The Context-Dependence of Mutations: A Linkage of Formalisms. PLOS Comput. Biol. 12, e1004771 (2016). 27337695 10.1371/journal.pcbi.1004771 PMC4919011

[24] Brookes DH, Aghazadeh A, Listgarten J, On the sparsity of fitness functions and implications for learning. Proc. Natl. Acad. Sci. 119, e2109649118 (2022). 34937698 10.1073/pnas.2109649118 PMC8740588

[25] Tareen A, , MAVE-NN: learning genotype-phenotype maps from multiplex assays of variant effect. Genome Biol. 23, 98 (2022). 35428271 10.1186/s13059-022-02661-7 PMC9011994

[26] Jackson JD, Okun LB, Historical roots of gauge invariance. Rev. Mod. Phys. 73, 663–680 (2001)

[27] Jackson JD, Classical electrodynamics. (John Wiley & Sons), (1998).

[28] Aharonov Y, Bohm D, Significance of electromagnetic potentials in the quantum theory. Phys review 115, 485 (1959)

[29] Peshkin M, Tonomura A, The Aharonov-Bohm Effect. (Springer Verlag), (2005).

[30] Vaidman L, Role of potentials in the aharonov-bohm effect. Phys. Rev. A 86, 040101 (2012),

[31] Posfai A, Zhou J, McCandlish DM, Kinney JB, Gauge fixing for sequence-function relationships In prep. (2024). 10.1371/journal.pcbi.1012818 PMC11957564 40111986

[32] Sagan BE, The Symmetric Group: Representations, Combinatorial Algorithms, and Symmetric Functions, Graduate Texts in Mathematics. (Springer), 2 edition, (2001) Read in early 2022.

[33] Kinney JB, Atwal GS, Parametric Inference in the Large Data Limit Using Maximally Informative Models. Neural computation 26, 637–653 (2014-04) Wrote. 24479782 10.1162/NECO_a_00568

[34] Atwal GS, Kinney JB, Learning Quantitative Sequence–Function Relationships from Massively Parallel Experiments. J. Stat. Phys. 162, 1203–1243 (2016) Wrote.

[35] Machta BB, Chachra R, Transtrum MK, Sethna JP, Parameter space compression underlies emergent theories and predictive models. Science 342, 604 – 607 (2013). 24179222 10.1126/science.1238723

[36] Transtrum MK, , Perspective: Sloppiness and emergent theories in physics, biology, and beyond. The J. Chem. Phys. 143, 010901 – 14 (2015). 26156455 10.1063/1.4923066

[37] Seitz E, McCandlish DM, Kinney JB, Koo PK, Interpreting cis-regulatory mechanisms from genomic deep neural networks using surrogate models. bioRxiv (2023). 10.1038/s42256-024-00851-5 PMC11823438 39950082

