# Symmetry, gauge freedoms, and the interpretability of sequence-function relationships

**DOI:** 10.1101/2024.05.12.593774

**Published:** 2024-06-24

**Authors:** Anna Posfai, David M. McCandlish, Justin B. Kinney

**Affiliations:** 1 Simons Center for Quantitative Biology, Cold Spring Harbor Laboratory, Cold Spring Harbor, NY, 11724

**Keywords:** sequence-function relationships, gauge freedoms, sequence space, permutation symmetry, representation theory

## Abstract

Quantitative models that describe how biological sequences encode functional activities are ubiquitous in modern biology. One important aspect of these models is that they commonly exhibit gauge freedoms, i.e., directions in parameter space that do not affect model predictions. In physics, gauge freedoms arise when physical theories are formulated in ways that respect fundamental symmetries. However, the connections that gauge freedoms in models of sequence-function relationships have to the symmetries of sequence space have yet to be systematically studied. Here we study the gauge freedoms of models that respect a specific symmetry of sequence space: the group of position-specific character permutations. We find that gauge freedoms arise when model parameters transform under redundant irreducible matrix representations of this group. Based on this finding, we describe an “embedding distillation” procedure that enables analytic calculation of the number of independent gauge freedoms, as well as efficient computation of a sparse basis for the space of gauge freedoms. We also study how parameter transformation behavior affects parameter interpretability. We find that in many (and possibly all) nontrivial models, the ability to interpret individual model parameters as quantifying intrinsic allelic effects requires that gauge freedoms be present. This finding establishes an incompatibility between two distinct notions of parameter interpretability. Our work thus advances the understanding of symmetries, gauge freedoms, and parameter interpretability in sequence-function relationships.

## Introduction

Understanding the quantitative nature of sequence-function relationships is a major goal of modern biology (1). To study a sequence-function relationship of interest, researchers often propose a mathematical model, fit the parameters of the model to data, then biologically interpret the resulting parameter values. This interpretation step is complicated, however, by gauge freedoms—directions in parameter space along which model parameters can be changed without altering model predictions. If any gauge freedoms are present in a model, the values of individual model parameters cannot be meaningfully interpreted in the absence of additional constraints.

Researchers performing quantitative studies of sequence-function relationships routinely encounter gauge freedoms in their models. In practice, one of two methods is used to overcome the difficulties that gauge freedoms present. One method, called “gauge fixing”, removes gauge freedoms by introducing additional constraints on model parameters (2–18). Another method limits the mathematical models that one uses to models that do not have any gauge freedoms (19–24). But despite being frequently encountered in the course of research, the gauge freedoms present in models of sequence-function relationships have received only limited attention (e.g., 3, 5–7, 12, 25). In particular, the mathematical properties of these gauge freedoms have yet to be systematically studied.

In physics, by contrast, gauge freedoms are a topic of fundamental importance (26). Gauge freedoms are well-known to arise when a physical theory is expressed in a form that manifestly respects fundamental symmetries. For example, the classical theory of electricity and magnetism (E&M) is invariant to Lorentz transformations, i.e., relativistic changes in an observer’s velocity (27). Lorentz invariance is obscured, however, when the equations of E&M are expressed directly in terms of electric and magnetic fields. To express these equations in a form that is manifestly Lorentz invariant, one must instead formulate them in terms of an electromagnetic four-potential. Doing this introduces gauge freedoms because the four-potential, unlike electric and magnetic fields, is neither directly measurable nor uniquely determined by the configuration of a physical system.* Nevertheless, working with the four-potential simplifies the equations of E&M and can aid in both their solution and their physical interpretation.

Motivated by the connection between gauge freedoms and symmetries in physics, we asked whether gauge freedoms in models of sequence-function relationships have a connection to the symmetries of sequence space. Here we study the gauge freedoms of linear models that are equivariant under a specific symmetry group of sequence space—the group of position-specific character permutations. These models include many of the most commonly used models, such as models with pairwise and/or higher-order interactions. Using techniques from the theory of matrix representations, we find that the gauge freedoms of these models arise when the transformations of model parameters that compensate for these symmetry transformations are described by redundant irreducible matrix representations. Based on this finding, we introduce an “embedding distillation” procedure that facilitates analysis of the vector space formed by the gauge freedoms of a model.

We also study the connection between parameter interpretability and model transformation behavior. We find that in many (and possibly all) nontrivial models, the ability to interpret model parameters as quantifying the intrinsic effects of alleles requires that the model have gauge freedoms. This finding shows that models having gauge freedoms can have important advantages over models having no gauge freedoms. A companion paper (31) reports specific gauge-fixing strategies that can be applied to many commonly used models that do have gauge freedoms.

## Background

We now establish definitions and notation used in Results. We also review basic results regarding gauge freedoms in mathematical models of sequence-function relationships. Our companion paper (31) provides an expanded discussion of these results together with corresponding proofs.

### Sequence-function relationships.

Let A denote an alphabet comprising α distinct characters, and let S denote the set of $\alpha^{L}$ sequences of length L built from these characters. A real-valued model of a sequence-function relationship, $f(s;\vec{\theta })$, is defined to be a function that maps each sequence s∈S to a real number. The vector $\vec{\theta }$ denotes the parameters of the model and is assumed to comprise M real numbers. Complex-valued models of sequence-function relationships are defined analogously.

#### Linear models.

Linear models of sequence-function relationships are linear in $\vec{\theta }$ and thus have the form

[1] $$f(s;\vec{\theta })={\vec{\theta }}^{⊤}\vec{x}(s)=\sum_{i=1}^{M}\theta_{i}x_{i}(s),$$

for all s∈S. Here, $\vec{x}(\cdot )$ is an M-dimensional vector of sequence features, and each feature $x_{i}(\cdot )$ is a function that maps S to ℝ. We refer to the space ${\mathbb{R}}^{M}$ in which $\vec{x}$ and $\vec{\theta }$ live as feature space.^†^

An example of a linear model is the pairwise one-hot model, which has the form

[2] $$f_{\text{pair}}^{\text{ohe}}=\theta_{0}x_{0}+\sum_{l}\sum_{c}\theta_{l}^{c}x_{l}^{c}+\sum_{l<l^{\prime }}\sum_{c,c^{\prime }}\theta_{ll^{\prime }}^{cc^{\prime }}x_{ll^{\prime }}^{cc^{\prime }}.$$

Here, l, $l^{\prime }\in \{1,\ldots ,L\}$ index the positions within each sequence s, c, $c^{\prime }\in A$ index the possible characters at these positions, and the sequence arguments of both the model and features are kept implicit. Pairwise one-hot models comprise three types of features: the constant feature, $x_{0}(s)$, which equals one for every sequence s; additive one-hot features, $x_{l}^{c}(s)$, which equal one if $s_{l}=c$ and equal zero otherwise (where $s_{l}$ denotes the character at position l in sequence s); and pairwise one-hot features, $x_{ll^{\prime }}^{cc^{\prime }}(s)$, which equal one if both $s_{l}=c$ and $s_{l^{\prime }}=c^{\prime }$, and which equal zero otherwise.

#### Gauge freedoms.

Gauge freedoms are transformations of model parameters that do not affect model predictions. Formally, a gauge freedom is any vector $\vec{g}\in {\mathbb{R}}^{M}$ that satisfies

[3] $$f(s;\vec{\theta })=f(s;\vec{\theta }+\vec{g})\;\text{for all}\;s\in S\text{.}$$

For linear sequence-function relationships the set of gauge freedoms, denoted by G, forms a vector space in ${\mathbb{R}}^{M}$. It is readily shown that G is the orthogonal complement of the space spanned by sequence embeddings (31). In what follows, we use γ to represent the dimension of G, i.e., the number of (independent) gauge freedoms.

Gauge freedoms arise from linear dependencies among sequence features. By inspection we see that $f_{\text{pair}}^{\text{ohe}}$ has

[4] $$M_{\text{pair}}^{\text{ohe}}=1+\alpha L+\left(\begin{array}{c} L\\ 2 \end{array}\right)\alpha^{2}$$

parameters. However, the space spanned by the corresponding embedding, ${\vec{x}}_{\text{pair}}^{\text{ohe}}$, has only $1+(\alpha -1)L+\left(\begin{array}{c} L\\ 2 \end{array}\right){\left(\alpha -1\right)}^{2}$ dimensions. This difference reflects the presence of $L+\left(\begin{array}{c} L\\ 2 \end{array}\right)(2\alpha -1)$ constraints on the features: $x_{0}=\sum_{c^{\prime }}x_{l}^{c^{\prime }}$ for all positions l (1 constraint per position), and both $x_{l}^{c}=\sum_{c^{\prime }}x_{ll^{\prime }}^{cc^{\prime }}$ and $x_{l^{\prime }}^{c}=\sum_{c^{\prime }}x_{ll^{\prime }}^{c^{\prime }c}$ for all pairs of positions $l<l^{\prime }(2\alpha -1$ independent constraints per pair of positions). The model $f_{\text{pair}}^{\text{ohe}}$ therefore has

[5] $$\gamma_{\text{pair}}^{\text{ohe}}=L+\left(\begin{array}{c} L\\ 2 \end{array}\right)(2\alpha -1)$$

gauge freedoms. See our companion paper (31) for details, as well as (3, 5, 7, 10) for earlier treatments of gauge freedoms in the pairwise one-hot model.

#### Fixing the gauge.

Fixing the gauge is the process of removing gauge freedoms by restricting $\vec{\theta }$ to a subset Θ of parameter space called “the gauge”. Linear gauges are choices of Θ that are vector spaces. One useful property of linear gauges is that gauge-fixing can be accomplished through linear projection. Specifically, for any linear gauge Θ, there exists a projection matrix P that projects each parameter vector $\vec{\theta }\in {\mathbb{R}}^{M}$ onto an equivalent parameter vector ${\vec{\theta }}_{\text{fixed}}\in \text{Θ}$ via ${\vec{\theta }}_{\text{fixed}}=P\vec{\theta }$. Our companion paper describes a parametric family of linear gauges (including an explicit formula for the corresponding projection matrices) that includes as special cases many of the most commonly used gauges in the literature (31).

## Results

We being this section by defining a specific symmetry group of sequence space—the group of position-specific character permutations—as well as the notion of model equivariance under this group. We then illustrate, for two example models, how model transformation behavior under this symmetry group impacts gauge freedoms and parameter interpretability. Next we formally investigate this relationship using methods from the theory of group representations (32). In doing so, we establish an “embedding distillation” procedure that, for any equivariant model, enables analytic calculation of the number of gauge freedoms and efficient computation of a sparse basis for the space of gauge freedoms. We conclude by revisiting the issue of parameter interpretability in light of these results.

### Position-specific character permutations.

Different transformations of sequence space impact models of sequence-function relationships in different ways. Here we focus on a specific kind of transformation: position-specific character permutations. These transformations of sequence space form a mathematical group, which we denote by $H_{\text{PSCP}}$. The action of a transformation $h\in H_{\text{PSCP}}$ on a sequence s∈S is written hs. $H_{\text{PSCP}}$ is a symmetry group of sequence space in that transformations in $H_{\text{PSCP}}$ preserve the Hamming distances between sequences. There are other symmetry groups of sequence space as well, but we ultimately find that these symmetry groups do not have the same connections to gauge freedoms that $H_{\text{PSCP}}$ does (discussed below and in SI Sec. 7).

### Equivariance.

We also focus on linear models of sequence-function relationships for which $H_{\text{PSCP}}$ induces linear transformations of the embeddings and parameters. These linear transformations are called “representations” (32). In general, a representation R of a group H is a function that maps each h∈H to a matrix R(h) in a way that preserves the multiplicative structure of H, i.e., $R\left(h_{1}h_{2}\right)=R\left(h_{1}\right)R\left(h_{2}\right)$ for any two group elements $h_{1},h_{2}\in H$. The degree of the representation R (denoted deg R) is the dimension of the space on which R acts.

In what follows, we say that an embedding $\vec{x}$ is equivariant in H if and only if there is a representation R such that

[6] $$\vec{x}(hs)=R(h)\vec{x}(s)$$

for all h∈H and all s∈S. We also say that a model is equivariant if and only if it has an equivariant embedding. For an equivariant model whose embedding transforms as in Eq. 6, the transformation of S by any h∈H can be compensated for by the transformation of $\vec{\theta }$ by $R{\left(h\right)}^{-1⊤}$, in the sense that

[7] $$f(s;\vec{\theta })=f\left(hs;R{\left(h\right)}^{-1⊤}\vec{\theta }\right)$$

for every s∈S and every $\vec{\theta }\in {\mathbb{R}}^{M}$ (see SI Sec. 3.2). Although linear models of sequence-function relationships can be equivariant in a variety of symmetry groups H, we use the term “equivariant” to specifically refer to equivariance under $H_{\text{PSCP}}$ unless otherwise noted.

### One-hot models.

**Fig. 1.**
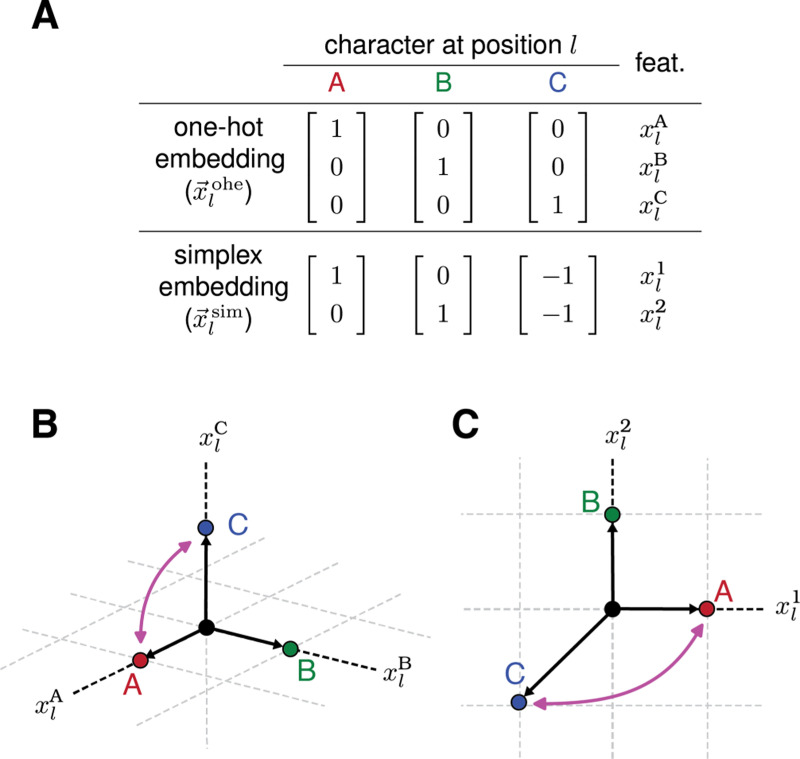
Transformation behavior of two single-position embeddings. (A) Two possible embeddings of characters at position l in a sequence built from the three-character alphabet A={A,B,C}: the three-dimensional one-hot embedding ${\vec{x}}_{l}^{\text{ohe}}$ and the two-dimensional simplex embedding ${\vec{x}}_{l}^{\text{sim}}$. The elements of ${\vec{x}}_{l}^{\text{ohe}}$ are the three one-hot sequence features $x_{l}^{\text{A}},x_{l}^{\text{B}}$, and $x_{l}^{C}$. The two elements of ${\vec{x}}_{l}^{\text{sim}}$ are denoted $x_{l}^{1}$ and $x_{l}^{2}$. (B) The embedding ${\vec{x}}^{\text{ohe}}$ for each possible character at position l. (C) The embedding ${\vec{x}}_{l}^{\text{sim}}$ for each possible character at position l. Pink arrows in panels B and C indicate the transformation of each embedding vector induced by permuting characters A and C.

The most commonly used equivariant models are based on single-position one-hot embeddings. We denote the single-position one-hot embedding for position l as ${\vec{x}}_{l}^{\text{ohe}}$ and define it to be a binary vector of dimension α with features $x_{l}^{c_{1}},\ldots ,x_{l}^{c_{\alpha }}$, where $c_{1},\ldots ,c_{\alpha }$ is an ordering of the characters in A. For example, Fig. 1A shows ${\vec{x}}_{l}^{\text{ohe}}$ for the three-character alphabet A={A,B,C}.

The one-hot embedding ${\vec{x}}_{l}^{\text{ohe}}$ transforms under a permutation representation, which we denote as $R_{l}^{\text{ohe}}$. For example, consider the transformation $h_{\text{A}↔\text{C}}$ that exchanges characters A and C at every position in a sequence. The effect of this transformation on ${\vec{x}}_{l}^{\text{ohe}}$ (Fig. 1B) is equivalent to multiplying ${\vec{x}}_{l}^{\text{ohe}}$ by the matrix

[8] $$R_{l}^{\text{ohe}}\left(h_{\text{A}↔\text{C}}\right)=\left[\begin{array}{ccc} 0 & 0 & 1\\ 0 & 1 & 0\\ 1 & 0 & 0 \end{array}\right].$$

This and all other matrices in the representation $R_{l}^{\text{ohe}}$ are permutation matrices: all matrix elements are 0 or 1, and each row and column contains a single 1. Consequently, multiplying a vector by one of these matrices changes the order of the elements in that vector, but does not change the overall set values that those elements take. We refer to ${\vec{x}}_{l}^{\text{ohe}}$ and other embeddings that transform under permutation representations as permutation embeddings; their corresponding models are called permutation models.

The embeddings of many different models can be built by taking direct sums of direct products of ${\vec{x}}_{l}^{\text{ohe}}$. For example, the pairwise one-hot model of Eq. 2 is based on the embedding

[9] $${\vec{x}}_{\text{pair}}^{\text{ohe}}={\vec{x}}^{\text{triv}}⊕\left\{\underset{l}{⊕}{\vec{x}}_{l}^{\text{ohe}}\right\}⊕\left\{\underset{l<l^{\prime }}{⊕}{\vec{x}}_{l}^{\text{ohe}}⊗{\vec{x}}_{l^{\prime }}^{\text{ohe}}\right\},$$

where ${\vec{x}}^{\text{triv}}$ denotes the trivial embedding (defined to be the 1-dimensional vector [1] for all sequences). Because ${\vec{x}}_{l}^{\text{ohe}}$ is a permutation embedding, so is ${\vec{x}}_{\text{pair}}^{\text{ohe}}$. In fact, any embedding constructed from a direct sum of direct products of ${\vec{x}}_{l}^{\text{ohe}}$ is a permutation embedding. We call this class of models the generalized one-hot models.

How a single-position embedding transforms has important consequences for how the parameters of models constructed from that embedding are interpreted. For the pairwise one-hot model, the fact that ${\vec{x}}_{l}^{\text{ohe}}$ transforms under a permutation representation imples that both ${\vec{x}}_{\text{pair}}^{\text{ohe}}$ and ${\vec{\theta }}_{\text{pair}}^{\text{ohe}}$ do as well. A consequence of this is that the individual parameters in ${\vec{\theta }}_{\text{pair}}^{\text{ohe}}$ can be interpreted as quantifying intrinsic allelic effects. For example, the transformation $h_{\text{A}↔\text{C}}$ induces a permutation of parameters that exchanges $\theta_{l}^{\text{A}}↔\theta_{l}^{\text{C}}$ at all positions l, exchanges $\theta_{ll^{\prime }}^{\text{AA}}↔\theta_{ll^{\prime }}^{CC}$ at all pairs of positions $l<l^{\prime }$, and so on. Model parameters therefore track their corresponding alleles: $\theta_{l}^{\text{A}}$ tracks sequences that have A at position l, $\theta_{ll^{\prime }}^{\text{AA}}$ tracks sequences that have AA at positions l and $l^{\prime }$, etc..

The fact that ${\vec{x}}_{l}^{\text{ohe}}$ transforms under a permutation representation also means that the features therein are not linearly independent. For example, the three embedding vectors in Fig. 1B lie within a two-dimensional affine subspace defined by the constraint $x_{l}^{A}+x_{l}^{B}+x_{l}^{C}=1$. As we will see, a consequence of such constraints is that embeddings (like ${\vec{x}}_{\text{pair}}^{\text{ohe}}$) that are built from a direct sums of multiple direct products of single-position one-hot encodings will yield models that have gauge freedoms. So although the parameters of generalized one-hot models can be interpreted as quantifying intrinsic allelic effects, the numerical values of individual parameters often cannot be interpreted in the absence of gauge-fixing constraints.

### Simplex models.

Single-position simplex embeddings encode the α characters of A using zero-centered vectors of dimension α−1. These embeddings can be defined in multiple ways that differ from one another by similarity transformations. Here we adopt a particularly convenient definition: ${\vec{x}}_{l}^{\text{sim}}(s)$ is defined to be an α−1 dimensional vector, the i’th element of which is $x_{l}^{c_{i}}(s)$ if $s_{l}\neq c_{\alpha }$ and −1 if $s_{l}=c_{\alpha }$. Fig. 1A,C illustrate ${\vec{x}}_{l}^{\text{sim}}$ for the three-character alphabet. Unlike ${\vec{x}}_{l}^{\text{ohe}}$, ${\vec{x}}_{l}^{\text{sim}}$ transforms under a non-permutation representation, which we denote as $R_{l}^{\text{sim}}$. For example, the effect of $h_{\text{A}↔\text{C}}$ on ${\vec{x}}_{l}^{\text{sim}}$ is equivalent to multiplication by the matrix

[10] $$R_{l}^{\text{sim}}\left(h_{\text{A}↔\text{C}}\right)=\left[\begin{array}{cc} -1 & 0\\ -1 & 1 \end{array}\right].$$

As with one-hot embeddings, the embeddings of many different models can be built from direct sums of direct products of ${\vec{x}}_{l}^{\text{sim}}$. For example, a simplex embedding analogous to ${\vec{x}}_{\text{pair}}^{\text{ohe}}$ can be constructed as

[11] $${\vec{x}}_{\text{pair}}^{\text{sim}}={\vec{x}}^{\text{triv}}⊕\left\{\underset{l}{⊕}{\vec{x}}_{l}^{\text{sim}}\right\}⊕\left\{\underset{l<l^{\prime }}{⊕}{\vec{x}}_{l}^{\text{sim}}⊗{\vec{x}}_{l^{\prime }}^{\text{sim}}\right\}.$$

The corresponding pairwise simplex model has the form

[12] $$f_{\text{pair}}^{\text{sim}}=\theta_{0}x_{0}+\sum_{l}\sum_{i=1}^{\alpha -1}\theta_{l}^{i}x_{l}^{i}+\sum_{l<l^{\prime }}\sum_{i,j=1}^{\alpha -1}\theta_{ll^{\prime }}^{ij}x_{ll^{\prime }}^{ij},$$

where $x_{l}^{i}$ denotes the *i*’th element of ${\vec{x}}_{l}^{\text{ohe}}$, and where $x_{ll^{\prime }}^{ij}(s)=x_{l}^{i}(s)x_{l^{\prime }}^{j}(s)$, for all s∈S. We use ${\vec{\theta }}_{\text{pair}}^{\text{sim}}$ to denote the parameters of this model. Note that these parameters are indexed using numerical superscripts ranging from 1 to α−1, rather than by characters in A.

Pairwise simplex models describe the same sequence-function relationships that pairwise one-hot model do. However, because ${\vec{x}}_{l}^{\text{sim}}$ has lower dimension than ${\vec{x}}_{l}^{\text{ohe}}$, ${\vec{\theta }}_{\text{pair}}^{\text{sim}}$ contains fewer parameters than ${\vec{\theta }}_{\text{pair}}^{\text{ohe}}$. Inspection of Eq. 11 shows that the number of parameters in ${\vec{\theta }}_{\text{pair}}^{\text{ohe}}$ is, in fact,

[13] $$\dim\;{\vec{x}}_{\text{pair}}^{\text{sim}}=1+(\alpha -1)L+\left(\begin{array}{c} L\\ 2 \end{array}\right){\left(\alpha -1\right)}^{2}.$$

This reduction in the number of parameters entirely eliminates gauge freedoms, as can be seen from the fact that

[14] $$\gamma_{\text{pair}}^{\text{ohe}}=\dim{\vec{x}}_{\text{pair}}^{\text{ohe}}-\dim{\vec{x}}_{\text{pair}}^{\text{sim}}.$$

The lack of gauge freedoms in $f_{\text{pair}}^{\text{sim}}$ is one example of the fact that, as we will see, models defined using (non-redundant) simplex embeddings do not have gauge freedoms. In fact, multiple groups (20, 22, 23) have argued for the use of simplex models, rather than one-hot models, based on the former not having gauge freedoms.

We argue, however, that the parameters of simplex models are fundamentally more difficult to interpret than are the parameters of one-hot models. Because ${\vec{x}}_{l}^{\text{sim}}$ does not transform under a permutation representation, neither does ${\vec{x}}_{\text{pair}}^{\text{sim}}$. and neither does ${\vec{\theta }}_{\text{pair}}^{\text{sim}}$. In the case of the three-character alphabet, one sees from Eq. 11 that $h_{\text{A}↔\text{C}}$ induces a transformation of model parameters that maps $\theta_{l}^{1}\to -\theta_{l}^{1},\theta_{l}^{2}\to -\theta_{l}^{1}+\theta_{l}^{2}$, $\theta_{ll^{\prime }}^{22}\to \theta_{ll^{\prime }}^{11}-\theta_{ll^{\prime }}^{12}-\theta_{ll^{\prime }}^{21}+\theta_{ll^{\prime }}^{22}$, and so on. The fact that these parameters change in a way that is not described by a permutation but rather by nontrivial linear combinations, means that individual parameters cannot be interpreted as individual allelic effects.

### Maschke decomposition of equivariant embeddings.

Having illustrated the connection between model transformation behavior, gauge freedoms, and parameter interpretability, we now use methods from the theory of group representations to formally investigate this connection. Every group representation is either reducible or irreducible. A representation is irreducible if and only if it has no proper invariant subspace. Maschke’s theorem, a foundational result in representation theory, states that all representations of finite groups are equivalent (i.e., equal up to a similarity transformation) to a direct sum of irreducible representations. Because $H_{\text{PSCP}}$ is finite, any of its representations R can be expressed as

[15] $$R≃\overset{K}{\underset{k=1}{⊕}}Q_{k}R_{k},$$

where ≃ denotes equivalence, the $R_{k}$ are pairwise inequivalent irreducible representations of $H_{\text{PSCP}}$, and each $Q_{k}$ denotes the multiplicity of $R_{k}$ in the direct sum.

In what follows, we say that a sequence embedding is irreducible if and only if it transforms under an irreducible representation of $H_{\text{PSCP}}$. One consequence of Eq. 15 is that any embedding $\vec{x}$ that transforms under R can be decomposed as

[16] $$\vec{x}≃\overset{K}{\underset{k=1}{⊕}}\overset{Q_{k}}{\underset{q=1}{⊕}}{\vec{x}}_{kq},$$

where each ${\vec{x}}_{kq}$ is an irreducible embedding that transforms under $R_{k}$.

**Fig. 2.**
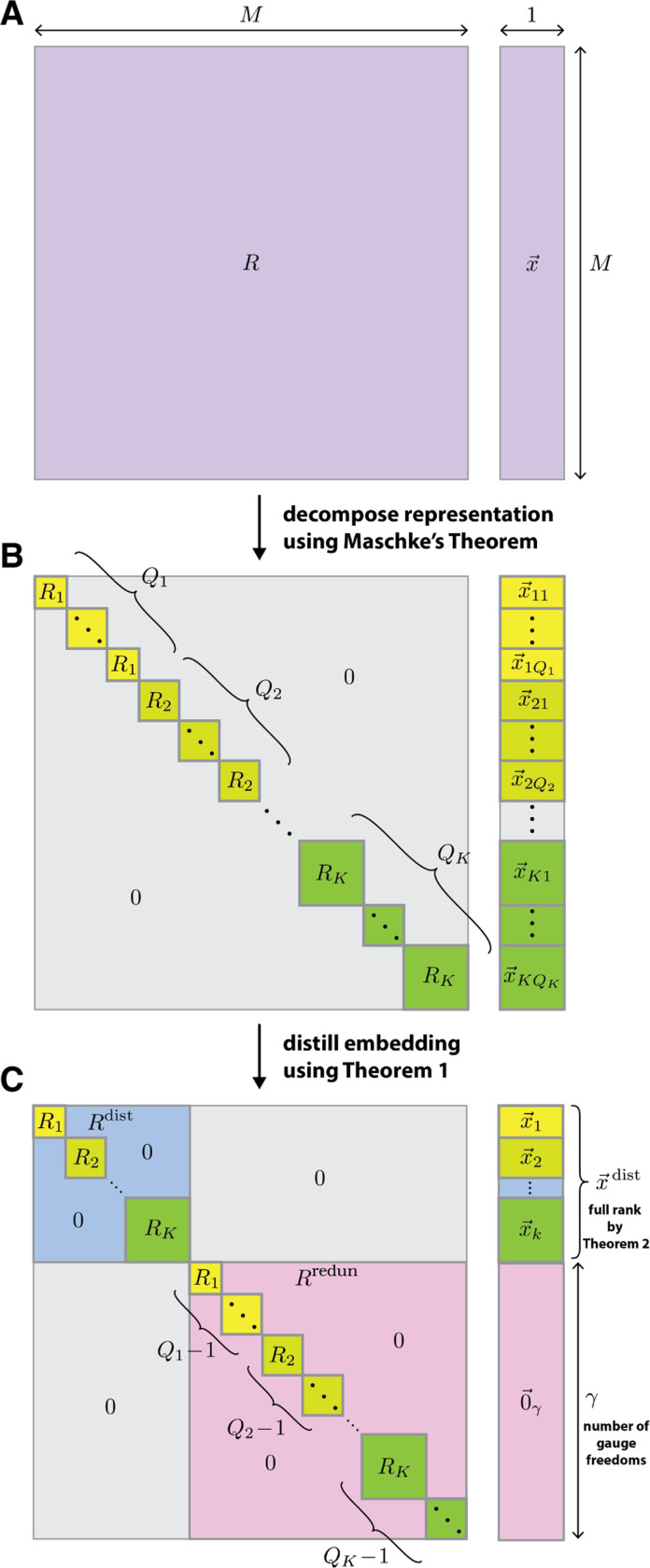
Embedding distillation. (A) Given an M-dimensional embedding $\vec{x}$ that is equivariant under $H_{\text{PSCP}}$, let R be the representation describes how single 1. Consequently, multiplying a vector by $\vec{x}$ transforms. (B) By Maschke’s theorem, R can be decomposed into a direct sum of irreducible representations, $R_{k}(k\in \{1,\ldots ,K\})$, each of which occurs with multiplicity $Q_{k}$ (Eq. 15). Similarly, $\vec{x}$ can be decomposed into a direct sum of irreducible embeddings ${\vec{x}}_{kq}\left(q\in \left\{1,\ldots ,Q_{k}\right\}\right)$, where each ${\vec{x}}_{kq}$ transforms under $R_{k}$ (Eq. 16). (C) By Theorem 1, an additional similarity transformation can be performed that, for each value of k, zeroes out all but one ${\vec{x}}_{kq}$; the remaining ${\vec{x}}_{kq}$ is denoted by ${\vec{x}}_{k}$ (Eq. 18 and Eq. 19). Consequently, $\vec{x}$ decomposes into a direct sum of a distilled embedding ${\vec{x}}^{\text{dist}}$ and a zero vector ${\vec{0}}_{\gamma }$ having dimension γ (Eq. 18). ${\vec{x}}^{\text{dist}}$ is given by the direct sum of all ${\vec{x}}_{k}$ (Eq. 19) and is full rank by Theorem 2. The distilled representation $R^{\text{dist}}$ describes how ${\vec{x}}^{\text{dist}}$ transforms and is given by a direct sum of one copy of each $R_{k}$. The redundant representation $R^{\text{redun}}$ operates on ${\vec{0}}_{\gamma }$ and comprises the $Q_{k}-1$ remaining copies of each $R_{k}$. The number of gauge freedoms γ is equal to the degree of $R^{\text{redun}}$ (Eq. 22).

This Maschke decomposition of R and $\vec{x}$ is illustrated in Fig. 2A,B. Note: here and in what follows we assume that all ${\vec{x}}_{kq}$ are nonzero, but this assumption can be removed without fundamentally changing our results; see SI Sec. 5.2 for details.

### Distillation of equivariant embeddings.

We now describe an “embedding distillation” procedure that connects the Maschke decomposition of $\vec{x}$ to the gauge freedoms of the corresponding model. In SI Sec. 5.1 we prove the following:

#### Theorem 1

*Any two nonzero sequence embeddings that transform under the same irreducible representation of* $H_{\text{PSCP}}$ *are equal up to a constant of proportionality*.

Using Theorem 1 we obtain,

[17] $$\vec{x}≃\overset{K}{\underset{k=1}{⊕}}Q_{k}{\vec{x}}_{k},$$

where ${\vec{x}}_{kq}$ is any one of the irreducible embeddings ${\vec{x}}_{k}$ in Eq. 6 and $Q_{k}$ again denotes the multiplicity of each term in the direct sum. Additional similarity transformations can then be performed to zero out all except one copy of ${\vec{x}}_{k}$. There is therefore an invertible “distillation matrix” T such that

[18] $$T\vec{x}={\vec{x}}^{\text{dist}}⊕{\vec{0}}_{\gamma }\text{,}$$

where ${\vec{0}}_{\gamma }$ is a γ-dimensional vector of zeros and

[19] $${\vec{x}}^{\text{dist}}=\overset{K}{\underset{k=1}{⊕}}{\vec{x}}_{k},$$

is the distilled embedding. When applied to the representation R, this distillation procedure yields

[20] $$TRT^{-1}={\vec{R}}^{\text{dist}}⊕{\vec{R}}^{\text{redun}},$$

where the distilled representation, $R^{\text{dist}}=\overset{K}{\underset{k=1}{⊕}}R_{k}$, comprises one copy of each $R_{k}$ present in Eq. 15, and where the redundant representation, $R^{\text{redun}}=\overset{K}{\underset{k=1}{⊕}}\left(Q_{k}-1\right)R_{k}$, comprises the remaining copies of $R_{k}$. The final distilled versions of R and $\vec{x}$ are illustrated in Fig. 2C.

### Identification of gauge freedoms in equivariant models.

To identify the gauge freedoms of any equivariant model, we use the fact that ${\vec{x}}^{\text{dist}}$ (Eq. 19) is full rank. This is a consequence of the following Theorem, which is proven in SI Sec. 3.4:

#### Theorem 2

*For each* k∈{1,…,K}, *let* ${\vec{x}}_{k}$ *be a nonzero embedding that transforms under an irreducible representation* $R_{k}$ *of the group* $H_{\text{PSCP}}$. *Then the direct sum of all* ${\vec{x}}_{k}$ *is full rank if and only if all* $R_{k}$ *are pairwise inequivalent*.

Because ${\vec{x}}^{\text{dist}}$ is full rank, ${\vec{g}}^{⊤}\vec{x}(s)=0$ for all s∈S if and only if

[21] $$\vec{g}=T^{⊤}\left[{\vec{0}}_{M-\gamma }⊕{\vec{g}}_{\gamma }\right],$$

where T is the distillation matrix in Eq. 18 and ${\vec{g}}_{\gamma }$ is any vector in ${\mathbb{R}}^{\gamma }$. The space of gauge transformations G is therefore given by the set of vectors having the form in Eq. 21. In particular, the number of gauge freedoms is seen to be

[22] $$\gamma =\dim\vec{x}-\dim{\vec{x}}^{\text{dist}}.$$

Equivalently, $\gamma =\deg R^{\text{redun}}$. We thus see that the number of gauge freedoms of an equivariant linear model is equal to the sum of the degrees of all the redundant irreducible representations under which that model’s embedding (or equivalently, parameters) transforms.

### Identification of all equivariant models.

The mathematical structure of a group defines the models that transform equivariantly under that group. In the case of $H_{\text{PSCP}}$, the relatively simple group structure allows the straight-forward identification of all inequivalent distilled embeddings, and thus all inequivalent equivariant linear models of sequence-function relationships.

$H_{\text{PSCP}}$ can be written as a product of simpler groups:

[23] $$H_{\text{PSCP}}=H_{1}\times \cdots \times H_{l},$$

where each $H_{l}$ denotes the group of character permutations at position l only. Each irreducible representation $R_{k}$ of $H_{\text{PSCP}}$ can therefore be expressed as

[24] $$R_{k}≃\overset{L}{\underset{l=1}{⊕}}R_{l}^{k},$$

where each ${\vec{x}}_{l}^{k}$ is an irreducible representation of $H_{l}$ [e.g., see Theorem 1.11.3 of (32)]. An embedding ${\vec{x}}_{k}$ that transforms under $R_{k}$ will therefore have the form

[25] $${\vec{x}}_{k}≃\overset{L}{\underset{l=1}{⊕}}{\vec{x}}_{l}^{k},$$

where ${\vec{x}}_{l}^{k}$ is an irreducible embedding that transforms under $H_{l}$. In SI Sec. 4.3 we show that $H_{l}$ supports only two inequivalent equivariant embeddings (regardless of alphabet size): ${\vec{x}}^{\text{triv}}$ and ${\vec{x}}_{l}^{\text{sim}}$. Each ${\vec{x}}_{l}^{k}$ must therefore be one of these two embeddings. Ignoring factors of ${\vec{x}}^{\text{triv}}$ which do not alter direct products, Eq. 25 can therefore be written as

[26] $${\vec{x}}_{k}=\underset{l\in B_{k}}{⊕}{\vec{x}}_{l}^{\text{sim}},$$

where $B_{k}$ is a subset of the positions {1,…,L}. There are $2^{L}$ possible choices for each subset $B_{k}$, and thus $2^{L}$ inequivalent irreducible embeddings ${\vec{x}}_{k}$. Since each ${\vec{x}}_{k}$ can appear at most once on the left-hand side of Eq. 19, we find that there are $2^{2^{L}}$ inequivalent distilled embeddings ${\vec{x}}^{\text{dist}}$.

For each choice of ${\vec{x}}^{\text{dist}}$ there are an infinite number of possible choices for T and γ that can be used, via Eq. 18, to define $\vec{x}$. The number of possible equivariant embeddings $\vec{x}$, and thus the number of equivariant models f, is therefore infinite. However, all models corresponding to a specific ${\vec{x}}^{\text{dist}}$ have the same expressivity, i.e., the set of sequence-function relationships that each model describes (considered over all possible values of model parameters) is the same. We therefore consider these models to be equivalent, and conclude that there are a total of $2^{2^{L}}$ inequivalent equivariant linear models of sequence-function relationships.

### Analytical analysis of generalized one-hot models.

We now use the embedding distillation procedure to compute the number of gauge freedoms of all generalized one-hot models. This derivation is based on the Maschke decomposition of ${\vec{x}}_{l}^{\text{ohe}}$, which is see SI Sec. 2.5 for details.

[27] $${\vec{x}}_{l}^{\text{ohe}}≃{\vec{x}}^{\text{triv}}⊕{\vec{x}}_{l}^{\text{sim}};$$

see SI Sec. 2.5 for details.

We first demonstrate this analysis on the pairwise one-hot model. Plugging the decomposition of ${\vec{x}}_{l}^{\text{ohe}}$ in Eq. 27 into the definition for ${\vec{x}}_{\text{pair}}^{\text{ohe}}$ in Eq. 9, then expanding the direct product and grouping like terms, we find that

[28] $${\vec{x}}_{\text{pair}}^{\text{ohe}}≃\left[1+L+\left(\begin{array}{c} L\\ 2 \end{array}\right)\right]{\vec{x}}^{\text{triv}}⊕\left\{\underset{l}{⊕}L{\vec{x}}_{l}^{\text{sim}}\right\}⊕\left\{\underset{l<l^{\prime }}{⊕}{\vec{x}}_{l}^{\text{sim}}⊗{\vec{x}}_{l^{\prime }}^{\text{sim}}\right\},$$

where the scalar coefficients correspond to the $Q_{k}$ in Eq. 17. We derive the corresponding distilled embedding by simply replacing each of these coefficients with 1. Doing so reveals the distillation of ${\vec{x}}_{\text{pair}}^{\text{ohe}}$ to be ${\vec{x}}_{\text{pair}}^{\text{sim}}$. The result for $\gamma_{\text{pair}}^{\text{ohe}}$ in Eq. 14 is therefore just a manifestation of Eq. 22.

We now extend this analysis approach to all generalized one-hot models. The embedding ${\vec{x}}_{\text{goh}}$ of any generalized one-hot model can be written as

[29] $${\vec{x}}_{\text{goh}}=\overset{J}{\underset{j=1}{⊕}}\underset{l\in A_{j}}{⊕}{\vec{x}}_{l}^{\text{ohe}}$$

where $A_{1},\ldots ,A_{J}$ denote J (not necessarily distinct) sets of positions. Because the dimension of ${\vec{x}}_{l}^{\text{ohe}}$ is α, the number of corresponding model parameters is

[30] $$M_{\text{goh}}=\dim{\vec{x}}_{\text{goh}}=\sum_{j=1}^{J}\alpha^{\left|A_{j}\right|}.$$

Again, using Eq. 27 to decompose ${\vec{x}}_{l}^{\text{ohe}}$ in terms of ${\vec{x}}^{\text{triv}}$ and ${\vec{x}}_{l}^{\text{sim}}$, then expanding each tensor product and grouping the resulting terms, we find that

[31] $${\vec{x}}_{\text{goh}}^{\text{dist}}=\overset{K}{\underset{k=1}{⊕}}\underset{l\in B_{k}}{⊕}{\vec{x}}_{l}^{\text{sim}},$$

where $B_{1},\ldots ,B_{K}$ denote the distinct subsets of positions that occur among the $A_{j}$. Because the dimension of ${\vec{x}}_{l}^{\text{sim}}$ is α−1,

[32] $$\dim{\vec{x}}_{\text{goh}}^{\text{dist}}=\sum_{k=1}^{K}{\left(\alpha -1\right)}^{\left|B_{k}\right|}.$$

The number of gauge freedoms of the generalized one-hot model having embedding ${\vec{x}}_{\text{goh}}$ is therefore given by

[33] $$\gamma_{\text{goh}}=\sum_{j=1}^{J}\alpha^{\left|A_{j}\right|}-\sum_{k=1}^{K}{\left(\alpha -1\right)}^{\left|B_{k}\right|}.$$

**Fig. 3.**
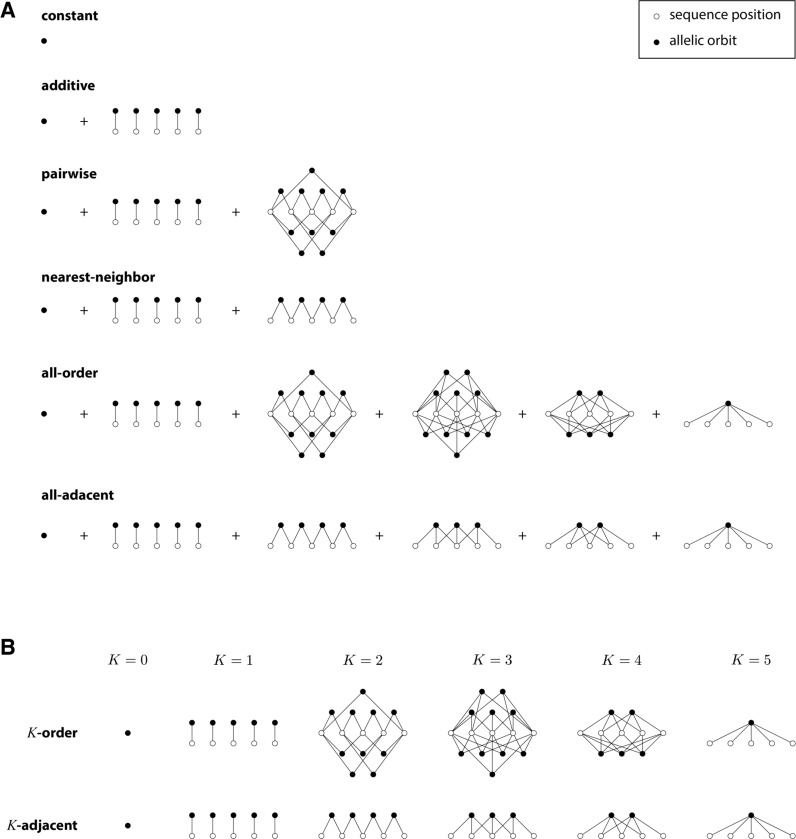
Structure of generalized one-hot models analyzed in Table 1 for sequences of length L=5. Open circles represent sequence positions. Closed circles represent allelic orbits, i.e., sets of sequence features that are closed under the action of $H_{\text{PSCP}}$. Edges indicate position indices shared by the features in each allelic orbit. (A) Structure of constant, additive, pairwise, nearest-neighbor, all-order, and all-adjacent models. (B) Structure of K-order models and K-adjacent models for various interaction orders K.

Table 1 reports the number of gauge freedoms computed in this manner for a variety of generalized one-hot models (illustrated in Fig. 3). SI Sec. 6 provides expanded descriptions for each generalized one-hot model, as well as detailed computations of the results in Table 1.

A result of this analytic analysis is that all generalized one-hot models have gauge freedoms, save models for which the direct sum in Eq. 29 includes only one term. To see this, observe that Eq. 17 gives

[34] $$\dim{\vec{x}}_{\text{goh}}=\sum_{k=1}^{K}Q_{k}{\left(\alpha -1\right)}^{\left|B_{k}\right|}\text{,}$$

where each multiplicity value $Q_{k}$ is equal to the number of sets $A_{j}$ that contain $B_{k}$. Using this together with Eq. 31 in Eq. 22 gives

[35] $$\gamma_{\text{goh}}=\sum_{k=1}^{K}\left(Q_{k}-1\right){\left(\alpha -1\right)}^{\left|B_{k}\right|}.$$

We thus see that $\gamma_{\text{goh}}=0$ if and only if none of the $Q_{k}$ are greater than 1. But the empty set is a subset of every $A_{j}$. The empty set will therefore always be among the $B_{k}$, and the corresponding multiplicity value will be $Q_{k}=J$. Gauge freedoms are therefore present in all generalized one-hot models for which J≥2. Conversely, $\gamma_{\text{goh}}=0$ when J=1 because all $B_{k}$ occur with multiplicity $Q_{k}=1$. Gauge freedoms are therefore absent in all generalized one-hot models for which J=1.

### Computational analysis of generalized one-hot models.

Embedding distillation also allows one to efficiently compute a sparse basis for the space of gauge freedoms, $G_{\text{goh}}$, of any generalized one-hot model. Eq. 21 reveals that $G_{\text{goh}}$ is spanned by the last $\gamma_{\text{goh}}$ row vectors of T. One can therefore compute a basis for $G_{\text{goh}}$ by computing T. This is notable because and computing T only requires keeping track of the similarity transformations needed to express ${\vec{x}}_{\text{goh}}$ in the distilled form shown in Eq. 18. This computation is far less demanding than computing a basis for $G_{\text{goh}}$ using Gaussian elimination or singular value decomposition when (as is often the case) the number of possible sequences is far greater than the number of model parameters.

We now describe an algorithm for distilling any generalized one-hot embedding ${\vec{x}}_{\text{goh}}$. Eq. 27 can be written as an equality, $T^{\left(1\right)}{\vec{x}}_{l}^{\text{ohe}}={\vec{x}}^{\text{triv}}⊕{\vec{x}}_{l}^{\text{sim}}$, where $T^{\left(1\right)}$ is the α×α matrix

[36] $$T^{\left(1\right)}=\left[\begin{array}{ccccc} 1 & 1 & \cdots & 1 & 1\\ 1 & 0 & \cdots & 0 & -1\\ 0 & 1 & \cdots & 0 & -1\\ \vdots & \vdots & \ddots & \vdots & \vdots \\ 0 & 0 & \cdots & 1 & -1 \end{array}\right].$$

Using $T^{\left(1\right)}$, one can compute the distillation matrix T for any generalized one-hot model as the product of three matrices:

[37] $$T=T_{\text{sort}}T_{\text{thin}}T_{\text{decom}}.$$

**Fig. 4.**
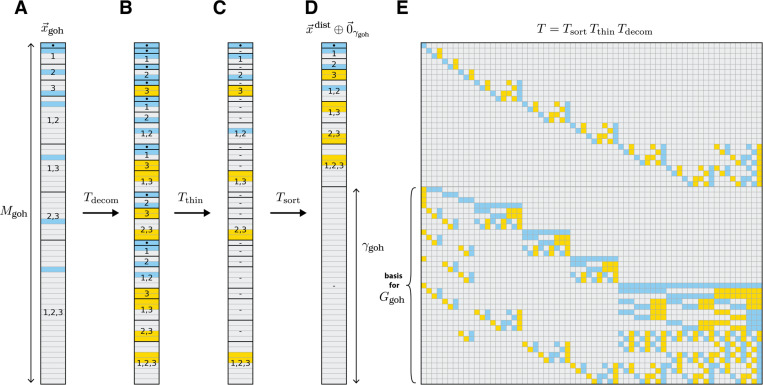
Embedding distillation for an example generalized one-hot model. (A) Embedding ${\vec{x}}_{\text{goh}}$ of the L=3 sequence s=ABC for an all-order interaction model based on the alphabet A={A,B,C}. Embedding has degree $M_{\text{goh}}=64$. (B) Result of multiplication by the decomposition matrix, $T_{\text{decom}}$. (C) Result of subsequent multiplication by the thinning matrix $T_{\text{thin}}$. (D) Result of subsequent multiplication by the sorting matrix $T_{\text{sort}}$, which yields ${\vec{x}}^{\text{dist}}⊕{\vec{0}}_{\gamma_{\text{goh}}}$ with $\gamma_{\text{goh}}=37$ gauge freedoms. In B-D, dots indicate ${\vec{x}}^{\text{triv}}$, dashes indicate zero vectors, and numbers indicate ${\vec{x}}_{l}^{\text{sim}}$ or Kronecker products thereof for specified positions l. (E) Distillation matrix T that implements the full distillation procedure in A-D. The last $\gamma_{\text{goh}}$ rows of T provide a sparse basis for the gauge space, $G_{\text{goh}}$. In A-E, vector and matrix elements are colored using: blue, +1; yellow, −1; gray, 0.

The effects of these three matrices are illustrated in Fig. 4. The “decomposition matrix”, $T_{\text{decom}.}$, decomposes ${\vec{x}}_{\text{goh}}$ (Fig. 4A) into a direct sum of irreducible embeddings (Fig. 4B). The “thinning matrix”, $T_{\text{thin}}$, then zeros out all except the first copy of each irreducible embedding (Fig. 4C). The “sorting matrix”, $T_{\text{sort}}$, then rearranges the direct sum so that the remaining nonzero embeddings come first, followed by a zero vector of dimension $\gamma_{\text{goh}}$ (Fig. 4D). SI Sec. 8 provides explicit algorithms for constructing $T_{\text{decom}}$, $T_{\text{thin}}$, and $T_{\text{sort}}$ as well as the inverse of each of these three matrices. It is readily seen that all of these matrices are sparse in the large L limit when the maximal order of interaction described by the model is fixed. The resulting distillation matrix T as well as its inverse are therefore also sparse. Moreover, every nonzero element of T is +1 or −1 (Fig. 4E). Taking the last $\gamma_{\text{goh}}$ rows of T we obtain a basis for $G_{\text{goh}}$comprising sparse vectors whose only nonzero elements are +1 and −1. These sparse forms for the factors of T and $T^{-1}$ also allows us to compute a sparse gauge-fixing projection matrix P; see SI Sec. 8 for details.

### Other symmetry groups.

The proof of Theorem 1 in SI Sec. 5.1, and thus our embedding distillation procedure, applies only to the symmetry group $H_{\text{PSCP}}$. There are other symmetry groups of sequence space besides $H_{\text{PSCP}}$, however, and it is worth asking whether Theorem 1, and thus Eqs. 18–22, hold for those groups as well.

One other symmetry group is the group of global character permutations, $H_{\text{GCP}}$. This group comprises transformations that affect the same permutation of characters at every position. Another is the group of position permutations, $H_{\text{PP}}$. This group comprises transformations that permute positions without otherwise changing characters. SI Sec. 7.1 shows that Theorem 1 does not hold for either $H_{\text{GCP}}$ or $H_{\text{PP}}$. Consequently, one cannot compute distilled embeddings using the irreducible representations of these groups.

A third symmetry group is $H_{\text{Ham}}$, which describes combinations of position permutations and position-specific character permutations. $H_{\text{Ham}}$ is the largest symmetry group that preserves Hamming distances (33), and includes $H_{\text{PSCP}}$, $H_{\text{PP}}$, and $H_{\text{GCP}}$ as subgroups. Theorem 1 does hold for $H_{\text{Ham}}$, due the fact that $H_{\text{PSCP}}$ is a subgroup (see SI Sec. 7.2). However, the set of models that are equivariant under $H_{\text{Ham}}$ is a subset of the models that are equivariant under $H_{\text{PSCP}}$, and the irreducible representations of $H_{\text{Ham}}$ are more complex than those of $H_{\text{PSCP}}$. $H_{\text{PSCP}}$ is therefore more useful than $H_{\text{Ham}}$ is for analyzing gauge freedoms.

### Transformation behavior and parameter interpretability.

We now return to the connection between parameter transformation behavior and parameter interpretability. Above we observed for pairwise models that the ability to interpret individual parameters as quantifying intrinsic allelic effects appears to require the presence of gauge freedoms. We now formalize this observation and conjecture an extension to all linear models.

We define an allele a to be to be a pattern of characters that is either present or absent in any sequence s∈S. The corresponding “allelic feature” $x_{a}$ is defined be the indicator function on S for whether a sequence has allele a, and an “allelic model” is defined to be a linear sequence-function relationship in which every feature is an allelic feature. In the context of an allelic model, the parameter $\theta_{a}$ that multiplies $x_{a}$ is said to be an “allelic effect” The parameters of a linear model can therefore be interpreted as allelic effects if and only if every one of the corresponding features is an indicator function on S.

For an allelic model to have parameters that describe intrinsic allelic effects, the model must be a “permutation model”, i.e., the features and parameters of the model must transform under a permutation representation of $H_{\text{PSCP}}$. Requiring an allelic model to be a permutation model puts strong constraints on which sets of alleles it can describe. Because $H_{\text{PSCP}}$ permutes sequences, it also permutes alleles. Given a specific allele a, we call the set of alleles created by the action of $H_{\text{PSCP}}$ on a an “allelic orbit”. It is readily seen that, for an allelic model to be a permutation model, the set of alleles it describes must consist of an integral number J of complete allelic orbits.

All allelic models that comprise J≥2 allelic orbits have gauge freedoms. To see this, observe that the features in each orbit transform among themselves according to a permutation representation. The features of the full model will therefore transform under a direct sum of J permutation representations. Because every permutation representation contains the trivial representation in its Maschke decomposition, the decomposition of the full model’s representation will contain at least J copies of the trivial representation. The model will therefore have at least J−1 gauge freedoms. Additional gauge freedoms can be present as well, so this result only provides a lower bound on the number of gauge freedoms.

This result is reflected in our above analytic analysis of generalized one-hot models. All generalized one-hot models are allelic permutation models (though the converse is not true; see SI Sec. 9.1), and each allelic orbit of a generalized one-hot model corresponds to a position set $A_{j}$ in Eq. 29. The lower-bound on the number of gauge freedoms identified here recapitulates the finding above that generalized one-hot models have no gauge freedoms if and only if J=1.

An allelic permutation model that does not have gauge freedoms must therefore comprise only one allelic orbit. Are single-orbit allelic models useful in practice? We argue that the answer is essentially “no”. In SI Sec. 9.1 we show that single-orbit generalized one-hot models cannot describe co-occurring alleles. We regard such models as “trivial” because the entire reason for quantitatively modeling sequence-function relationships is to deconvolve the influence of co-occurring alleles. There are single-orbit allelic permutation that describe co-occurring alleles, but all the examples of these we have analyzed either have gauge freedoms or are mathematically equivalent to generalized one-hot models (see SI Sec. 9.1). Moreover, among models whose embeddings are built from direct sums of direct products of single-position embeddings, the generalized one-hot models have the fewest gauge freedoms (see SI Sec. 9.2). Based on these findings, we conjecture that all nontrivial allelic permutation models (i.e., all models whose parameters describe intrinsic allelic effects) have gauge freedoms.

## Discussion

Motivated by the connection between gauge freedoms and symmetries in physics, we investigated the relationship between gauge freedoms and symmetries in quantitative models of sequence-function relationships. We found that, for linear models that are equivariant under the group of position-specific character permutations (denoted $H_{\text{PSCP}}$), gauge freedoms arise due to model parameters transforming according to redundant irreducible matrix representations. From a practical standpoint, this result facilitates the analytic calculation of the number of independent gauge freedoms, as well as the efficient computation of a sparse basis for the space of gauge freedoms, in a large class of commonly used models. From a conceptual standpoint, this result links the gauge freedoms of models of sequence-function relationships to the transformation behavior of these models under a specific symmetry group of sequence space.

We also investigated the link between parameter transformation behavior and parameter interpretability. In doing so, we identified an incompatibility between two different notions of parameter interpretability: in linear models that are equivariant under $H_{\text{PSCP}}$, the ability to interpret individual parameters as quantifying intrinsic allelic effects requires that these parameters transform under a permutation representation of $H_{\text{PSCP}}$. But in many (and possibly in all) nontrivial models, this requirement is incompatible with the ability to interpret the values of individual parameters in the absence of gauge-fixing constraints. Consequently, models that have gauge freedoms can have advantages over equally expressive models that do not have gauge freedoms.

It should be noted that there are indeed useful models that do not have gauge freedoms. One such class of models are the “wild-type” one-hot models, the features of which are limited to those describing mutations away from a specific sequence of interest (e.g., 34, 35). While the parameters of such models do quantify allelic effects, they do so relative to a specific reference sequence, and so do not quantify intrinsic allelic effects. Another class of useful models that do not have gauge freedoms are models whose features represent sequence-dependent physical properties, such as the chemical properties of amino acids (36, 37) or the physical shape of the DNA double helix (38, 39). These models are not equivariant, however, and their parameters describe the effects of physical properties of alleles, not the effects of alleles themselves. Notably, both classes of model reflect inductive biases that break the symmetry described by $H_{\text{PSCP}}$.

We now return to the analogy with theoretical physics. In classical field theories like E&M, there are specific symmetries that are well-established by experiment and that any mathematical formulation of the theory must be consistent with. This does not, however, mean that the equations of the theory must transform in a simple way under those symmetries. Mathematically formulating physical theories so that the equations themselves manifestly respect the symmetries of the theory generally requires over-parameterizing the equations, thereby introducing gauge freedoms. Physicists often find it worthwhile to do this, as having fundamental symmetries be manifestly reflected in one’s equations can greatly facilitate the interpretation and application of those equations. Solving such equations, however, requires fixing the gauge—introducing additional constraints that make the solution of the equations unique.

Unlike in physics, there is no experimentally established requirement that models of sequence-function relationships be equivariant under any symmetries of sequence space. The specific mathematical form one uses for such models is subjective, and different models are commonly used in different contexts. Citing the ambiguities caused by gauge freedoms, some have argued for restricting one’s choice of model to those that have no gauge freedoms. Nevertheless, models that have gauge freedoms are still common in the literature. We suggest that a major reason for this may be that researchers often prefer to use models that manifestly reflect symmetries of sequence space, and therefore have parameters that are interpretable as intrinsic allelic effects. As we showed, these criteria often (and possibly in all nontrivial cases) require the use of over-parameterized models. In this way, the origin of gauge freedoms in models of sequence-function relationships does mirror the origin of gauge freedoms in physical theories.

There is still much to understand about the relationship between models of sequence-function relationships, the symmetries of these models, and how these modes can be biological interpreted. This paper and its companion (31) have only addressed gauge freedoms and symmetries in linear models of sequence-function relationships. Some work has explored the gauge freedoms and symmetries of nonlinear models of sequence-function relationships (40, 41), but important questions remain. The sloppy modes (42, 43) present in sequence-function relationships are also important to understand but, to our knowledge, these have yet to be systematically investigated. Addressing these problems is becoming increasingly urgent due to the expanding interest in interpretable quantitative models of sequence-function relationships (e.g., 44).

## Materials and Methods

See Supplemental Information for full derivations of the mathematical results. Python code implementing the embedding distillation algorithm described the section “Computational analysis of generalized one-hot models” and used for generating Fig. 4 is available at https://github.com/jbkinney/23_posfai.

## Supplementary Material

Supplement 1

## Figures and Tables

**Table 1.** Analytical results for various generalized one-hot models, computed using Eqs. 30 and 33. Columns show model type, the orders of interaction included in each model, the number of parameters of each model, and the number of gauge freedoms of each model. See SI Sec. 6 for derivations of these results. K-adjacent models assume K≥1.

model type	interaction orders	no. parameters ($M_{\text{goh}}$)	no. gauge freedoms ($\gamma_{\text{goh}}$)
constant	0	1	0
additive	0,1	1+Lα	L
pairwise	0,1,2	$1+L\alpha +\left(\begin{array}{c} L\\ 2 \end{array}\right)\alpha^{2}$	$L+\left(\begin{array}{c} L\\ 2 \end{array}\right)(2\alpha -1)$
nearest-neighbor	0,1,2^‡^	$1+L\alpha +(L-1)\alpha^{2}$	L+(L−1)(2α−1)
all-order	0,1,...,*L*	${\left(\alpha +1\right)}^{L}$	${\left(\alpha +1\right)}^{L}-\alpha^{L}$
all-adjacent	0,1,2^‡^,...,*L*^‡^	$1+\frac{\alpha }{{\left(\alpha -1\right)}^{2}}\left[\alpha^{L+1}-(L+1)\alpha +L\right]$	$1+\frac{\alpha }{{\left(\alpha -1\right)}^{2}}\left[2\alpha^{L}-\alpha^{L-1}-(L+1)\alpha +L\right]$

K-order	K	$\left(\begin{array}{c} L\\ K \end{array}\right)\alpha^{K}$	$\left(\begin{array}{c} L\\ K \end{array}\right)\alpha^{K}-\sum_{k=0}^{K}\left(\begin{array}{c} L\\ K \end{array}\right){\left(\alpha -1\right)}^{k}$
hierarchical K-order	0,1,...,*K*	$\sum_{k=0}^{K}\left(\begin{array}{c} L\\ k \end{array}\right)\alpha^{k}$	$\sum_{k=0}^{K}\left(\begin{array}{c} L\\ K \end{array}\right)\left[\alpha^{k}-{\left(\alpha -1\right)}^{k}\right]$

K-adjacent^‡^	K ^‡^	$(L-K+1)\alpha^{K}$	$(L-K)\alpha^{K-1}$
hierarchical K-adjacent^‡^	0,1,2^‡^,...,*K*^‡^	$1+\sum_{k=1}^{K}(L-k+1)\alpha^{k}$	$(L-K)\alpha^{K-1}+1+\sum_{k=1}^{K-1}(L-k+1)\alpha^{k}$

‡ Only includes interactions among adjacent positions.

## Significance Statement

Gauge freedoms—directions in parameter space that do not affect model predictions—are ubiquitous in mathematical models of biological sequence-function relationships. But in contrast to theoretical physics, where gauge freedoms play a central role, little is understood about the mathematical properties of gauge freedoms in models of sequence-function relationships. Here we identify a connection between specific symmetries of sequence space and the gauge freedoms present in a large class of commonly used models for sequence-function relationships. We show that this connection can be used to perform useful mathematical computations, and we discuss the impact of model transformation properties on parameter interpretability. The results fill a major gap in the understanding of quantitative sequence-function relationships.

## Footnotes

* Results in quantum physics, such as the Aharanov-Bohm effect (28, 29), suggest a reality to the four-potential beyond what can be inferred solely from classical E&M, though there are arguments against this interpretation (30).

† Our analysis is readily extended to models in which $\vec{x}$ and $\vec{\theta }$ are complex vectors (e.g. 24). All the results in Supplemental Information are, in fact, derived for this more general class of models. Here we restrict our discussion to the reals only to simplify the presentation.

## References

[1] Kinney JB, McCandlish DM, Massively parallel assays and quantitative sequence-function relationships. Annu. Rev. Genomics Hum. Genet. 20, 99–127 (2019) Wrote. 31091417 10.1146/annurev-genom-083118-014845

[2] Kinney JB, Tkacik G, Callan CG, Precise physical models of protein-DNA interaction from high-throughput data. Proc. Natl. Acad. Sci. 104, 501–506 (2007) Wrote. 17197415 10.1073/pnas.0609908104 PMC1766414

[3] Weigt M, White RA, Szurmant H, Hoch JA, Hwa T, Identification of direct residue contacts in protein-protein interaction by message passing. Proc. Natl. Acad. Sci. 106, 67–72 (2009). 19116270 10.1073/pnas.0805923106 PMC2629192

[4] Marks DS, Protein 3D Structure Computed from Evolutionary Sequence Variation. PLoS ONE 6, e28766 (2011). 22163331 10.1371/journal.pone.0028766 PMC3233603

[5] Ekeberg M, Lovkvist C, Lan Y, Weigt M, Aurell E, Improved contact prediction in proteins: Using pseudolikelihoods to infer Potts models. Phys. Rev. E 87, 012707 (2013). 10.1103/PhysRevE.87.012707 23410359

[6] Ekeberg M, Hartonen T, Aurell E, Fast pseudolikelihood maximization for direct-coupling analysis of protein structure from many homologous amino-acid sequences. J. Comput. Phys. 276, 341–356 (2014).

[7] Stein RR, Marks DS, Sander C, Inferring Pairwise Interactions from Biological Data Using Maximum-Entropy Probability Models. PLoS Comput. Biol. 11, e1004182 (2015). 26225866 10.1371/journal.pcbi.1004182 PMC4520494

[8] Barton JP, Leonardis ED, Coucke A, Cocco S, ACE: adaptive cluster expansion for maximum entropy graphical model inference. Bioinformatics 32, 3089–3097 (2016). 27329863 10.1093/bioinformatics/btw328

[9] Haldane A, Flynn WF, He P, Levy RM, Coevolutionary Landscape of Kinase Family Proteins: Sequence Probabilities and Functional Motifs. Biophys. J. 114, 21–31 (2018). 29320688 10.1016/j.bpj.2017.10.028 PMC5773752

[10] Cocco S, Feinauer C, Figliuzzi M, Monasson R, Weigt M, Inverse statistical physics of protein sequences: a key issues review. Reports on Prog. Phys. 81, 032601 (2018). 10.1088/1361-6633/aa9965 29120346

[11] A Haldane, Levy RM, Influence of multiple-sequence-alignment depth on Potts statistical models of protein covariation. Phys. Rev. E 99, 032405 (2019). 30999494 10.1103/PhysRevE.99.032405 PMC6508952

[12] Rube HT, Probing molecular specificity with deep sequencing and biophysically interpretable machine learning. bioRxivp. 2021.06.30.450414 (2021).

[13] Zamuner S, Rios PDL, Interpretable Neural Networks based classifiers for categorical inputs. arXiv (2021).

[14] Feinauer C, Meynard-Piganeau B, Lucibello C, Interpretable pairwise distillations for generative protein sequence models. PLoS Comput. Biol. 18, e1010219 (2022). 35737722 10.1371/journal.pcbi.1010219 PMC9258900

[15] Gerardos A, Dietler N, Bitbol AF, Correlations from structure and phylogeny combine constructively in the inference of protein partners from sequences. PLoS Comput. Biol. 18, e1010147 (2022). 35576238 10.1371/journal.pcbi.1010147 PMC9135348

[16] Hsu C, Nisonoff H, Fannjiang C, Listgarten J, Learning protein fitness models from evolutionary and assay-labeled data. Nat. Biotechnol. 40,1114–1122 (2022). 35039677 10.1038/s41587-021-01146-5

[17] Feinauer C, Borgonovo E, Mean Dimension of Generative Models for Protein Sequences. bioRxiv p. 2022.12.12.520028 (2022).

[18] Rube HT, Prediction of protein-ligand binding affinity from sequencing data with interpretable machine learning. Nat. Biotechnol. 40,1520–1527 (2022). 35606422 10.1038/s41587-022-01307-0 PMC9546773

[19] Weinberger ED, Fourier and taylor series on fitness landscapes. Biol. cybernetics 65, 321–330 (1991).

[20] Zhang CT, Zhang R, Analysis of distribution of bases in the coding sequences by a diagrammatic technique. Nucleic acids research 19, 6313–7 (1991). 1956790 10.1093/nar/19.22.6313 PMC329145

[21] Stadler PF, Spectral landscape theory in Evolutionary Dynamics: Exploring the Interplay of Selection, Accident, Neutrality and Function, eds. Crutchfield J Schuster P. (Oxford Univ. Press, Oxford), pp. 231–271 (2003).

[22] Stormo GD, Maximally efficient modeling of DNA sequence motifs at all levels of complexity. Genetics 187,1219–1224 (2011-04). 21300846 10.1534/genetics.110.126052 PMC3070529

[23] Poelwijk FJ, Krishna V, Ranganathan R, The Context-Dependence of Mutations: A Linkage of Formalisms. PLOS Comput. Biol. 12, e1004771 (2016). 27337695 10.1371/journal.pcbi.1004771 PMC4919011

[24] Brookes DH, Aghazadeh A, Listgarten J, On the sparsity of fitness functions and implications for learning. Proc. Natl. Acad. Sci. 119, e2109649118 (2022). 34937698 10.1073/pnas.2109649118 PMC8740588

[25] Tareen A, MAVE-NN: learning genotype-phenotype maps from multiplex assays of variant effect. Genome Biol. 23, 98 (2022). 35428271 10.1186/s13059-022-02661-7 PMC9011994

[26] Jackson JD, Okun LB, Historical roots of gauge invariance. Rev. Mod. Phys. 73, 663–680 (2001).

[27] Jackson JD, Classical electrodynamics. (John Wiley & Sons), (1998).

[28] Y Aharonov, Bohm D, Significance of electromagnetic potentials in the quantum theory. Phys. review 115, 485 (1959).

[29] Peshkin M, Tonomura A, The Aharonov-Bohm Effect. (Springer Verlag), (2005).

[30] Vaidman L, Role of potentials in the aharonov-bohm effect. Phys. Rev. A 86, 040101 (2012).

[31] Posfai A, Zhou J, McCandlish DM, Kinney JB, Gauge fixing for sequence-function relationships. bioRxiv (2024). 10.1371/journal.pcbi.1012818 PMC11957564 40111986

[32] Sagan BE, The Symmetric Group: Representations, Combinatorial Algorithms, and Symmetric Functions, Graduate Texts in Mathematics. (Springer), 2 edition, (2001) Read in early 2022.

[33] Happel R, Stadler PF, Canonical approximation of fitness landscapes. Complexity 2, 53–58 (1996).

[34] Olson CA, Wu NC, Sun R, A comprehensive biophysical description of pairwise epistasis throughout an entire protein domain. Curr. biology: CB 24, 2643–2651 (2014). 25455030 10.1016/j.cub.2014.09.072 PMC4254498

[35] Baeza-Centurion P, Miñana B, Schmiedel JM, Valcárcel J, Lehner B, Combinatorial genetics reveals a scaling law for the effects of mutations on splicing. Cell 176, 549–563.e23 (2019). 30661752 10.1016/j.cell.2018.12.010

[36] Kawashima S, Aaindex: amino acid index database, progress report 2008. Nucleic acids research 36, D202–D205 (2007). 17998252 10.1093/nar/gkm998 PMC2238890

[37] Yang KK, Wu Z, Bedbrook CN, Arnold FH, Learned protein embeddings for machine learning. Bioinformatics 34, 2642–2648 (2018). 29584811 10.1093/bioinformatics/bty178 PMC6061698

[38] Rohs R, The role of DNA shape in protein-DNA recognition. Nature 461, 1248–1253 (2009). 19865164 10.1038/nature08473 PMC2793086

[39] Yang L, TFBSshape: a motif database for DNA shape features of transcription factor binding sites. Nucl Acids Res 42, D148–D155 (2013). 24214955 10.1093/nar/gkt1087 PMC3964943

[40] Kinney JB, Atwal GS, Parametric Inference in the Large Data Limit Using Maximally Informative Models. Neural computation 26, 637–653 (2014-04) Wrote. 24479782 10.1162/NECO_a_00568

[41] Atwal GS, Kinney JB, Learning Quantitative Sequence-Function Relationships from Massively Parallel Experiments. J. Stat. Phys. 162, 1203–1243 (2016) Wrote.

[42] Machta BB, Chachra R, Transtrum MK, Sethna JP, Parameter space compression underlies emergent theories and predictive models. Science 342, 604–607 (2013). 24179222 10.1126/science.1238723

[43] Transtrum MK, Perspective: Sloppiness and emergent theories in physics, biology, and beyond. The J. Chem. Phys. 143, 010901–14 (2015). 26156455 10.1063/1.4923066

[44] Seitz E, McCandlish DM, Kinney JB, Koo PK, Interpreting cis-regulatory mechanisms from genomic deep neural networks using surrogate models. bioRxiv (2023). 10.1038/s42256-024-00851-5 PMC11823438 39950082

