# Supplementary material for: Symmetry, gauge freedoms, and the interpretability of sequence-function relationships
Source: bioRxiv. 2024 May 13:2024.05.12.593774. Preprint. [Version 1] doi: 10.1101/2024.05.12.593774 (PMC11118426; doi:10.1101/2024.05.12.593774)
Supplement: Supplementary file 1 [file media-1.pdf]

# Supplemental Information for:
# Symmetry, gauge freedoms, and the interpretability of sequence-function relationships

Anna Posfai, David M. McCandlish, Justin B. Kinney

May 13, 2024

## Contents


**1 Preliminaries** **2**
- 1.1 General notation . . . 2
- 1.2 Specific sets . . . 2
- 1.3 Specific groups . . . 2
- 1.4 Specific embeddings . . . 3
- 1.5 Definitions . . . 3

**2 Example: Embeddings and representations for single DNA characters** **4**
- 2.1 The one-hot embedding $\vec{x}^{\,\text{ohe}}$ and the defining representation $R^{\,\text{def}}$ . . . 4
- 2.2 The trivial embeddings $\vec{x}^{\,\text{triv}}$ and the trivial representaiton $R^{\,\text{triv}}$ . . . 4
- 2.3 The simplex embedding $\vec{x}^{\,\text{sim}}$ and the standard representation $R^{\,\text{std}}$ . . . 4
- 2.4 Maschke decompositions of $\vec{x}^{\,\text{ohe}}$ and $R^{\,\text{def}}$. . . 5
- 2.5 The tetrahedral embedding . . . 5

**3 Results for general groups** **6**
- 3.1 Nonzero embeddings . . . 6
- 3.2 Equivariance . . . 6
- 3.3 Irreducible embeddings are full rank . . . 6
- 3.4 Direct sums of inequivalent irreducible embeddings have full rank (Theorem 2 of Main Text) . . . 6

**4 Results for $S_\alpha$, the symmetric group** **7**
- 4.1 The trivial embedding $\vec{x}^{\,\text{triv}}$ and trivial representation $R^{\,\text{triv}}$ . . . 7
- 4.2 The simplex embedding $\vec{x}^{\,\text{sim}}$ and standard representation $R^{\,\text{std}}$ . . . 7
- 4.3 $\vec{x}^{\,\text{triv}}$ and $\vec{x}^{\,\text{sim}}$ are the only irreducible embeddings that transform under $S_\alpha$ . . . 8
- 4.4 Irreducible embeddings that co-transform under $S_\alpha$ are proportional . . . 9

**5 Results for $H_{\text{PSCP}}$, the group of position-specific character permutations** **10**
- 5.1 Irreducible embeddings that co-transform under $H_{\text{PSCP}}$ are proportional (Theorem 1 of Main Text) . . . 10
- 5.2 Generalized distillations of $H_{\text{PSCP}}$-equivariant embeddings . . . 11

**6 Results for specific models (Table 1 of Main Text)** **11**
- 6.1 $N$-order interaction model . . . 11
- 6.2 Hierarchical $N$-order interaction models . . . 12
  - 6.2.1 $N = 1$: Additive model . . . 12
  - 6.2.2 $N = 2$: Pairwise-interaction model . . . 13
  - 6.2.3 $N = L$: All-order interaction model . . . 13
- 6.3 $N$-adjacent interaction model . . . 13
- 6.4 Hierarchical $N$-adjacent interaction models . . . 15
  - 6.4.1 $N = 1$: Additive model . . . 15
  - 6.4.2 $N = 2$: Nearest-neighbor interaction model . . . 15
  - 6.4.3 $N = L$: All-adjacent interaction model . . . 16

**7 Results for other symmetry groups: $H_{\text{GCP}}$, $H_{\text{PP}}$, and $H_{\text{PSCP}}$ 16**
7.1 Distillation does not work for $H_{\text{GCP}}$ or $H_{\text{PP}}$ . . . 16
7.2 Distillation does work for $H_{\text{Ham}}$ . . . 17

**8 Distillation algorithm 18**
8.1 Overview of the algorithm . . . 18
8.2 General one-hot and simplex encodings . . . 19
8.3 Computation of the decomposition matrix $T_{\text{decom}}$. . . . 19
8.4 Computation of the thinning matrix $T_{\text{thin}}$ . . . 20
8.5 Computation of the sorting matrix $T_{\text{sort}}$ . . . 20

**9 Parameter interpretation 21**
9.1 Properties of allelic models . . . 21
9.2 One-hot models provide a lower bound on gauge freedoms . . . 21

**10 Restriction to the reals 22**

# 1 Preliminaries

## 1.1 General notation

- $\mathcal{U}$ denotes a finite set.
- $H$ denotes a group.
- $\vec{x}$, $\vec{y}$ denote complex embeddings.
- $\simeq$ denotes equivalence.
- $M$, $m$ denote the number of dimensions.
- $\gamma$ denotes the number of gauge freedoms.
- $R$ denotes a complex group representation.
- $\vec{\theta}$ denote model parameters.
- $J, K$ denote the number of elements in a direct sum.
- $L$ denotes the length of a sequence.
- $\alpha$ denotes the number of characters in alphabet $\mathcal{A}$.
- $f$ denotes a linear model of a sequence-function relationship.
- $i \leftrightarrow j$ denotes a transposition, i.e. a permutation that only exchanges $i$ and $j$.

## 1.2 Specific sets

- $\mathcal{I}_\alpha$ denotes $(1, 2, \ldots, \alpha)$, an ordered set comprising the first $\alpha$ positive integers.
- $\mathcal{A}$ denotes the alphabet $(c_1, \ldots, c_\alpha)$, an ordered set comprising $\alpha$ characters.
- $\mathcal{S}$ denotes sequence space, i.e., the set of sequences of length $L$ built from the $\alpha$ characters in $\mathcal{A}$.

## 1.3 Specific groups

- $S_\alpha$ denotes the symmetric group and acts on $\mathcal{I}_\alpha$.
- $H_{\text{CP}}$ denotes the group of character permutations and acts on $\mathcal{A}$.
- $H^l_{\text{CP}}$ denotes the group of single-position character permutations and acts on $\mathcal{S}$.
- $H_{\text{PSCP}}$ denotes the group of position-specific character permutations and acts on $\mathcal{S}$.
- $H_{\text{PP}}$ denotes the group of position permutations and acts on $\mathcal{S}$.
- $H_{\text{Ham}}$ denotes the group of Hamming graph symmetries and acts on $\mathcal{S}$.

## 1.4 Specific embeddings

- $\vec{x}^{\,\mathrm{triv}}$ denotes the trivial embedding of $\mathcal{I}_\alpha$, $\mathcal{A}$, or $\mathcal{S}$.
- $\vec{x}^{\,\mathrm{ohe}}$ denotes the one-hot embedding of $\mathcal{I}_\alpha$ or $\mathcal{A}$.
- $\vec{x}^{\,\mathrm{sim}}$ denotes the simplex embedding of $\mathcal{I}_\alpha$ or $\mathcal{A}$.
- $\vec{x}^{\,\mathrm{tet}}$ denotes the tetrahedral embedding of $\mathcal{A}$, applicable when $\alpha = 4$.
- $\vec{x}_l^{\,\mathrm{ohe}}$, $l \in \mathcal{I}_L$, denote a single-position one-hot embedding of $\mathcal{S}$.
- $\vec{x}_l^{\,\mathrm{sim}}$, $l \in \mathcal{I}_L$, denotes a single-position simplex embedding of $\mathcal{S}$.

## 1.5 Definitions

- **co-transformation**: Two equivariant embeddings *co-transform* if they are transformed by the same group representation.
- **embedding**: An *embedding* $\vec{x}$ is a map from a set $\mathcal{U}$ to a vector space $\mathbb{C}^m$; $m$ is called the *degree* of $\vec{x}$, denoted $\deg \vec{x}$.
- **equivariance**:
  - An embedding $\vec{x}$ of a set $\mathcal{U}$ is *equivariant* under a group $H$ iff there is a group representation $R$ such that $\vec{x}(hu) = R(h)\vec{x}(u)$ for all $h \in H$ and all $u \in \mathcal{U}$.
  - A linear model $f$ on a set $\mathcal{U}$ is *equivariant* under a group $H$ iff $f(u, \vec{\theta}) = \vec{\theta}^{\dagger}\vec{x}(u)$ for all $u \in H$, where $\vec{x}$ is an embedding of $\mathcal{U}$ that is equivariant under $H$, and $\vec{\theta} \in \mathbb{C}^m$ where $m = \deg \vec{x}$.
- **equivalence**:
  - Two embeddings $\vec{x}$ and $\vec{y}$ of a set $\mathcal{U}$ are *equivalent*, denoted $\vec{x} \simeq \vec{y}$, iff there is an invertible matrix $A$ (a similarity transformation) such that $\vec{y}(u) = A\vec{x}(u)$ for all $u \in \mathcal{U}$.
  - Two representations $R$ and $R'$ of a group $H$ are *equivalent*, denoted $R \simeq R'$, iff there is an invertible matrix $A$ (a similarity transformation) such that $R(h) = AR'(h)A^{-1}$ for all $h \in H$.
- **degree**:
  - The degree of an embedding $\vec{x}$, denoted $\deg \vec{x}$, is the number of elements in each embedding vector.
  - The degree of a representation $R$, denoted $\deg R$, is the number of rows (or equivalently the number of columns) of each matrix in the representation.
- **full rank embedding**: An embedding $\vec{x}$ of degree $m$ is *full rank* iff $\mathrm{span}\, \vec{x} = \mathbb{C}^m$.
- **irreducibility**: An *irreducible* embedding is an equivariant embedding that transforms under an irreducible representation.
- **nonzero embedding**: An embedding $\vec{x}$ of a set $\mathcal{U}$ is *nonzero* iff $\vec{x}(u) \neq 0$ for some $u \in \mathcal{U}$.
- **module**:
  - A *module* $V$ is defined by a vector space together with a group representation that transforms the elements of that vector space.
  - The *module* of an equivariant embedding $\vec{x}$ that has degree $m$ and transforms under the representation $R$ is the module defined by the vector space $\mathbb{C}^m$ and representation $R$.
- **representation**: A group representation $R$ is a map from a group $H$ to a set of matrices that preserve the multiplicaiton rules of $H$, i.e., for which $R(h_1)R(h_2) = R(h_1 h_2)$ for all $h_1, h_2 \in H$.
- **span of an embedding**: The *span* of an embedding $\vec{x}$ of a set $\mathcal{U}$, denoted $\mathrm{span}\, \vec{x}$, is the span of the set of vectors $\{\vec{x}(u) : u \in \mathcal{U}\}$.

# 2 Example: Embeddings and representations for single DNA characters

To concretely illustrate embeddings and representations, we now consider how a single DNA character is embedded and transforms under character permutations. We consider three embeddings of the DNA alphabet $\mathcal{A} = \{\mathrm{A}, \mathrm{C}, \mathrm{G}, \mathrm{T}\}$: the one-hot embedding $\vec{x}^{\,\mathrm{ohe}}$, the trivial embedding $\vec{x}^{\,\mathrm{triv}}$, and the simplex embedding $\vec{x}^{\,\mathrm{sim}}$. These embeddings are all equivariant under the group of character permutations $H_{\mathrm{CP}}$, therefore we also describe the representations of $H_{\mathrm{CP}}$ that these embeddings transform under: the defining representation $R^{\,\mathrm{def}}$, the trivial representation $R^{\,\mathrm{triv}}$, and the standard representation $R^{\,\mathrm{std}}$. Finally, we describe explicitly how $\vec{x}^{\,\mathrm{ohe}}$ decomposes into a direct sum of $\vec{x}^{\,\mathrm{triv}}$ and $\vec{x}^{\,\mathrm{sim}}$, and how $R^{\,\mathrm{def}}$ similarly decomposes into a direct sum of $R^{\,\mathrm{triv}}$ and $R^{\,\mathrm{std}}$.

## 2.1 The one-hot embedding $\vec{x}^{\,\mathrm{ohe}}$ and the defining representation $R^{\,\mathrm{def}}$

The one-hot encoding $\vec{x}^{\,\mathrm{ohe}}$ of characters in $\mathcal{A}$ has degree 4 and is given by

$$\vec{x}^{\,\mathrm{ohe}}(\mathrm{A}) = \begin{bmatrix} 1 \\ 0 \\ 0 \\ 0 \end{bmatrix}, \quad \vec{x}^{\,\mathrm{ohe}}(\mathrm{C}) = \begin{bmatrix} 0 \\ 1 \\ 0 \\ 0 \end{bmatrix}, \quad \vec{x}^{\,\mathrm{ohe}}(\mathrm{G}) = \begin{bmatrix} 0 \\ 0 \\ 1 \\ 0 \end{bmatrix}, \quad \vec{x}^{\,\mathrm{ohe}}(\mathrm{T}) = \begin{bmatrix} 0 \\ 0 \\ 0 \\ 1 \end{bmatrix}. \tag{1}$$

$\vec{x}^{\,\mathrm{ohe}}$ transforms under the defining representation, $R^{\,\mathrm{def}}$, of $H_{\mathrm{CP}}$. $H_{\mathrm{CP}}$ is generated by 6 transpositions, i.e. group elements that switch two characters while keeping all other characters fixed. We denote these six transpositions as $\mathrm{A} \leftrightarrow \mathrm{C}$, $\mathrm{A} \leftrightarrow \mathrm{G}$, $\mathrm{A} \leftrightarrow \mathrm{T}$, $\mathrm{C} \leftrightarrow \mathrm{G}$, $\mathrm{C} \leftrightarrow \mathrm{T}$, and $\mathrm{G} \leftrightarrow \mathrm{T}$. For these six generating transpositions, $R^{\,\mathrm{def}}$ is given by

$$R^{\,\mathrm{def}}(\mathrm{A} \leftrightarrow \mathrm{C}) = \begin{bmatrix} 0 & 1 & 0 & 0 \\ 1 & 0 & 0 & 0 \\ 0 & 0 & 1 & 0 \\ 0 & 0 & 0 & 1 \end{bmatrix}, \quad R^{\,\mathrm{def}}(\mathrm{A} \leftrightarrow \mathrm{G}) = \begin{bmatrix} 0 & 0 & 1 & 0 \\ 0 & 1 & 0 & 0 \\ 1 & 0 & 0 & 0 \\ 0 & 0 & 0 & 1 \end{bmatrix}, \quad R^{\,\mathrm{def}}(\mathrm{A} \leftrightarrow \mathrm{T}) = \begin{bmatrix} 0 & 0 & 0 & 1 \\ 0 & 1 & 0 & 0 \\ 0 & 0 & 1 & 0 \\ 1 & 0 & 0 & 0 \end{bmatrix}, \tag{2}$$

$$R^{\,\mathrm{def}}(\mathrm{C} \leftrightarrow \mathrm{G}) = \begin{bmatrix} 1 & 0 & 0 & 0 \\ 0 & 0 & 1 & 0 \\ 0 & 1 & 0 & 0 \\ 0 & 0 & 0 & 1 \end{bmatrix}, \quad R^{\,\mathrm{def}}(\mathrm{C} \leftrightarrow \mathrm{T}) = \begin{bmatrix} 1 & 0 & 0 & 0 \\ 0 & 0 & 0 & 1 \\ 0 & 0 & 1 & 0 \\ 0 & 1 & 0 & 0 \end{bmatrix}, \quad R^{\,\mathrm{def}}(\mathrm{G} \leftrightarrow \mathrm{T}) = \begin{bmatrix} 1 & 0 & 0 & 0 \\ 0 & 1 & 0 & 0 \\ 0 & 0 & 0 & 1 \\ 0 & 0 & 1 & 0 \end{bmatrix}. \tag{3}$$

## 2.2 The trivial embeddings $\vec{x}^{\,\mathrm{triv}}$ and the trivial representaiton $R^{\,\mathrm{triv}}$

The trivial embedding $\vec{x}^{\,\mathrm{triv}}$ of characters in $\mathcal{A}$ has degree 1 and is given by

$$\vec{x}^{\,\mathrm{triv}}(c) = [1] \quad \text{for all} \quad c \in \mathcal{A}. \tag{4}$$

$\vec{x}^{\,\mathrm{triv}}$ transforms under the trivial representation, $R^{\,\mathrm{triv}}$, of $H_{\mathrm{CP}}$. $R^{\,\mathrm{triv}}$ also has degree 1 and is given by

$$R^{\,\mathrm{triv}}(h) = [1] \quad \text{for all} \quad h \in H_{\mathrm{CP}}. \tag{5}$$

## 2.3 The simplex embedding $\vec{x}^{\,\mathrm{sim}}$ and the standard representation $R^{\,\mathrm{std}}$

The simplex embedding and the standard representation can be formulated in multiple equivalent ways. Here we present one realization that is particularly amenable to analytic calculations in arbitrary dimensions. In this realization, the simplex embedding $\vec{x}^{\,\mathrm{sim}}$ has degree 3 and is given by

$$\vec{x}^{\,\mathrm{sim}}(\mathrm{A}) = \begin{bmatrix} 1 \\ 0 \\ 0 \end{bmatrix}, \quad \vec{x}^{\,\mathrm{sim}}(\mathrm{C}) = \begin{bmatrix} 0 \\ 1 \\ 0 \end{bmatrix}, \quad \vec{x}^{\,\mathrm{sim}}(\mathrm{G}) = \begin{bmatrix} 0 \\ 0 \\ 1 \end{bmatrix}, \quad \vec{x}^{\,\mathrm{sim}}(\mathrm{T}) = \begin{bmatrix} -1 \\ -1 \\ -1 \end{bmatrix}. \tag{6}$$

$\vec{x}^{\,\mathrm{sim}}$ transforms under the standard representation, $R^{\,\mathrm{std}}$, of $H_{\mathrm{CP}}$. $R^{\,\mathrm{std}}$ also has degree 3, and for the 6 generating elements of $H_{\mathrm{CP}}$, it is given by

$$R^{\,\mathrm{std}}(\mathrm{A} \leftrightarrow \mathrm{C}) = \begin{bmatrix} 0 & 1 & 0 \\ 1 & 0 & 0 \\ 0 & 0 & 1 \end{bmatrix}, \quad R^{\,\mathrm{std}}(\mathrm{A} \leftrightarrow \mathrm{G}) = \begin{bmatrix} 0 & 0 & 1 \\ 0 & 1 & 0 \\ 1 & 0 & 0 \end{bmatrix}, \quad R^{\,\mathrm{std}}(\mathrm{C} \leftrightarrow \mathrm{G}) = \begin{bmatrix} 1 & 0 & 0 \\ 0 & 0 & 1 \\ 0 & 1 & 0 \end{bmatrix}, \tag{7}$$

$$R^{\,\mathrm{std}}(\mathrm{A} \leftrightarrow \mathrm{T}) = \begin{bmatrix} -1 & 0 & 0 \\ -1 & 1 & 0 \\ -1 & 0 & 1 \end{bmatrix}, \quad R^{\,\mathrm{std}}(\mathrm{C} \leftrightarrow \mathrm{T}) = \begin{bmatrix} 1 & -1 & 0 \\ 0 & -1 & 0 \\ 0 & -1 & 1 \end{bmatrix}, \quad R^{\,\mathrm{std}}(\mathrm{G} \leftrightarrow \mathrm{T}) = \begin{bmatrix} 1 & 0 & -1 \\ 0 & 1 & -1 \\ 0 & 0 & -1 \end{bmatrix}. \tag{8}$$

### 2.4 Maschke decompositions of $\vec{x}^{\,\text{ohe}}$ and $R^{\,\text{def}}$.

The defining representation decomposes, by Maschke's Theorem, into the direct sum of the trivial representation and the standard representation:

$$R^{\,\text{def}} \simeq R^{\,\text{triv}} \oplus R^{\,\text{std}}. \tag{9}$$

Similarly, the one-hot encoding decomposes into the direct sum of the trivial embedding and the simplex embedding:

$$\vec{x}^{\,\text{ohe}} \simeq \vec{x}^{\,\text{triv}} \oplus \vec{x}^{\,\text{sim}}. \tag{10}$$

These decompositions are accomplished by the similarity transformation matrix $T$, in the sense that

$$T^{(1)}_{\text{decom}} R^{\,\text{def}} \left(T^{(1)}_{\text{decom}}\right)^{-1} = \left[\begin{array}{c|ccc} R^{\,\text{triv}} & 0 & 0 & 0 \\ \hline 0 & & & \\ 0 & & R^{\,\text{std}} & \\ 0 & & & \end{array}\right], \quad T^{(1)}_{\text{decom}} \vec{x}^{\,\text{ohe}} = \left[\begin{array}{c} \vec{x}^{\,\text{triv}} \\ \hline \\ \vec{x}^{\,\text{sim}} \\ \\ \end{array}\right], \tag{11}$$

where

$$T^{(1)}_{\text{decom}} = \begin{bmatrix} 1 & 1 & 1 & 1 \\ 1 & 0 & 0 & -1 \\ 0 & 1 & 0 & -1 \\ 0 & 0 & 1 & -1 \end{bmatrix} \quad \Rightarrow \quad \left(T^{(1)}_{\text{decom}}\right)^{-1} = \frac{1}{4}\begin{bmatrix} 1 & 3 & -1 & -1 \\ 1 & -1 & 3 & -1 \\ 1 & -1 & -1 & 3 \\ 1 & -1 & -1 & -1 \end{bmatrix} \tag{12}$$

### 2.5 The tetrahedral embedding

It is worth noting that, instead of the simplex embedding for DNA characters, some studies (e.g., [3]) have what we call the tetrahedral embedding, $\vec{x}^{\,\text{tet}}$. The tetrahedral embedding is given by

$$\vec{x}^{\,\text{tet}}(\text{A}) = \begin{bmatrix} 1 \\ 1 \\ 1 \end{bmatrix}, \quad \vec{x}^{\,\text{tet}}(\text{C}) = \begin{bmatrix} 1 \\ -1 \\ -1 \end{bmatrix}, \quad \vec{x}^{\,\text{tet}}(\text{G}) = \begin{bmatrix} -1 \\ 1 \\ -1 \end{bmatrix}, \quad \vec{x}^{\,\text{tet}}(\text{T}) = \begin{bmatrix} -1 \\ -1 \\ 1 \end{bmatrix}. \tag{13}$$

$\vec{x}^{\,\text{tet}}$ transforms under a corresponding tetrahedral representation, $R^{\,\text{tet}}$. In the tetrahedral representation, the 6 generating elements of $H_{\text{CP}}$ are given by

$$R^{\,\text{tet}}(\text{A}\leftrightarrow\text{C}) = \begin{bmatrix} 1 & 0 & 0 \\ 0 & 0 & -1 \\ 0 & -1 & 0 \end{bmatrix}, \quad R^{\,\text{tet}}(\text{A}\leftrightarrow\text{G}) = \begin{bmatrix} 0 & 0 & -1 \\ 0 & 1 & 0 \\ -1 & 0 & 0 \end{bmatrix}, \quad R^{\,\text{tet}}(\text{A}\leftrightarrow\text{T}) = \begin{bmatrix} 0 & -1 & 0 \\ -1 & 0 & 0 \\ 0 & 0 & 1 \end{bmatrix}, \tag{14}$$

$$R^{\,\text{tet}}(\text{C}\leftrightarrow\text{G}) = \begin{bmatrix} 0 & 1 & 0 \\ 1 & 0 & 0 \\ 0 & 0 & 1 \end{bmatrix}, \quad R^{\,\text{tet}}(\text{C}\leftrightarrow\text{T}) = \begin{bmatrix} 0 & 0 & 1 \\ 0 & 1 & 0 \\ 1 & 0 & 0 \end{bmatrix}, \quad R^{\,\text{tet}}(\text{G}\leftrightarrow\text{T}) = \begin{bmatrix} 1 & 0 & 0 \\ 0 & 0 & 1 \\ 0 & 1 & 0 \end{bmatrix}. \tag{15}$$

The tetrahedral embedding $\vec{x}^{\,\text{tet}}$ is equivalent to the simplex embedding $\vec{x}^{\,\text{sim}}$, and the tetrahedral representation $R^{\,\text{tet}}$ is equivalent to the standard representation $R^{\,\text{std}}$. The Maschke decomposition formulas of $R^{\,\text{def}}$ and $\vec{x}^{\,\text{ohe}}$ in Sec. 2.4, using $R^{\,\text{tet}}$ and $\vec{x}^{\,\text{tet}}$ in place of $R^{\,\text{std}}$ and $\vec{x}^{\,\text{sim}}$, are given by,

$$R^{\,\text{def}} = T \left[\begin{array}{c|ccc} R^{\,\text{triv}} & 0 & 0 & 0 \\ \hline 0 & & & \\ 0 & & R^{\,\text{tet}} & \\ 0 & & & \end{array}\right] T^{-1} \quad \text{and} \quad \vec{x}^{\,\text{ohe}} = T \left[\begin{array}{c} \vec{x}^{\,\text{triv}} \\ \hline \\ \vec{x}^{\,\text{tet}} \\ \\ \end{array}\right], \tag{16}$$

where

$$T = \frac{1}{4}\begin{bmatrix} 1 & 1 & 1 & 1 \\ 1 & 1 & -1 & -1 \\ 1 & -1 & 1 & -1 \\ 1 & -1 & -1 & 1 \end{bmatrix} \quad \Rightarrow \quad T^{-1} = \begin{bmatrix} 1 & 1 & 1 & 1 \\ 1 & 1 & -1 & -1 \\ 1 & -1 & 1 & -1 \\ 1 & -1 & -1 & 1 \end{bmatrix}. \tag{17}$$

Note that $T^{-1}$ is a Haddamard matrix of order 4 [3]. The simplex embedding has an advantage over the tetrahedral embedding, however, in that it is unclear how to generalize the tetrahedral embedding to arbitrary numbers of characters $\alpha$.

# 3 Results for general groups

## 3.1 Nonzero embeddings

**Proposition 1.** *Let $\vec{x}$ be an embedding of a set $\mathcal{U}$ that transforms under a representation of a group $H$ that is transitive on $\mathcal{U}$. Then either $\vec{x}(u) = \vec{0}$ all $u \in \mathcal{U}$, or $\vec{x}(u) \neq \vec{0}$ for all $u \in \mathcal{U}$.*

*Proof.* Assume that $\vec{x}(u) = \vec{0}$ for some $u \in \mathcal{U}$, and choose any other $u' \in \mathcal{U}$. Because $H$ is transitive, there is a group element $h \in \mathcal{H}$ such that $u' = hu$. Letting $R$ denote the representation of $H$ that $\vec{x}$ transforms under, we find

$$\vec{x}(u') = R(h)\vec{x}(u) = R(h)\vec{0} = \vec{0}. \tag{18}$$

We thus find that $\vec{x}(u) = \vec{0}$ for some $u \in \mathcal{U}$ implies that $\vec{x}(u) = \vec{0}$ for all $u \in \mathcal{U}$. This completes the proof.

We note, however, that if $H$ is not transitive on $\mathcal{U}$, then Prop. 1 will generally not hold. □

## 3.2 Equivariance

**Proposition 2.** *If $f$ is a linear model based on the embedding $\vec{x}$ of a set $\mathcal{U}$ into a vector space $V$, and $\vec{x}$ is an equivariant embedding that transforms under a representation $R$ of a group $H$, then*

$$f\left(u; \vec{\theta}\,\right) = f\left(hu; R(h)^{-1\dagger}\,\vec{\theta}\,\right) \tag{19}$$

*for all $\vec{\theta} \in V$, all $u \in \mathcal{U}$, and all $h \in H$.*

*Proof.*

$$\begin{aligned} f(hu; R(h)^{-1\dagger}\vec{\theta}) &= [R(h)^{-1\dagger}\vec{\theta}]^\dagger \vec{x}(hu) && (20)\\ &= \vec{\theta}^\dagger R(h)^{-1} R(h)\vec{x}(u) && (21)\\ &= \vec{\theta}^\dagger \vec{x}(u) && (22)\\ &= f(u; \vec{\theta}). && (23) \end{aligned}$$

□

## 3.3 Irreducible embeddings are full rank

**Proposition 3.** *Every nonzero equivariant embedding that transforms under an irreducible representation is full rank.*

*Proof.* Let $\vec{x}$ be a nonzero embedding of a set $\mathcal{U}$ into a vector space $V$. Further assume that $\vec{x}$ transforms under an irreducible representation $R$ of a group $H$. Since $R$ transforms vectors in $V$, $V$ is an irreducible $H$ module. For any $h \in H$,

$$\begin{aligned} R(h)\,\mathrm{span}\,\vec{x} &= \mathrm{span}\,\{R(h)\vec{x}(u) : u \in \mathcal{U}\} && (24)\\ &= \mathrm{span}\,\{\vec{x}(hu) : u \in \mathcal{U}\} && (25)\\ &= \mathrm{span}\,\{\vec{x}(u) : u \in \mathcal{U}\} && (26)\\ &= \mathrm{span}\,\vec{x}. && (27) \end{aligned}$$

Therefore, $\mathrm{span}\,\vec{x}$ is an $H$-invariant subspace of $V$. Since $V$ is an irreducible module, the only $H$-invariant subspaces of $V$ are 0 and $V$. Because $\vec{x}$ is nonzero, $\mathrm{span}\,\vec{x} \neq 0$. We thus find that $\mathrm{span}\,\vec{x} = V$, proving that $\vec{x}$ is full rank. □

## 3.4 Direct sums of inequivalent irreducible embeddings have full rank (Theorem 2 of Main Text)

**Proposition 4.** *(Theorem 2 of Main Text) For each $k \in \{1, \ldots, K\}$, let $\vec{x}_k$ be a nonzero embedding that transforms under an irreducible representation $R_k$ of a group $H$. Then the direct sum of all $\vec{x}_k$ is full rank if all $R_k$ are pairwise inequivalent.*

*Proof.* Let $V = \bigoplus_{k=1}^K V_k$ where $V_k$ is the vector space to which the embedding $\vec{x}_k$ maps. Let $R = \bigoplus_{k=1}^K R_k$, we see that $R$ is a representation of $H$ over $V$.

Given $R$, define an $H$-invariant inner product $\langle \cdot, \cdot \rangle$ over $V$; note that this can always be accomplished (e.g., see p. 16 of [2]). Next, using the notation $\vec{x}(s) = \bigoplus_{k=1}^K \vec{x}_k(s)$, define,

$$W = \{\vec{w} \in V : \langle \vec{w}, \vec{x}(s) \rangle = 0 \;\text{ for all }\; s \in \mathcal{S}\}\,. \tag{28}$$

$W$ is $H$-invariant, since for all $\vec{w} \in W$ and $h \in H_{\text{PSCP}}$,

$$\langle R(h)\vec{w}, \vec{x}(s)\rangle = \langle \vec{w}, R(h)^{-1}\vec{x}(s)\rangle = \langle \vec{w}, \vec{x}(h^{-1}s)\rangle = 0. \tag{29}$$

We are goingto show that $\dim(W) = 0$.

We start by showing that the projection operator $P$ that projects $V$ orthogonally on to $W$ according to the inner product $\langle \cdot, \cdot \rangle$ is an $H$-homomorphism. Assume $W$ has dimension $n$, and let $\{\hat{e}_1, \dots, \hat{e}_n\}$ be an orthonormal basis of $W$ (i.e., $\langle \hat{e}_i, \hat{e}_j \rangle = \delta_{ij}$ for all $i, j \in \mathcal{I}_n$). We define the projection operator $P$ to be such that

$$P\vec{v} = \sum_{i=1}^{n} \hat{e}_i \langle \hat{e}_i, \vec{v}\rangle \quad \text{for all} \quad v \in V. \tag{30}$$

Because $\langle \cdot, \cdot \rangle$ is a $H$-invariant inner product, $\langle R(h)\hat{e}_i, R(h)\hat{e}_j \rangle = \delta_{ij}$ for all $h \in H$, and thus $\{R(h)\hat{e}_i\}_{i=1}^n$ is also an orthonormal basis for $W$. Consequently, for all $h \in H$ and $\vec{v} \in V$,

$$P\vec{v} = \sum_{i=1}^{n} R(h)\hat{e}_i \langle R(h)\hat{e}_i, \vec{v}\rangle = R(h)\sum_{i=1}^{n} \hat{e}_i \langle \hat{e}_i, R(h)^{-1}\vec{v}\rangle = R(h)PR(h)^{-1}\vec{v}. \tag{31}$$

We therefore see that $P$ commutes with $R$, so it is an $H$-homomorphism.

By theorem 1.7.8 in [2], there is a set of complex scalars $\{c_k\}_{k=1}^K$ such that

$$P = \bigoplus_{k=1}^{K} c_k I_k, \tag{32}$$

where $I_k$ denotes the identity operator on $V_k$. Because $P\vec{x}(s) = \vec{0}$ for all $s \in \mathcal{S}$,

$$0 = \langle P\vec{x}(s), P\vec{x}(s)\rangle = \sum_{k=1}^{K} \langle c_k\vec{x}_k(s), c_k\vec{x}_k(s)\rangle = \sum_{k=1}^{K} ||c_k||^2 ||\vec{x}_k(s)||^2. \tag{33}$$

Since all $\vec{x}_k$ are nonzero, this is only possible if all $c_k = 0$. We thus find that $P$ projects all vectors to $\vec{0}$, and hence that $W$ has dimension 0. Since $W$ is the orthogonal complement of $\operatorname{span}\vec{x}$, $\vec{x}$ has full rank. This completes the proof. □

# 4 Results for $S_\alpha$, the symmetric group

The symmetric group, $S_\alpha$, is the group of permutations among the integers $\{1, \dots, \alpha\} = \mathcal{I}_\alpha$. $S_\alpha$ is generated by transpositions, which exchange two elements of $\mathcal{I}_\alpha$ while leaving all other elements unchanged. In what follows, we use $i \leftrightarrow j$ to denote the transposition that exchanges elements $i < j \in \mathcal{I}_\alpha$.

## 4.1 The trivial embedding $\vec{x}^{\,\text{triv}}$ and trivial representation $R^{\text{triv}}$

The trivial embedding of $\mathcal{I}_\alpha$, $\vec{x}^{\,\text{triv}}$, has degree 1 and is given by

$$\vec{x}^{\,\text{triv}}(i) = [1] \quad \text{for all} \quad i \in I_\alpha. \tag{34}$$

$\vec{x}^{\,\text{triv}}$ transforms under the trivial representation, $R^{\text{triv}}$, which also has degree 1 and is given by

$$R^{\text{triv}}(h) = [1] \quad \text{for all} \quad h \in S_\alpha. \tag{35}$$

## 4.2 The simplex embedding $\vec{x}^{\,\text{sim}}$ and standard representation $R^{\text{std}}$

Here we define an explicit coordinate realization of the simplex embedding $\vec{x}^{\,\text{sim}}$ of $\mathcal{I}_\alpha$, as well as the standard representation $R^{\text{std}}$ of $S_\alpha$, under which $\vec{x}^{\,\text{sim}}$ transforms. Both $\vec{x}^{\,\text{sim}}$ and $R^{\text{std}}$ have degree $\alpha - 1$, and we use $k, l \in \mathcal{I}_{\alpha-1}$ to index the elements of $\vec{x}^{\,\text{sim}}$ and $R^{\text{std}}$. We define the coordinates of $\vec{x}^{\,\text{sim}}$ to be

$$[\vec{x}^{\,\text{sim}}(i)]_k = \delta_{ik} \quad \text{for} \quad i \in \mathcal{I}_{\alpha-1}, \tag{36}$$

$$[\vec{x}^{\,\text{sim}}(\alpha)]_k = -1. \tag{37}$$

Correspondingly, we define the coordinate realization of $R^{\text{std}}$ on transpositions to be

$$[R^{\text{std}}(i \leftrightarrow j)]_{kl} = \delta_{kl} - \delta_{ik}\delta_{il} - \delta_{jk}\delta_{jl} + \delta_{ik}\delta_{jl} + \delta_{jk}\delta_{il}, \tag{38}$$

$$[R^{\text{std}}(i \leftrightarrow \alpha)]_{kl} = \delta_{kl} - \delta_{kl}\delta_{ik} - \delta_{il}. \tag{39}$$

One can readily verify that these definitions work by showing that $\vec{x}^{\,\text{sim}}$ does in fact transform according to $R^{\,\text{std}}$ for all transpositions in $S_\alpha$, thereby showing that $R^{\,\text{std}}$ is indeed a valid representation of $S_\alpha$. The embedding and representation in Sec. 2.3 are an example of this realization for $\alpha = 4$. Finally, we note that $\vec{x}^{\,\text{std}}$ has the useful property that

$$\sum_{i=1}^{\alpha} \vec{x}^{\,\text{sim}}(i) = \vec{0}. \tag{40}$$

### 4.3 $\vec{x}^{\,\text{triv}}$ and $\vec{x}^{\,\text{sim}}$ are the only irreducible embeddings that transform under $S_\alpha$

**Proposition 5.** *If a nonzero embedding of $\mathcal{I}_\alpha$ transforms under an irreducible representation of $S_\alpha$, then it is equivalent to either the trivial embedding or the simplex embedding.*

*Proof.* Assume that $\vec{x}$ is a nonzero embedding of $\mathcal{I}_\alpha$, that $\vec{x}$ has degree $m$, and that $\vec{x}$ transforms under an irreducible representation $R$ of $\mathcal{S}_\alpha$.

First consider $\alpha = 2$. $S_2$ has only two inequivalent irreducible representations: the trivial representation and the standard representation. The trivial representation supports the embedding $\vec{x}^{\,\text{triv}}$, and the standard representation supports the simplex embedding $\vec{x}^{\,\text{sim}}$. This proves the proposition for $\alpha = 2$.

Now consider $\alpha \geq 3$. Select $i, j, k \in \mathcal{I}_\alpha$, where $i \neq j$, $i \neq k$, $j \neq k$, and assume that $\vec{x}(i) = \vec{x}(j) \neq \vec{x}(k)$. The transposition $j \leftrightarrow k$ exchanges $j$ and $k$ without affecting $i$. Therefore, the representation of $R(j \leftrightarrow k)$ must exchange the vectors $\vec{x}(j)$ and $\vec{x}(k)$ while leaving the vector $\vec{x}(i)$ invariant. But this is impossible because $\vec{x}(i) = \vec{x}(j)$. Consequently, $\vec{x}(i)$ must be either the same for all $i \in \mathcal{I}_\alpha$, or must be different for all $i \in \mathcal{I}_\alpha$. If $\vec{x}(i)$ is the same for all $i$, then $\vec{x} \simeq \vec{x}^{\,\text{triv}}$. In the rest of this proof we assume that $\vec{x}(i)$ is different for all $i \in \mathcal{I}_\alpha$. We will show that this implies $\vec{x} \simeq \vec{x}^{\,\text{sim}}$.

Prop. 3 shows that, because $\vec{x}$ is irreducible, $\vec{x}$ is also full rank. We can therefore choose a subset $\{n_1, \ldots, n_m\} \subseteq \mathcal{I}_\alpha$ such that $\{\vec{x}(n_1), \ldots, \vec{x}(n_m)\}$ is a basis for $\mathbb{C}^m$. For simplicity, we assume that $n_i = i$ for all $i \in \mathcal{I}_m$, so that $\mathcal{B} = \{\vec{x}(i) : i \in \mathcal{I}_m\}$ is the basis; we revisit this assumption at the end of the proof. In the basis $\mathcal{B}$, the elements of $\vec{x}$ are

$$[\vec{x}(i)]_k = \delta_{ik} \tag{41}$$

for all $i, k \in \mathcal{I}_m$.

Now choose $a, b, j \in \mathcal{I}_m$ such that $a \neq b$, $a \neq j$, $b \neq j$. We can compute the elements of $R(a \leftrightarrow b)$ in basis $\mathcal{B}$ by noting that

$$[\vec{x}(a)]_i = \sum_{k=1}^{m} [R(a \leftrightarrow b)]_{ik} [\vec{x}(b)]_k = [R(a \leftrightarrow b)]_{ib}, \tag{42}$$

$$[\vec{x}(b)]_i = \sum_{k=1}^{m} [R(a \leftrightarrow b)]_{ik} [\vec{x}(a)]_k = [R(a \leftrightarrow b)]_{ia}, \tag{43}$$

$$[\vec{x}(j)]_i = \sum_{k=1}^{m} [R(a \leftrightarrow b)]_{ik} [\vec{x}(j)]_k = [R(a \leftrightarrow b)]_{ij} \quad \text{for} \quad j \neq a, b. \tag{44}$$

and therefore that,

$$[R(a \leftrightarrow b)]_{ia} = \delta_{ib}, \tag{45}$$

$$[R(a \leftrightarrow b)]_{ib} = \delta_{ia}, \tag{46}$$

$$[R(a \leftrightarrow b)]_{ij} = \delta_{ij} \quad \text{for} \quad j \neq a, b. \tag{47}$$

Combining Eq. 45, Eq. 46, and Eq. 47 into a single expression gives that, for any $a, b \in I_m$ such that $a \neq b$, and any $i, j \in I_m$,

$$[R(a \leftrightarrow b)]_{ij} = \delta_{ij}(1 - \delta_{aj})(1 - \delta_{bj}) + \delta_{aj}\delta_{bi} + \delta_{bj}\delta_{ai}, \tag{48}$$

$$= \delta_{ij} - \delta_{ai}\delta_{aj} - \delta_{bi}\delta_{bj} + \delta_{ai}\delta_{bj} + \delta_{bi}\delta_{aj}. \tag{49}$$

Now choose $c \in \mathcal{I}_\alpha \setminus \mathcal{I}_m$ and $a, j \in \mathcal{I}_m$ such that $a \neq j$. Because $\mathcal{B}$ is a basis, there are coefficients $r_i^c$, $i \in \mathcal{I}_m$, such that

$$\vec{x}(c) = \sum_{i=1}^{m} r_i^c \vec{x}(i) \tag{50}$$

Consequently, the elements of $\vec{x}(c)$ in the basis $\mathcal{B}$ are,

$$[\vec{x}(c)]_i = r_i^c. \tag{51}$$

We compute the matrix elements of $R(a \leftrightarrow c)$ by noting that

$$
\begin{aligned}
[\vec{x}(a)]_i &= \sum_{k=1}^{m} [R(a \leftrightarrow c)]_{ik} [\vec{x}(c)]_k = \sum_{k=1}^{m} [R(a \leftrightarrow c)]_{ik} r_k^c, && (52)\\
[\vec{x}(c)]_i &= \sum_{k=1}^{m} [R(a \leftrightarrow c)]_{ik} [\vec{x}(a)]_k = [R(a \leftrightarrow c)]_{ia}, && (53)\\
[\vec{x}(j)]_i &= \sum_{k=1}^{m} [R(a \leftrightarrow c)]_{ik} [\vec{x}(j)]_k = [R(a \leftrightarrow c)]_{ij}. && (54)
\end{aligned}
$$

From Eq. 41, Eq. 51, Eq. 53, and Eq. 54 we see that, for all $i, k \in \mathcal{I}_m$,

$$[R(a \leftrightarrow c)]_{ik} = \delta_{ik}(1 - \delta_{ak}) + r_i^c \delta_{ak} \tag{55}$$

are the elements of $R(a \leftrightarrow c)$. Plugging this into Eq. 52 then gives a constraint on the $r_i^c$ coefficients:

$$\delta_{ia} = \sum_{k=1}^{m} [\delta_{ik}(1 - \delta_{ak}) + r_i^c \delta_{ak}] r_k^c = r_i^c (1 - \delta_{ai}) + r_a^c r_i^c. \tag{56}$$

Considering different values for $i \in \mathcal{I}_\alpha$ in Eq. 56 then gives two specific constraints on $r_i^c$:

$$(r_a^c)^2 = 1 \text{ for } i = a \quad \text{and} \quad r_i^c(1 + r_a^c) = 0 \text{ for } i \neq a. \tag{57}$$

The left side of Eq. 57 gives $r_i^c = \pm 1$. If $r_a^c = 1$, the right side of Eq. 57 gives $r_i^c = 0$ for $i \neq a$. But this would mean that $\vec{x}(c) = \vec{x}(a)$, which violates the requirement that all $\vec{x}(i)$ are distinct. We thus find that $r_i^c = -1$. Therefore, for all $i, k \in \mathcal{I}_m$,

$$[\vec{x}(c)]_i = -1, \quad \text{and} \quad [R(a \leftrightarrow c)]_{ik} = \delta_{ik}(1 - \delta_{ak}) - \delta_{ak}. \tag{58}$$

In particular, this means that there is only a single possible $\vec{x}(c)$ for all $c \in \mathcal{I}_\alpha \setminus \mathcal{I}_m$. Since all $\vec{x}(c)$ must be distinct, there can only be one possible value for $c \geq m$, i.e. $c = \alpha = m + 1$. We therefore find that for $i, j, k, l \in I_{\alpha-1}$, $i \neq j$,

$$
\begin{aligned}
[\vec{x}(i)]_k &= \delta_{ik} && (59)\\
[\vec{x}(\alpha)]_k &= -1 && (60)\\
[R(i \leftrightarrow j)]_{kl} &= \delta_{kl} - \delta_{ik}\delta_{il} - \delta_{jk}\delta_{jl} + \delta_{ik}\delta_{jl} + \delta_{jk}\delta_{il}. && (61)\\
[R(i \leftrightarrow \alpha)]_{kl} &= \delta_{kl} - \delta_{ik}\delta_{kl} - \delta_{il} && (62)\\
& && (63)
\end{aligned}
$$

We therefore see that $\vec{x} = \vec{x}^{\,\text{sim}}$ and $R = R^{\text{std}}$. Finally, we revisit our simplifying assumption that $n_i = i$ for all $i \in \mathcal{I}_m$. It is readily seen that removing this assumption leads to

$$\vec{x} = T\vec{x}^{\,\text{sim}} \quad \text{and} \quad R = TR^{\text{std}}T^{-1} \tag{64}$$

where $T$ is the similarity transformation that maps each $\vec{x}(i)$ to $\vec{x}(n_i)$, i.e.,

$$T = R(g) \quad \text{where} \quad g = \begin{pmatrix} 1 & 2 & \cdots & m \\ n_1 & n_2 & \cdots & n_m \end{pmatrix}. \tag{65}$$

Consequently, $\vec{x} \simeq \vec{x}^{\,\text{sim}}$ and $R \simeq R^{\text{sim}}$. This completes the proof. □

## 4.4 Irreducible embeddings that co-transform under $S_\alpha$ are proportional

**Proposition 6.** *Nonzero embeddings that transform under the same irreducible representation of the symmetric group are equal up to a constant of proportionality.*

*Proof.* Let $\vec{x}$ and $\vec{y}$ be two embeddings of $\mathcal{I}_\alpha$ that transform according to the same irreducible representation $R$ of $S_\alpha$. First we construct a matrix $A$ such that

$$\vec{y}(i) = A\vec{x}(i) \quad \text{for all} \quad i \in \mathcal{I}_\alpha. \tag{66}$$

From Proposition 5, $\vec{x}$ and $\vec{y}$ must transform under the trivial representation or a representation equivalent to the standard representation. If $\vec{x}$ and $\vec{y}$ transform under the trivial representation, then both $\vec{x}(i)$ and $\vec{y}(i)$ are nonzero scalars that do

not depend on $i$, and Eq. 66 is satisfied by setting $A = \vec{y}/\vec{x}$. If $\vec{x}$ and $\vec{y}$ transform under a representation equivalent to the standard representation, then both $\vec{x}$ and $\vec{y}$ have degree $m = \alpha - 1$. Define the $m \times m$ matrices $X = [\vec{x}(1)\ \vec{x}(2)\ \ldots\ \vec{x}(m)]$ and $Y = [\vec{y}(1)\ \vec{y}(2)\ \ldots\ \vec{y}(m)]$. Next define $A = YX^{-1}$; note that $X^{-1}$ exists because the columns of $X$ are linearly independent. By construction, $\vec{y}(i) = A\vec{x}(i)$ for $i \in I_{\alpha-1}$. Moreover $\sum_{i=1}^{\alpha} \vec{x}(i) = \sum_{i=1}^{\alpha} \vec{y}(i) = \vec{0}$ from Eq. 40. Therefore,

$$\vec{y}(\alpha) = -\sum_{i=1}^{\alpha-1} \vec{y}(i) = -\sum_{i=1}^{\alpha-1} A\vec{x}(i) = A\vec{x}(\alpha) \tag{67}$$

as well. This proves that Eq. 66 is satisfied when $\vec{x}$ and $\vec{y}$ transform under the standard representation.

Next we show that $A$ commutes with $R$. For all $h \in \mathcal{S}_\alpha$ and all $i \in \mathcal{I}_\alpha$,

$$AR(h)\vec{x}(i) = A\vec{x}(hi) = \vec{y}(hi) = R(h)\vec{y}(i) = R(h)A\vec{x}(i). \tag{68}$$

Because $\vec{x}$ has full rank, this requires

$$AR(h) = R(h)A \tag{69}$$

for all $h \in S_\alpha$, thereby showing that $A$ and $R$ commute.

Finally, we show that $A = cI$ for some $c \in \mathbb{C}$. This is a consequence of Shur's Lemma (e.g., see Corollary 1.6.6 of ref. [2]), which tells us that any matrix that commutes with an irreducible representation is either invertible or the zero matrix. Now define $B = A - cI$ where $c$ is an eigenvalue of $A$. $B$ commutes with $R$, since

$$BR(h) = (A - cI)R(h) = R(h)(A - cI) = R(h)B. \tag{70}$$

But $B$ cannot be invertible because at least one of its eigenvalues is zero. $B$ must therefore be the zero matrix, implying that $A = cI$. This shows that $\vec{y} = c\vec{x}$, completing the proof. □

# 5 Results for $H_{\text{PSCP}}$, the group of position-specific character permutations

## 5.1 Irreducible embeddings that co-transform under $H_{\text{PSCP}}$ are proportional (Theorem 1 of Main Text)

**Proposition 7.** *(Theorem 1 of Main Text) Nonzero sequence embeddings that transform under the same irreducible representation of $H_{\text{PSCP}}$ are equal up to a constant of proportionality.*

*Proof.* As stated in Main Text, the group of position-specific character permutations, $H_{\text{PSCP}}$, is given by the direct product

$$H_{\text{PSCP}} = \bigotimes_{l=1}^{L} H_{\text{CP}}^{l}, \tag{71}$$

where $H_{\text{CP}}^{l}$ is the group of permutations among the $\alpha$ possible characters at position $l$ in a sequence. Therefore, every element $h \in H_{\text{PSCP}}$ can be expressed as the direct product

$$h = \bigotimes_{l=1}^{L} h_l, \quad h_l \in H_{\text{CP}}^{l}. \tag{72}$$

It is known that if we consider representations over an algebraically closed field like $\mathbb{C}$, then all irreducible representations of a product group can be expressed as tensor products of the irreducible representations of the individual group factors (e.g., Theorem 1.11.3 of ref. [2]). Therefore, any irreducible representation $R$ of $H_{\text{PSCP}}$ can be expressed as

$$R(h) = \bigotimes_{l=1}^{L} R_l(h_l), \tag{73}$$

where each $R_l$ is an irreducible representation of $H_{\text{CP}}^{l}$, and where, for each $h \in H_{\text{PSCP}}$, $h_l \in H_{\text{CP}}^{l}$ is the corresponding factor in Eq. 72.

Now let $\{\vec{x}_q\}_{q=1}^{Q}$ denote a set of embeddings that transform under $R$. Eq. 73 implies that each $\vec{x}_q$ must be expressible as a tensor product of site-specific embeddings as

$$\vec{x}_q = \bigotimes_{l=1}^{L} \vec{x}_q^{\,l}, \tag{74}$$

where each $\vec{x}_q^l$ is a site-specific embedding that transforms under $H_{\text{CP}}^l$. Because $H_{\text{CP}}^l$ is isomorphic to $S_\alpha$, each $R_l$ can be thought of as a representation of $\mathcal{S}_\alpha$, and each $\vec{x}_q^l$ can be thought of as an embedding of $\mathcal{I}_\alpha$. Proposition 6 says that any two nonzero embeddings of $\mathcal{I}_\alpha$ that transform under the same irreducible representation $S_\alpha$ must be scalar multiples of one another. There must therefore exist, for every $l \in \mathcal{I}_L$, a nonzero embedding $\vec{x}^l$ that transforms under $R_l$ and that satisfies

$$\vec{x}_q^l = c_q^l \vec{x}^l \tag{75}$$

for all $q \in \mathcal{I}_Q$. We therefore find that

$$\vec{x}_q = c_q \vec{x}, \quad \text{where} \quad c_q = \prod_{l=1}^{L} c_q^l \quad \text{and} \quad \vec{x} = \bigotimes_{l=1}^{L} \vec{x}^l. \tag{76}$$

This completes the proof. □

## 5.2 Generalized distillations of $H_{\text{PSCP}}$-equivariant embeddings

Here we remove the assumption in the Main Text that every irreducible embedding $\vec{x}_{kq}$ in Eq. 9 is nonzero. Removing this assumption and retracing the logic, it is straight-forward to see that only two changes to the main text equations are required. First, Eq. 12 of Main Text becomes

$$\vec{x}^{\,\text{dist}} = \bigoplus_{k \in \mathcal{K}} \vec{x}_k, \tag{77}$$

where $\mathcal{K} \subseteq \mathcal{I}_K$ is the set of $k$ such that $\vec{x}_{qk} \neq \vec{0}$ for at least one value of $q$, and where $\vec{x}_k$ is chosen to be any one of the nonzero embeddings $\vec{x}_{qk}$. Second, the statement that the number of gauge freedoms equals the sum of the degrees of all redundant irreducible representations still holds, but the term "redundant" deserves clarification. For every $k \in \mathcal{I}_K$, there are $Q_k$ copies of the representation $R_k$ in the Maschke decomposition (Eq. 8 of Main Text). When $k \in \mathcal{K}$, $Q_k - 1$ of these copies of $R_k$ are redundant, because all but one of the $Q_k$ embeddings $\vec{x}_{kq}$ can be zeroed out by similarity transformations. When $k \in \mathcal{I}_K \setminus \mathcal{K}$, however, all $Q_k$ of these copies of $R_k$ are redundant because all of the $\vec{x}_{kq}$ are already zero. Eq. 15 of Main Text therefore becomes

$$\gamma \;=\; \deg \vec{x} - \deg \vec{x}^{\,\text{dist}} \;=\; \sum_{k \in \mathcal{K}} (Q_k - 1) \deg R_k \;\; + \sum_{k \in \mathcal{I}_K \setminus \mathcal{K}} Q_k \deg R_k. \tag{78}$$

# 6 Results for specific models (Table 1 of Main Text)

We now compute the number of parameters and gauge freedoms for a variety of one-hot equivariant models. To avoid conflicts in notation, we use $N$ and $n$ in place of $K$ and $k$ to parameterize orders of interactions. Note that $N$ here does not refer the number of sequences in $\mathcal{S}$.

## 6.1 $N$-order interaction model

Here we compute the number of parameters and gauge freedoms for the $N$-order interaction model. We begin by computing the degree of the model embedding $\vec{x}_{N\text{-order}}$. The position sets that define $\vec{x}_{N\text{-order}}$ are

$$\{A_j\}_{j=1}^{J} = \{U : U \subseteq I_L, |U| = N\}. \tag{79}$$

From this we see that there are $J = \binom{L}{N}$ distinct sets $A_j$, and that $|A_j| = N$ for every $j \in \mathcal{I}_J$. By Main Text Eq. 23, the degree of $\vec{x}_{N\text{-order}}$ is therefore

$$\deg \vec{x}_{N\text{-order}} \;=\; \sum_{j=1}^{J} \alpha^{|A_j|} \tag{80}$$

$$\;=\; \binom{L}{N} \alpha^N. \tag{81}$$

Next we compute the degree of the distilled embedding $\deg \vec{x}_{N\text{-order}}^{\,\text{dist}}$. The position sets that define $\vec{x}_{N\text{-order}}^{\,\text{dist}}$ are

$$\{B_k\}_{k=1}^{K} \;=\; \{V : V \subseteq A_j, j \in \mathcal{I}_J\} \tag{82}$$

$$\;=\; \{V : V \subseteq U, U \subseteq I_L, |U| = N\} \tag{83}$$

$$\;=\; \{V : V \subseteq I_L, |V| \leq N\} \tag{84}$$

$$\;=\; \bigcup_{n=0}^{N} \{V : V \subseteq I_L, |V| = n\}, \tag{85}$$

where the union is over disjoint sets. From this we see that, for each $n \in \{0, \ldots, N\}$, there are $\binom{L}{n}$ sets $B_k$ such that $|B_k| = n$. By Main Text Eq. 26, the degree of $\vec{x}^{\text{dist}}_{N\text{-order}}$ is therefore

$$\begin{aligned} \deg \vec{x}^{\text{dist}}_{N\text{-order}} &= \sum_{k=1}^{K} (\alpha - 1)^{|B_k|} &(86)\\ &= \sum_{n=0}^{N} \binom{L}{n} (\alpha - 1)^n. &(87) \end{aligned}$$

The number of parameters, $M_{N\text{-order}}$, and the number of gauge freedoms, $\gamma_{N\text{-order}}$, are therefore given by

$$\begin{aligned} M_{N\text{-order}} &= \deg \vec{x}_{N\text{-order}} &(88)\\ &= \binom{L}{N} \alpha^N, &(89)\\ \gamma_{N\text{-order}} &= \deg \vec{x}_{N\text{-order}} - \deg \vec{x}^{\text{dist}}_{N\text{-order}} &(90)\\ &= \binom{L}{N} \alpha^N - \sum_{n=0}^{N} \binom{L}{n} (\alpha - 1)^n. &(91) \end{aligned}$$

## 6.2 Hierarchical $N$-order interaction models

We define the hierarchical $N$-order interaction model to be the sum of $n$-order interaction models taken over all $n \in \{0, 1, \ldots, N\}$. The embedding of the hierarchical $N$-order interaction model is

$$\vec{x}_{\leq N\text{-order}} = \bigoplus_{n=0}^{N} \vec{x}_{n\text{-order}}. \quad (92)$$

The number of parameters of the hierarchical $N$-order interaction model is therefore

$$\begin{aligned} M_{\leq N\text{-order}} &= \sum_{n=0}^{N} M_{n\text{-order}} &(93)\\ &= \sum_{n=0}^{N} \binom{L}{n} \alpha^n. &(94) \end{aligned}$$

Since every parameter of the models of order $N - 1$ and lower is redundant with parameters of the model of order $N$, the number of gauge freedoms of the hierarchical $N$-order interaction model is

$$\begin{aligned} \gamma_{\leq N\text{-order}} &= \deg \vec{x}_{\leq N\text{-order}} - \deg \vec{x}^{\text{dist}}_{\leq N\text{-order}} &(95)\\ &= M_{\leq N\text{-order}} - \deg \vec{x}^{\text{dist}}_{N\text{-order}} &(96)\\ &= \sum_{n=0}^{N} \binom{L}{n} \alpha^n - \sum_{n=0}^{N} \binom{L}{n} (\alpha - 1)^n &(97)\\ &= \sum_{n=0}^{N} \binom{L}{n} [\alpha^n - (\alpha - 1)^n]. &(98) \end{aligned}$$

Additive models, pairwise interaction models, and all-order interaction models are special cases of the hierarchical $N$-order interaction model.

### 6.2.1 $N = 1$: Additive model

The additive model is a hierarchical 1-order interaction model. Setting $N = 1$, Eq. 94 gives

$$\begin{aligned} M_{\text{additive}} &= M_{\leq 1\text{-order}} &(99)\\ &= \binom{L}{0} + \binom{L}{1} \alpha &(100)\\ &= 1 + L\alpha, &(101) \end{aligned}$$

and Eq. 98 gives,

$$\begin{aligned}\gamma_{\text{additive}} &= \gamma_{\leq 1\text{-order}}, &(102)\\ &= \binom{L}{0}(1-1)+\binom{L}{1}(\alpha-(\alpha-1)), &(103)\\ &= L. &(104)\end{aligned}$$

#### 6.2.2 $N=2$: Pairwise-interaction model

The pairwise-interaciton model is a hierarchical 2-order interaction model. Setting $N=2$, Eq. 94 gives

$$\begin{aligned}M_{\text{pairwise}} &= M_{\leq 2\text{-order}} &(105)\\ &= \binom{L}{0}+\binom{L}{1}\alpha+\binom{L}{2}\alpha^2 &(106)\\ &= 1+L\alpha+\binom{L}{2}\alpha^2, &(107)\end{aligned}$$

and Eq. 98 gives

$$\begin{aligned}\gamma_{\text{pairwise}} &= \gamma_{\leq 2\text{-order}} &(108)\\ &= \binom{L}{0}(1-1)+\binom{L}{1}(\alpha-(\alpha-1))+\binom{L}{2}(\alpha^2-(\alpha-1)^2) &(109)\\ &= L+\binom{L}{2}(2\alpha-1). &(110)\end{aligned}$$

#### 6.2.3 $N=L$: All-order interaction model

The all-order interaction model is a hierarchical $L$-order interaction model. Setting $N=L$, Eq. 94 gives

$$\begin{aligned}M_{\text{all-order}} &= M_{\leq L\text{-order}} &(111)\\ &= \sum_{n=0}^{L}\binom{L}{n}\alpha^n &(112)\\ &= (\alpha+1)^L, &(113)\end{aligned}$$

and Eq. 98 gives,

$$\begin{aligned}\gamma_{\text{all-order}} &= \gamma_{\leq L\text{-order}}, &(114)\\ &= \sum_{n=0}^{L}\binom{L}{n}\left[\alpha^n-(\alpha-1)^n\right], &(115)\\ &= (\alpha+1)^L-\alpha^L. &(116)\end{aligned}$$

### 6.3 $N$-adjacent interaction model

Here we compute the number of parameters and gauge freedoms for the $N$-adjacent interaction model. To facilitate computations, we define $\operatorname{span} U = \max(U)-\min(U)+1$ for any nonempty set $U \subseteq \mathcal{I}_l$, as well as $\operatorname{span}\emptyset = 0$. We also define the 0-adjacent interaction model to be the constant model, i.e. the model that corresponds to the embedding $\vec{x}_{\text{constant}} = \vec{x}^{\,\text{triv}}$, which has $M_{\text{constant}} = 1$ parameters and $\gamma_{\text{constant}} = 0$ gauge freedoms.

For $N \geq 1$, we begin by computing the degree of the embedding $\vec{x}_{N\text{-adjacent}}$. The position sets that define $\vec{x}_{N\text{-adjacent}}$ are

$$\begin{aligned}\{A_j\}_{j=1}^{J} &= \{U : U \subseteq I_L,\ |U| = N,\ \operatorname{span}(U) = N\} &(117)\\ &= \{U_l : l \in \mathcal{I}_{L-N+1}\}, \quad \text{where} \quad U_l = \{l,\ldots,l+N-1\}. &(118)\end{aligned}$$

From this we see that there are $J = L-N+1$ distinct sets $A_j$, and that $|A_j| = N$ for every $j \in \mathcal{I}_J$. By Main Text Eq. 23, the degree of $\vec{x}_{N\text{-adjacent}}$ is therefore

$$\begin{aligned}\deg \vec{x}_{N\text{-adjacent}} &= \sum_{j=1}^{J}\alpha^{|A_j|} &(119)\\ &= (L-N+1)\alpha^N. &(120)\end{aligned}$$

Next we compute the degree of the distilled embedding $\vec{x}^{\,\text{dist}}_{N\text{-adjacent}}$. The position sets that define $\vec{x}^{\,\text{dist}}_{N\text{-adjacent}}$ are

$$\begin{aligned}
\{B_k\}_{k=1}^K &= \{V : V \subseteq A_j,\, j \in \mathcal{I}_J\} && (121)\\
&= \{V : V \subseteq U,\ U \subseteq I_L,\ |U| = N,\ \text{span}\,(U) = N\} && (122)\\
&= \{V : V \subseteq I_L,\ |V| \le N,\ \text{span}\,(V) \le N\} && (123)\\
&= \bigcup_{p=0}^{N} \bigcup_{n=0}^{p} \{V : V \subseteq I_L,\ |V| = n,\ \text{span}\,(V) = p\}\,, && (124)
\end{aligned}$$

where the unions are over disjoint sets, and we have used the fact that $|V| \le \text{span}\,(V)$ for all $V \subseteq \mathcal{I}_L$. By Main Text Eq. 26, the degree of $\vec{x}^{\,\text{dist}}$ is therefore

$$\begin{aligned}
\deg \vec{x}^{\,\text{dist}}_{N\text{-adjacent}} &= \sum_{k=1}^{K} (\alpha-1)^{|B_k|} && (125)\\
&= \sum_{p=0}^{N} \sum_{n=0}^{p} z(n,p)(\alpha-1)^n, && (126)
\end{aligned}$$

where $z(n,p)$ is the number of subsets of $\mathcal{I}_L$ that have $n$ elements and span $p$.

- For $n = 0$, only $V = \emptyset$ has span $p = 0$. Therefore $z(0,0) = 1$, and $z(0,p) = 0$ for all $p \neq 0$.
- For $n = 1$, there are $L$ subsets: $U = \{l\}$ for every $l \in \mathcal{I}_L$. Each of these subsets has span $p = 1$. Therefore $z(1,1) = L$ and $z(1,p) = 0$ for all $p \neq 1$.
- For every $n \in \{2,\dots,N\}$, there is at least one subset $U$ having span $p$ for every $p \in \{2,\dots,N\}$. Given the endpoints of $U$, there are $\binom{p-2}{n-2}$ ways to choose the elements of $U$. And given $p$, there are $L-p+1$ possible choices for the endpoints of $U$. Therefore, $z(n,p) = (L-p+1)\binom{p-2}{n-2}$.

Continuing from Eq. 126,

$$\begin{aligned}
\dim \vec{x}^{\,\text{dist}}_{N\text{-adjacent}} &= 1 + L(\alpha-1) + \sum_{p=2}^{N}\sum_{n=2}^{N} \binom{p-2}{n-2}(L-p+1)(\alpha-1)^n && (127)\\
&= 1 + L(\alpha-1) + (\alpha-1)^2 \sum_{p=2}^{N} (L-p+1)\alpha^{p-2} && (128)\\
&= 1 + L(\alpha-1) + (\alpha-1)^2 (L-1) \sum_{m=0}^{N-2} \alpha^m - (\alpha-1)^2 \sum_{m=0}^{N-2} m\alpha^m. && (129)
\end{aligned}$$

Setting $M = N-2$, and using

$$\sum_{m=0}^{M} \alpha^m = \frac{\alpha^{M+1}-1}{\alpha-1}, \tag{130}$$

and

$$\sum_{m=0}^{M} m\alpha^m = \alpha \frac{d}{d\alpha} \sum_{m=0}^{M} \alpha^m = \frac{\alpha}{(\alpha-1)^2}\left[1 - \alpha^{M+1} + (M+1)(\alpha-1)\alpha^M\right], \tag{131}$$

we get (after a remarkable simplification),

$$\dim \vec{x}^{\,\text{dist}}_{N\text{-adjacent}} = (L-N+1)\alpha^N - (L-N)\alpha^{N-1}. \tag{132}$$

The number of parameters, $M_{N\text{-adjacent}}$, and the number of gauge freedoms, $\gamma_{N\text{-adjacent}}$, is (for $N \ge 1$) therefore given by

$$\begin{aligned}
M_{N\text{-adjacent}} &= \dim \vec{x}_{N\text{-adjacent}}, && (133)\\
&= (L-N+1)\alpha^N, && (134)\\
\gamma_{N\text{-adjacent}} &= \dim \vec{x}_{N\text{-adjacent}} - \dim \vec{x}^{\,\text{dist}}_{N\text{-adjacent}}, && (135)\\
&= (L-N)\alpha^{N-1}. && (136)
\end{aligned}$$

Note the curious fact that the number of gauge freedoms for the $N$-adjacent model is equal to the number of parameters for the $(N-1)$-adjacent model, i.e.

$$\gamma_{N\text{-adjacent}} = M_{(N-1)\text{-adjacent}}. \tag{137}$$

## 6.4 Hierarchical $N$-adjacent interaction models

We define the hierarchical $N$-adjacent interaction model to be the sum of $n$-adjacent interaction terms taken over all $n \in \{0, 1, \ldots, N\}$. The embedding of the hierarchical $N$-adjacent interaction model is

$$\vec{x}_{\leq N\text{-adjacent}} = \bigoplus_{n=0}^{N} \vec{x}_{n\text{-adjacent}}. \tag{138}$$

The number of parameters of the hierarchical $N$-adjacent interaction model is therefore,

$$\begin{aligned} M_{\leq N\text{-adjacent}} &= \sum_{n=0}^{N} M_{n\text{-adjacent}}, & (139)\\ &= 1 + \sum_{n=1}^{N} (L-n+1)\alpha^n, & (140)\\ &= 1 + \frac{\alpha}{(\alpha-1)^2}\left[(L-N+1)\alpha^{N+1} - (L-N)\alpha^N - (L+1)\alpha + L\right]. & (141)\end{aligned}$$

Since every parameter of the models of order $N-1$ and lower is redundant with parameters of the model of order $N$, the number of gauge freedoms of the hierarchical $N$-adjacent interaction model,

$$\begin{aligned} \gamma_{\leq\, N\text{-adjacant}} &= \deg \vec{x}_{\leq N\text{-adjacent}} - \deg \vec{x}^{\,\text{dist}}_{\leq N\text{-adjacent}} & (142)\\ &= M_{\leq N\text{-adjacent}} - \deg \vec{x}^{\,\text{dist}}_{N\text{-adjacent}} & (143)\\ &= 1 + \frac{\alpha}{(\alpha-1)^2}\left[(L-N+1)\alpha^{N+1} - (L-N)\alpha^N - (L+1)\alpha + L\right] & (144)\\ &\quad -(L-N+1)\alpha^N + (L-N)\alpha^{N-1}, & (145)\\ &= (L-N)\alpha^{N-1} + 1 + \frac{\alpha}{(\alpha-1)^2}\left[(L-N+2)\alpha^N - (L-N+1)\alpha^{N-1} - (L+1)\alpha + L\right]. & (146)\end{aligned}$$

Additive models, nearest-neighbor interaction models, and all-order interaction models are special cases of the hierarchical $N$-adjacent interaction model.

### 6.4.1 $N = 1$: Additive model

The additive model is a hierarchical 1-adjacent interaction model. Setting $N = 1$, Eq. 141 gives

$$\begin{aligned} M_{\text{additive}} &= M_{\leq 1\text{-adjacent}} & (147)\\ &= 1 + \frac{\alpha}{(\alpha-1)^2}\left[L\alpha^2 - (L-1)\alpha - (L+1)\alpha + L\right] & (148)\\ &= 1 + L\alpha, & (149)\end{aligned}$$

and Eq. 146 gives

$$\begin{aligned} \gamma_{\text{additive}} &= \gamma_{\leq 1\text{-adjacent}} & (150)\\ &= (L-1) + 1 + \frac{\alpha}{(\alpha-1)^2}\left[(L+1)\alpha - L - (L+1)\alpha + L\right] & (151)\\ &= L. & (152)\end{aligned}$$

### 6.4.2 $N = 2$: Nearest-neighbor interaction model

The nearest-neighbor interaction model is a hierarchical 2-adjacent interaction model. Setting $N = 2$, Eq. 141 gives

$$\begin{aligned} M_{\text{neighbor}} &= M_{\leq 2\text{-adjacent}} & (153)\\ &= 1 + \frac{\alpha}{(\alpha-1)^2}\left[(L-1)\alpha^3 - (L-2)\alpha^2 - (L+1)\alpha + L\right] & (154)\\ &= 1 + L\alpha + (L-1)\alpha^2, & (155)\end{aligned}$$

and Eq. 146 gives

$$\begin{aligned} \gamma_{\text{neighbor}} &= \gamma_{\leq 2\text{-adjacent}} & (156)\\ &= (L-2)\alpha + 1 + \frac{\alpha}{(\alpha-1)}\left[L\alpha^2 - (L-1)\alpha - (L+1)\alpha + L\right] & (157)\\ &= 1 + 2(L-1)\alpha. & (158)\end{aligned}$$

### 6.4.3 $N = L$: All-adjacent interaction model

The all-order adjacent model is a hierarchical $L$-adjacent interaction model. Setting $N = L$, Eq. 141 gives

$$\begin{aligned} M_{\text{all-adjacent}} &= M_{\leq L\text{-adjacent}} & (159)\\ &= 1 + \frac{\alpha}{(\alpha-1)^2}\left[\alpha^{L+1} - (L+1)\alpha + L\right], & (160)\end{aligned}$$

and Eq. 146 gives,

$$\begin{aligned} \gamma_{\text{all-adjacent}} &= \gamma_{\leq L\text{-adjacent}} & (161)\\ &= 1 + \frac{\alpha}{(\alpha-1)^2}\left[2\alpha^{L} - \alpha^{L-1} - (L+1)\alpha + L\right]. & (162)\end{aligned}$$

# 7 Results for other symmetry groups: $H_{\text{GCP}}$, $H_{\text{PP}}$, and $H_{\text{PSCP}}$

## 7.1 Distillation does not work for $H_{\text{GCP}}$ or $H_{\text{PP}}$

Embedding distillation does not work for $H_{\text{GCP}}$ (the group of global character permutations) or for $H_{\text{PP}}$ (the group of position permutations). The reason is that Prop. 7 (Theorem 1 of Main Text), does not hold for either of these groups: two embeddings that transform under the same representation are not necessarily proportional (or, in fact, related by any similarity transformation). Consider, for example, the following degree 1 embeddings:

$$\vec{x}_1(s) = \begin{cases} [1] \text{ if } s_l = s'_l \text{ for all } l, l' \in \mathcal{I}_L \\ [0] \text{ otherwise} \end{cases}, \quad (163)$$

$$\vec{x}_2(s) = \begin{cases} [0] \text{ if } s_l = s'_l \text{ for all } l, l' \in \mathcal{I}_L \\ [1] \text{ otherwise} \end{cases}. \quad (164)$$

Both $\vec{x}_1$ and $\vec{x}_2$ are invariant under $H_{\text{GCP}}$ and $H_{\text{PP}}$, and therefore transform under the trivial representation of both groups. However, $\vec{x}_1$ and $\vec{x}_2$ are not related by any similarity transformation, as no similarity transformation can map a zero vector to a nonzero vector. Consequently, distillation cannot be used to determine the gauge freedoms of either embedding, and the number of gauge freedoms cannot be computed by summing the degrees of the redundant irreducible representations under which these embeddings transform.

To see explicitly how distillation fails for $H_{\text{GCP}}$ and for $H_{\text{PP}}$, consider the additive embedding:

$$\vec{x}_{\text{additive}} = \vec{x}^{\,\text{triv}} \oplus \bigoplus_{l=1}^{L} \vec{x}_l^{\,\text{ohe}}. \quad (165)$$

Under $H_{\text{GCP}}$, each $\vec{x}_l^{\,\text{ohe}}$ transforms under the same representation: $R^{\text{def}}_{(\alpha)}$, the defining representation of $S_\alpha$ (we indicate the degree of the representation in the subscript for clarity). Using $R^{\text{def}}_{(\alpha)} \simeq R^{\text{triv}} \oplus R^{\text{std}}_{(\alpha-1)}$, where $R^{\text{std}}_{(\alpha-1)}$ is the standard representation of $S_\alpha$, we find that $\vec{x}_{\text{additive}}$ transforms under $H_{\text{GCP}}$ according to the representation

$$\begin{aligned} R^{\text{add}}_{\text{GCP}} &= R^{\text{triv}} \oplus [I_L \otimes R^{\text{def}}_{(\alpha)}], & (166)\\ &\simeq R^{\text{triv}} \oplus L R^{\text{def}}_{(\alpha)}, & (167)\\ &\simeq R^{\text{triv}} \oplus L[R^{\text{triv}} \oplus R^{\text{std}}_{(\alpha-1)}], & (168)\\ &\simeq [L+1] R^{\text{triv}} \oplus L R^{\text{std}}_{(\alpha-1)}, & (169)\\ &\simeq R^{\text{dist}}_{\text{GCP}} \oplus R^{\text{redun}}_{\text{GCP}}, & (170)\end{aligned}$$

where the distilled representation is

$$R^{\text{dist}}_{\text{GCP}} = R^{\text{triv}} \oplus R^{\text{std}}_{(\alpha-1)}, \quad (171)$$

and the redundant irreducible representations are collected into

$$R^{\text{redun}}_{\text{GCP}} = L R^{\text{triv}} \oplus (L-1) R^{\text{std}}_{(\alpha-1)}. \quad (172)$$

We thus see that

$$\deg R^{\text{redun}}_{\text{GCP}} = \alpha L - \alpha + 1. \quad (173)$$

This does not match the number of gauge freedoms $\gamma_{\text{additive}} = L$. The reason is that each copy of $R^{\text{std}}_{(\alpha-1)}$ transforms a simplex embedding of $\mathcal{A}$, one for each position in $\mathcal{I}_L$, and these embeddings are not equivalent. Similarly, $H_{\text{PP}}$ acts on $\vec{x}_{\text{additive}}$ via the representation

$$
\begin{aligned}
R^{\text{add}}_{\text{PP}} &= R^{\text{triv}} \oplus [R^{\text{def}}_{(L)} \otimes I_\alpha], & (174)\\
&= R^{\text{triv}} \oplus \alpha R^{\text{def}}_{(L)}, & (175)\\
&\simeq R^{\text{triv}} \oplus \alpha[R^{\text{triv}} \oplus R^{\text{std}}_{(L-1)}], & (176)\\
&\simeq [1+\alpha]R^{\text{triv}} \oplus \alpha R^{\text{std}}_{(L-1)}, & (177)\\
&\simeq R^{\text{dist}}_{\text{PP}} \oplus R^{\text{redun}}_{\text{PP}}, & (178)
\end{aligned}
$$

where the distilled representation is

$$R^{\text{dist}} = R^{\text{triv}} \oplus R^{\text{std}}_{(L-1)}, \tag{179}$$

and the redundant irreducible representations are collected into

$$R^{\text{redun}}_{\text{PP}} = \alpha R^{\text{triv}} \oplus (\alpha-1) R^{\text{std}}_{(L-1)}. \tag{180}$$

We thus see that

$$\deg R^{\text{redun}}_{\text{PP}} = \alpha L - L + 1. \tag{181}$$

This does not match the number of gauge freedoms $\gamma_{\text{additive}} = L$. The reason is that each copy of $R^{\text{std}}_{(L-1)}$ transforms a simplex embedding of $\mathcal{I}_L$, one for each character in $\mathcal{A}$, and these embeddings are not equivalent.

## 7.2 Distillation does work for $H_{\text{Ham}}$

Our embedding distillation procedure does work for $H_{\text{Ham}}$, the symmetry group of the Hamming graph. $H_{\text{Ham}}$ is given by the semidirect product

$$H_{\text{Ham}} = H_{\text{PSCP}} \rtimes H_{\text{PP}} \tag{182}$$

Note that $H_{\text{PSCP}}$ is a normal subgroup of $H_{\text{Ham}}$.

**Proposition 8.** *Nonzero sequence embeddings that transform under the same irreducible representation of $H_{\text{Ham}}$ are equivalent.*

*Proof.* Assume $\vec{x}$ and $\vec{y}$ are two nonzero embeddings that transform under the same irreducible representation $R$ of $H_{\text{Ham}}$. Let $R'$ denote $R$ restricted to $H_{\text{PSCP}}$. By Maschke's theorem, there is a similarity transformation matrix $T$ such that

$$R' = T\left[\bigoplus_{k=1}^{K} R'_k\right] T^{-1}, \tag{183}$$

where, for each $k = 1, \ldots, K$, $R_k$ is an irreducible representation of $H_{\text{PSCP}}$. Consequently,

$$\vec{x} = T\left[\bigoplus_{k=1}^{K} \vec{x}_k\right], \quad \text{and} \quad \vec{y} = T\left[\bigoplus_{k=1}^{K} \vec{y}_k\right], \tag{184}$$

where, for each $k \in \mathcal{I}_K$, both $\vec{x}_k$ and $\vec{y}_k$ transform under $R'_k$. We now show that all $\vec{x}_k$ and $\vec{y}_k$ are nonzero. Let $m_k = \deg R_k$, $\mathcal{K} = \left\{k : k \in \mathcal{I}_K,\ \vec{x}_k = \vec{0}\right\}$, and define the vector space

$$V = T^{-1\dagger}\left[\bigoplus_{k=1}^{K} \begin{cases} \mathbb{C}^{m_k} & \text{if } k \in \mathcal{K}, \\ \left\{\vec{0}\right\} & \text{otherwise} \end{cases}\right]. \tag{185}$$

It is readily see that $V$ is orthogonal to span $\vec{x}$, i.e., $\vec{v}^\dagger \vec{x}(s) = 0$ for all $v \in V$ and all $s \in \mathcal{S}$. But we know from Prop. 3 that, because $\vec{x}$ transforms under an irreducible representation of $H_{\text{Ham}}$, span $\vec{x}$ has full rank. Consequently, $\mathcal{K} = \emptyset$, and thus all $\vec{x}_k$ are nonzero. Similarly, all $\vec{y}_k$ are nonzero. Since both $\vec{x}_k$ and $\vec{y}_k$ are nonzero embeddings that transform under the same irreducible representation of $H_{\text{PSCP}}$, Proposition 7 tells us that $\vec{y}_k = c_k \vec{x}_k$ for some nonzero scalar $c_k \in \mathbb{C}$. Therefore,

$$\vec{y} = TCT^{-1}\vec{x} \quad \text{where} \quad C = \bigoplus_{k=1}^{K} c_k I_{m_k}. \tag{186}$$

This shows that $\vec{x}$ and $\vec{y}$ are related by a similarity transformation, thereby completing the proof. □

# 8 Distillation algorithm

We now describe the embedding distillation algorithm discussed in the section "Computational analysis of general equivalent one-hot models" of the main text and illustrated in main text Fig. 3. Python code implementing this algorithm is available at https://github.com/jbkinney/23_posfai.

## 8.1 Overview of the algorithm

Let $\vec{x}$ be an equivariant one-hot encoding of sequences as defined in the main text. Specifically, define

$$\vec{x} = \bigoplus_{j=1}^{J} \bigotimes_{l \in A_j} \vec{x}_l^{\,\text{ohe}}, \tag{187}$$

where each $A_j$, $j \in \mathcal{I}_J$, denotes an ordered set of sequence positions, and $(A_j)_{j=1}^{J}$ denotes an ordered set of position subsets the sets of positions used to construct the embedding. Distillation consists of multiplying $\vec{x}$ by a "distillation matrix", $T$, such that

$$T\vec{x} = \vec{x}_{\text{dist}} \oplus \vec{0}_\gamma, \tag{188}$$

where $\vec{x}_{\text{dist}}$ is the full-rank distilled embedding, $\vec{0}_\gamma$ is a vector of $\gamma$ zeros, and $\gamma$ is the number of gauge freedoms of $\vec{x}$. As in the main text, construct $T$ by expressing it as

$$T = T_{\text{sort}}\, T_{\text{thin}}\, T_{\text{decom}}. \tag{189}$$

We now describe the operations each of the three factors above perform:

1. $T_{\text{decom}}$ decomposes each Kronecker product of $\vec{x}_l^{\,\text{ohe}}$ into a direct sum of $\vec{x}^{\,\text{triv}}$ and Kronecker products of $\vec{x}_l^{\,\text{sim}}$. Specifically, multiplying Eq. 187 by $T_{\text{decom}}$ yields,

$$T_{\text{decom}}\,\vec{x} \quad = \quad \bigoplus_{p=1}^{P} \vec{x}_{k_p}, \tag{190}$$

where each $\vec{x}_k$ is an irreducible representation given by

$$\vec{x}_k = \bigotimes_{l \in B_k} \vec{x}_l^{\,\text{sim}}, \tag{191}$$

each $B_k$ is a distinct ordered set of positions (in which positions are listed in increasing order), $(B_k)_{k=1}^{K}$ is an ordered set comprising all unique subsets of positions found within all the $A_j$, and $(k_p)_{p=1}^{P}$ is an ordered set of (generally non-unique) indices within $\mathcal{I}_K$.

2. $T_{\text{thin}}$ thins the resulting embedding by zeroing-out all redundant copies of each irreducible embedding. Specifically, multiplying Eq. 190 by $T_{\text{thin}}$ yields

$$T_{\text{thin}}\, T_{\text{decom}}\,\vec{x} = \bigoplus_{p=1}^{P} \begin{cases} \vec{x}_{k_p} & \text{if } k_p \neq k_{p'} \text{ for any } p' < p \\ \vec{0}_{m_{k_p}} & \text{otherwise} \end{cases}, \tag{192}$$

where each $m_k = (\alpha - 1)^{|B_k|}$ is the degree of $\vec{x}_k$.

3. $T_{\text{sort}}$ sorts the embeddings in the direct sum so that all nonzero embeddings come first. Specifically, multiplying Eq. 192 by $T_{\text{sort}}$ yields

$$T_{\text{sort}}\, T_{\text{thin}}\, T_{\text{decom}}\,\vec{x} = \vec{x}^{\,\text{dist}} \oplus \vec{0}_\gamma, \tag{193}$$

where

$$\vec{x}^{\,\text{dist}} = \bigoplus_{k=1}^{K} \vec{x}_k. \tag{194}$$

We now show how to compute each of these three matrix factors. We find that the resulting distillation matrix $T$ is sparse (because $T_{\text{decom}}$, $T_{\text{thin}}$, and $T_{\text{sort}}$ are sparse) and that all nonzero entries of $T$ are either $+1$ or $-1$. We also show how to compute the inverse of each factor without having to invert any matrices, thereby allowing the rapid computation of

$$T^{-1} = T_{\text{decom}}^{-1}\, T_{\text{thin}}^{-1}\, T_{\text{sort}}^{-1}. \tag{195}$$

In what follows, we index vector and matrix elements starting from zero.

## 8.2 General one-hot and simplex encodings

Consider a general alphabet $\mathcal{A} = (c_1, \ldots, c_\alpha)$, and let $s$ be any sequence in $\mathcal{S}$. We define the position-specific one-hot encoding, $\vec{x}^{\,\text{ohe}}$, to be an $\alpha$-dimensional vector having elements

$$[\vec{x}_l^{\,\text{ohe}}(s)]_{i-1} = \begin{cases} 1 & \text{if } c_i = s_l, \\ 0 & \text{otherwise}, \end{cases} . \tag{196}$$

We define the position-specific simplex encoding, $\vec{x}^{\,\text{sim}}$, to be an $(\alpha - 1)$-dimensional vector having elements

$$[\vec{x}_l^{\,\text{sim}}(s)]_{i-1} = \begin{cases} 1 & \text{if } s_l = c_i \text{ and } i \neq \alpha, \\ -1 & \text{if } s_l = c_\alpha, \\ 0 & \text{otherwise.} \end{cases} \tag{197}$$

Finally, we define the trivial embedding, $\vec{x}^{\,\text{triv}}$, to be a one-dimensional vector given by

$$\vec{x}^{\,\text{triv}}(s) = [1]. \tag{198}$$

## 8.3 Computation of the decomposition matrix $T_{\text{decom}}$.

We build the decomposition matrix, $T_{\text{decom}}$, as a direct sum of individual $N$th order decomposition matrices, $T^{(N)}_{\text{decom}}$. Specifically,

$$T_{\text{decom}} = \bigoplus_{j=1}^{J} T^{(N_j)}_{\text{decom}}, \tag{199}$$

where $N_j = |A_j|$ for all $j \in \mathcal{I}_J$. Given $N$ we define $T^{(N)}_{\text{decom}}$ so that

$$T^{(N)}_{\text{decom}} \left[ \bigotimes_{n=1}^{N} \vec{x}_{l_n}^{\,\text{ohe}} \right] = \bigoplus_{B \subseteq A} \left[ \bigotimes_{l \in B} \vec{x}_l^{\,\text{sim}} \right] \tag{200}$$

for any subset of positions $A$ that has $N$ elements. We computationally construct $T^{(N)}_{\text{decom}}$ by recursion using

$$T^{(N)}_{\text{decom}} = T^{(N)}_{\text{perm}} \left[ T^{(N-1)}_{\text{decom}} \otimes T^{(1)}_{\text{decom}} \right]. \tag{201}$$

Using this recursion relation requires defining $T^{(1)}_{\text{decom}}$ and $T^{(N)}_{\text{perm}}$:

- $T^{(1)}_{\text{decom}}$ is an $\alpha \times \alpha$ matrix having elements

$$\left[ T^{(1)}_{\text{decom}} \right]_{ij} = \begin{cases} 1 & \text{if } i = 0, \\ -1 & \text{if } 0 < i < \alpha, \ j = \alpha - 1, \\ 1 & \text{if } 0 < i < \alpha, \ j = i - 1, \\ 0 & \text{otherwise}, \end{cases} \tag{202}$$

  for $i, j \in \{0, \ldots, \alpha - 1\}$. Note that $T^{(1)}_{\text{decom}}$ matches Eq. 12 when $\alpha = 4$.

- $T^{(N)}_{\text{perm}}$ is an $\alpha^N \times \alpha^N$ permutation matrix having elements

$$\left[ T^{(N)}_{\text{perm}} \right]_{ij} = \begin{cases} 1 & \text{if } 0 \leq i < \alpha^{N-1} \text{ and } \ j = \alpha i, \\ 1 & \text{if } \alpha^{N-1} \leq i < \alpha^N \text{ and } j = 1 + (i - \alpha^{N-1}) + \lfloor (i - \alpha^{N-1})/(\alpha - 1) \rfloor, \\ 0 & \text{otherwise.} \end{cases} \tag{203}$$

  for $i, j \in \{0, \ldots, \alpha^N - 1\}$. $T_{\text{perm}}$ needed because Kronecker products do not distribute over direct sums. Specifically, for a general vector $\vec{v}$ having $\alpha^N$ elements,

$$\vec{v} \otimes \left( \vec{x}^{\,\text{triv}} \oplus \vec{x}_l^{\,\text{sim}} \right) \neq \left( \vec{v} \otimes \vec{x}^{\,\text{triv}} \right) \oplus \left( \vec{v} \otimes \vec{x}_l^{\,\text{sim}} \right). \tag{204}$$

  The matrix $T^{(N)}_{\text{perm}}$ fixes this inequality, i.e.,

$$T_{\text{perm}} \left[ \vec{v} \otimes \left( \vec{x}^{\,\text{triv}} \oplus \vec{x}_l^{\,\text{sim}} \right) \right] = \left( \vec{v} \otimes \vec{x}^{\,\text{triv}} \right) \oplus \left( \vec{v} \otimes \vec{x}_l^{\,\text{sim}} \right). \tag{205}$$

Similarly, we build the inverse of the decomposition matrix, $T_{\text{decom}}^{-1}$, as a direct sum of $N$'th order inverse decomposition matrices $\left(T_{\text{decom}}^{(N)}\right)^{-1}$, which are computed by recursion using

$$\left(T_{\text{decom}}^{(N)}\right)^{-1} = \left[\left(T_{\text{decom}}^{(N-1)}\right)^{-1} \otimes \left(T_{\text{decom}}^{(1)}\right)^{-1}\right] \left(T_{\text{perm}}^{(N)}\right)^{-1}, \tag{206}$$

with

$$\left[\left(T_{\text{decom}}^{(1)}\right)^{-1}\right]_{ij} = \frac{1}{\alpha} \times \begin{cases} 1 & \text{if } j = 0, \\ (\alpha - 1) & \text{if } 0 < j < \alpha,\ i = j-1, \\ -1 & \text{otherwise,} \end{cases} \tag{207}$$

for $i, j \in \{0, \dots, \alpha - 1\}$. Note that $\left(T_{\text{decom}}^{(1)}\right)^{-1}$ matches Eq. 12 when $\alpha = 4$. Also note that, because $T_{\text{perm}}^{(N)}$ is a permutation matrix,

$$\left(T_{\text{perm}}^{(N)}\right)^{-1} = \left(T_{\text{perm}}^{(N)}\right)^{\top}. \tag{208}$$

## 8.4 Computation of the thinning matrix $T_{\text{thin}}$

For each $p \in \mathcal{I}_P$, define the index offset function

$$\text{offset}(p) = \sum_{p' < p} m_{k_{p'}}, \tag{209}$$

and the first occurence function

$$\text{first}(p) = \min \left\{p' : k'_p = k_p\right\}. \tag{210}$$

The thinning matrix, $T_{\text{thin}}$, is an $M \times M$ having elements

$$[T_{\text{thin}}]_{ij} = \begin{cases} 1 & \text{if } i = j, \\ -1 & \text{if } i \neq j,\ i = \text{offset}(p) + h, \text{ and } j = \text{offset}(\text{first}(p)) + h \text{ for some } p \in \mathcal{I}_P,\ h \in \left\{0, \dots, m_{k_p} - 1\right\}, \\ 0 & \text{otherwise,} \end{cases} \tag{211}$$

for $i, j \in \{0, \dots, M-1\}$. One can readily verify that the inverse of the thinning matrix thus has elements

$$\left[T_{\text{thin}}^{-1}\right]_{ij} = \begin{cases} 1 & \text{if } i = j, \\ 1 & \text{if } i \neq j,\ i = \text{offset}(p) + h, \text{ and } j = \text{offset}(\text{first}(p)) + h \text{ for some } p \in \mathcal{I}_P,\ h \in \left\{0, \dots, m_{k_p} - 1\right\}. \\ 0 & \text{otherwise.} \end{cases} \tag{212}$$

## 8.5 Computation of the sorting matrix $T_{\text{sort}}$

We define the sorted index offset function as

$$\text{sortedoffset}(p) \quad = \quad \begin{cases} \text{offest}_1(p) & \text{if } p = \text{first}(p), \\ \text{offest}_2(p) & \text{if } p \neq \text{first}(p). \end{cases} \tag{213}$$

where

$$\text{offset}_1(p) \quad = \quad \sum_{\substack{p' < p: \\ p' = \text{first}(p')}} m_{k_{p'}}, \tag{214}$$

$$\text{offset}_2(p) \quad = \quad \sum_{\substack{p' \leq P: \\ p' = \text{first}(p')}} m_{k_{p'}} + \sum_{\substack{p' < p: \\ p' \neq \text{first}(p')}} m_{k_{p'}}. \tag{215}$$

The sorting matrix, $T_{\text{sort}}$, is an $M \times M$ matrix having elements

$$[T_{\text{sort}}]_{ij} = \begin{cases} 1 & \text{if } i = \text{sortedoffset}(p) + h \text{ and } j = \text{offset}(p) + h \text{ for some } p \in \mathcal{I}_P \text{ and } h \in \mathcal{I}_{m_{k_p}} - 1, \\ 0 & \text{otherwise,} \end{cases} \tag{216}$$

for $i, j \in \{0, \dots, M-1\}$. It is readily see that $T_{\text{sort}}$ is a permutation matrix. Consequently, the inverse of $T_{\text{sort}}$ is given by its transpose, i.e.,

$$T_{\text{sort}}^{-1} = T_{\text{sort}}^{\top}. \tag{217}$$

# 9 Parameter interpretation

## 9.1 Properties of allelic models

**Proposition 9.** *If the parameters of an equivariant model quantify allelic effects, then those parameters transform under a permutation representation of $H_{\text{PSCP}}$.*

*Proof.* Because $H_{\text{PSCP}}$ is transitive, the orbit of $\mathcal{S}_i$ in $H_{\text{PSCP}}$ consists of a tiling set of allelic states, $\{\mathcal{S}_i : i \in U\}$, where $U$ is an appropriate set of alleles. For the model embedding $\vec{x}$ to be equivariant, the vector $\vec{x}$ must contain among its elements the full set of sequence features $\{x_i : i \in U\}$. And because every $h \in H_{\text{PSCP}}$ permutes the set of allelic states $\{\mathcal{S}_i : i \in U\}$, the corresponding representation of $h$ that acts on $\vec{x}$ must be a permutation representation. □

## 9.2 One-hot models provide a lower bound on gauge freedoms

Let us consider an encoding of the form

$$\vec{x}(s) = \bigoplus \vec{x}_{l_1 \dots l_k}, \quad \text{where} \quad \vec{x}_{l_1 \dots l_k} := \vec{x}_{l_1}(s) \otimes \dots \otimes \vec{x}_{l_k}(s), \tag{218}$$

the direct sum goes over an arbitrarily chosen set of position sets $\{l_1, \dots, l_k\}$, and each $\vec{x}_{l_j} = (x^{i_1}_{l_1}, \dots, x^{i_k}_{l_k})$ is an arbitrary single-position sequence encoding, where each $x^{i_j}_{l_j}$ is a function that only depends on sequence $s$ at position $l_j$. We assume that none of the single-position features are constant over all characters, i.e. if $x^i_l$ denotes the $i$th feature in the single-position encoding $\vec{x}_l$ then $x^i_l(c)$, $c \in \mathcal{A}$, is not a constant function. We note that the constant feature can be part of encoding $\vec{x}$, it corresponds to the empty set in the direct sum over position sets in (218).

Let $\gamma_{\vec{x}}$ denote the number of gauge freedoms of the model that uses encoding $\vec{x}$, and let $\gamma_{\vec{x}^{\text{ohe}}}$ denote the number of gauge freedoms of the model that uses the encoding of the same direct sum/direct product structure as $\vec{x}$, given in (218), but with $\vec{x}_{l_i} = \vec{x}^{\text{ohe}}_{l_i}$ for all $l_i$.

**Proposition 10.** *If a model built from encoding $\vec{x}$ given in formula (218) transforms under a permutation representation of $H_{\text{PSCP}}$, then the model has at least as many gauge freedoms as the corresponding one-hot model, i.e.*

$$\gamma_{\vec{x}} \geq \gamma_{\vec{x}^{\text{ohe}}}. \tag{219}$$

*Proof.* Let us assume we have an encoding $\vec{x}$ that satisfies the assumptions above, and let $R$ be the permutation representation under which it transforms. For each $h$, $R(h)$ is a permutation matrix, and therefore induces a permutation on the set of features which we denote by $h'$. With this notation, the equivariance defining equation becomes

$$\vec{x}(h(s)) = h'(\vec{x}(s)), \quad s \in \mathcal{S}, h \in H_{\text{PSCP}}. \tag{220}$$

We first show that, for each $h \in H_{\text{PSCP}}$, $h'$ does not mix features corresponding to different position sets, i.e. $h'$ maps each feature vector $\vec{x}_{l_1 \dots l_k}$ onto itself. First, if the encoding contains the constant feature $x_0$, we have $h'(x_0(s)) = x_0(h(s))$ =constant for all $s$, therefore $x_0$ is mapped to itself. (We note that we assumed that there is at most one constant feature in the encoding $\vec{x}$.). Next, let us consider how $h'$ acts on single-position features. Let $x^i_l$ denote the $i$th coordinate of the singe-position encoding $\vec{x}_l$. If $x^i_l$ is mapped to a possibly higher order feature $x^{i_1 \dots i_k}_{l_1 \dots l_k}$ ($k \geq 1$), then using the product form $h = h_1 \cdots h_L$, we have

$$x^i_l(h(s)) = x^{i_1 \dots i_k}_{l_1 \dots l_k}(s) = x^{i_1}_{l_1}(s) \cdot \ldots \cdot x^{i_k}_{l_k}(s). \tag{221}$$

We see that the left hand side function only depends on a sequence's character at position $l$, therefore, for any position $l_j \neq l$, if we change $s$ at $l_j$, the left hand side of the equation above does not change, while the right hand side does. Note that here we use the assumption that non of the single-position features $x^{i_j}_{l_j}(s)$ are constant functions of $s$. Therefore, we see that, to avoid contradiction, we need to have $k = 1$ and $l_1 = l$, that is, any single-position feature vector $\vec{x}_l(s)$ gets mapped onto itself by $h'$. Finally, consider how $h'$ acts on higher order features. By the definition of higher order feature vectors as tensor products of single-position feature vectors, and using the product form $h = h_1 \cdots h_L$, we obtain

$$x^{i_1 \dots i_k}_{l_1 \dots l_k}(h(s)) = \prod_{j=1}^{k} x^{i_j}_{l_j}(h(s)) = \prod_{j=1}^{k} x^{i_j}_{l_j}(h_{l_j}(s)) = \prod_{j=1}^{k} x^{i'_j}_{l_j}(s) = x^{i'_1 \dots i'_k}_{l_1 \dots l_k}(s), \tag{222}$$

where $x^{i'_{l_j}}_{l_j} := h'_{l_j}(x^{i_{l_j}}_{l_j})$. We thus find that $\vec{x}_{l_1 \dots l_k}$ also gets mapped onto itself by $h'$.

We conclude that for each $h \in H$, the permutation matrix $R(h)$ is block-diagonal with blocks $R_{l_1 \dots l_k}(h)$ corresponding to the position sets $l_1 < \ldots < l_k$ in formula (218). Therefore

$$\vec{x}(h(s)) = \bigoplus_{l_1 < \ldots < l_k} \vec{x}_{l_1 \dots l_k}(h(s)) = \bigoplus_{l_1 < \ldots < l_k} R_{l_1 \dots l_k}(h) \vec{x}_{l_1 \dots l_k}(s). \tag{223}$$

Considering a term in the right hand side expression above, we saw in equation (222) that how $R_{l_1\ldots l_k}(h)$ acts on $\vec{x}_{l_1\ldots l_k}$ is completely determined by how it acts on single-site feature vectors, specifically, $R_{l_1\ldots l_k}(h) = R_{l_1}(h) \otimes \ldots \otimes R_{l_k}(h)$, where $R_{l_j}(h)$ is the block of $R(h)$ that acts on the single-position feature vector $\vec{x}_{l_j}$. In summary, we found that

$$R = \bigoplus R_{l_1} \otimes \ldots \otimes R_{l_k}, \tag{224}$$

where each $R_{l_i}$ is permutation representation of $S_\alpha$.

Let us look at the Maschke decomposition of a factor $R_{l_j}$ in the expression above. A permutation representation is reducible, in particular, its Maschke decomposition always contains the trivial representation. From Proposition 6, we further know that the Maschke decomposition of $R_{l_j}$ can only contain copies of the trivial representation $R^{\text{triv}}$ and copies of the standard representation $R^{\text{std}}$ with degree $\alpha - 1$. But if it were to contain copies of only the trivial representation, then $\vec{x}_{l_j}$ would be the trivial embedding which would contradict our assumption that non of the first order features are constant over all characters. This means that the Maschke decomposition of $R_{l_j}$ also has to contain a copy of the standard representation. Therefore the Maschke decomposition of $R_{l_j}$ looks like

$$R_{l_j} \simeq Q^{\text{triv}} R^{\text{triv}} \oplus Q^{\text{std}} R^{\text{std}}, \tag{225}$$

where $Q^{\text{triv}} \geq 1$ and $Q^{\text{std}} \geq 1$.

Since $Q^{\text{triv}} = 1$ and $Q^{\text{std}} = 1$ in the Maschke decomposition of the corresponding one-hot model, we see that, in the distillation procedure, whenever a coordinate of $\vec{x}$ is zeroed out, so is the corresponding coordinate of $\vec{x}^{\text{ohe}}$, and therefore, $\gamma_{\vec{x}} \geq \gamma_{\vec{x}^{\text{ohe}}}$. □

# 10 Restriction to the reals

Suppose we have a real embedding $\vec{x}$. The following proposition shows that resulting the number of gauge freedoms ($\gamma = \dim G$) is the same regardless of whether we allow the parameters to be complex ($\vec{\theta} \in \mathbb{C}^M$) or we restrict them to the reals ($\vec{\theta} \in \mathbb{R}^M$). Moreover, the algorithm we derive above for identifying a sparse basis for $G$ in the context of generalized one-hot model yields basis vectors with real elements, and the projection matrices for the parametric gauges described in [1] all have real elements. Therefore, if $\vec{x}$ is real, all of the primary results in this manuscript and in our companion paper hold when model parameters are required to be real.

**Proposition 11.** *If $\vec{x}$ is a real embedding, then the corresponding space of gauge freedoms $G$ has real basis.*

*Proof.* Assume that $\vec{x}$ is a real embedding, i.e., that $\vec{x}(s) \in \mathbb{R}^M$ for every $s \in \mathcal{S}$. Let $X$ denote the design matrix of $\vec{x}$. Now choose any nonzero $\vec{g}_1 \in G$. Since $X\vec{g}_1 = \vec{0}$, and $X = X^*$, $X\vec{g}_1^* = \vec{0}$ as well, implying that $\vec{g}_1^* \in G$. Now define $G_1 = \text{span}\,\{\vec{g}_1, \vec{g}_1^*\}$ and $G_{-1} = G/G_1$, so that $G_1 \cup G_{-1} = \left\{\vec{0}\right\}$ and $G = G_1 \oplus G_{-1}$. If $\dim G_{-1} = 0$, stop. If $\dim G_{-1} > 0$ repeat this construction using $G_{-1}$ in place of $G$: i.e., choose any nonzero $\vec{g}_2 \in G_{-1}$, let $G_2 = \text{span}\,\{\vec{g}_2, \vec{g}_2^*\}$, define $G_{-2} = G_{-1}/G_2$, and continue this construction until, at some iteration $K$, $\dim G_{-K} = 0$. The result is a set of subspaces $\{G_k\}_{k=1}^K$, that have only trivial intersections and that partition $G$ so that

$$G = \bigoplus_{k=1}^{K} G_k. \tag{226}$$

For any complex vector $\vec{v} \in \mathbb{C}^M$, it is readily seen that $\text{span}\,\{\Re(\vec{v}), \Im(\vec{v})\} = \text{span}\,\{\vec{v}, \vec{v}^*\}$, where $\Re(\vec{v})$ and $\Im(\vec{v})$ are real vectors that respectively denote the real and imaginary parts of $\vec{v}$, i.e., $\vec{v} = \Re(\vec{v}) + i\Im(\vec{v})$. Now for each $k \in \{1, \ldots, K\}$, define a basis $\mathcal{B}_k$ for $G_k$ as follows:

- Set $\mathcal{B}_k = \{\Re(\vec{g}_k)\}$ if $\dim G_k = 1$ and $\Re(\vec{g}_k) \neq \vec{0}$,
- Set $\mathcal{B}_k = \{\Im(\vec{g}_k)\}$ if $\dim G_k = 1$ and $\Re(\vec{g}_k) = \vec{0}$,
- Set $\mathcal{B}_k = \{\Re(\vec{g}_k), \Im(\vec{g}_k)\}$ if $\dim G_k = 2$.

Finally, let $\mathcal{B} = \bigcup_{k=1}^K \mathcal{B}_k$. Observing that

$$\text{span}\,\mathcal{B} = \bigoplus_{k=1}^{K} \text{span}\,\mathcal{B}_k = \bigoplus_{k=1}^{K} G_k = G. \tag{227}$$

and

$$|\mathcal{B}| = \sum_{k=1}^{K} |\mathcal{B}_k| = \sum_{k=1}^{K} \dim G_k = \dim G, \tag{228}$$

we see that $\mathcal{B}$ is a real basis for $G$. This proves the claim. □

## References


[1] Anna Posfai, David M McCandlish, and Justin B Kinney. Gauge fixing for sequence-function relationships. *In prep.*, 2024.

[2] Bruce E Sagan. *The Symmetric Group: Representations, Combinatorial Algorithms, and Symmetric Functions*. Graduate Texts in Mathematics. Springer, 2 edition, 2001. Read in early 2022.

[3] Gary D Stormo. Maximally efficient modeling of DNA sequence motifs at all levels of complexity. *Genetics*, 187(4):1219 – 1224, 2011-04.
